# Supplementary material for: Digital health and the promise of equity in maternity care: A mixed methods multi-country assessment on the use of information and communication technologies in healthcare facilities in Latin America and the Caribbean
Source: PLoS One. 2024 Feb 27;19(2):e0298902. doi: 10.1371/journal.pone.0298902 (PMC10898739; doi:10.1371/journal.pone.0298902)
Supplement: S1 Table — (DOCX) [file pone.0298902.s001.docx]

**S1 Table. Sampling frame by country**

| **Country** | **Sampling universe** | **Facility universe** | **Data collection dates** | **Data collection strategy** |
| --- | --- | --- | --- | --- |
| Argentina | National. Public maternity hospitals throughout the republic | 114 | June 29 to August 19, 2021 | In charge of the Rosario Center for Perinatal Studies (CREP). Contacts through WhatsApp and email. |
| Bolivia | National. Public healthcare facilities that provide maternity care that have telemedicine focal points. | 344 | June 22 to August 12, 2021 | In charge of the National Telehealth Program of the Ministry of Health and Sports of Bolivia to all its institutional telehealth focal points. |
| Colombia | Two priority departments: Bogotá (urban area) and El Chocó (rural area). All public healthcare facilities registered in REPS was considered. | 212 | August 4 to September 1, 2022 | Coordinated by a local consultant, the survey was sent to network managers for dissemination among the establishments under their responsibility. Virtual and face-to-face follow-up. |
| Ecuador | All public facilities in two priority administrative zones: Zone 3, made up of the provinces of Cotopaxi, Tungurahua, Chimborazo, and Pastaza, and the province of Morona Santiago in Zone 6. | 370 | June 28 to August 4, 2021 | Through the zone coordinators of the Ministry of Public Health of zones 3 and 6, via email. |
| Guyana | National. 10 administrative districts. All private and public facilities that provide maternity care were considered. | 361 | August 7 to September 9, 2021 | Search by a consultant of all contacts, sending the link by WhatsApp and administration by telephone, in many cases, since several areas do not have an internet connection |
| Honduras | National. Public health facilities that provide maternal and child services in the 20 health regions. | 75 | June 24 to August 1, 2021 | Official letter issued by the Secretary of Health (SESAL) requesting each Head of the Regional (of the departments in which the Maternal and Child Service is provided) to collaborate in filling out said survey. Search by a consultant of all contacts, sending the link by WhatsApp, and telephone follow-up. |
| Paraguay | Nine of the 18 health regions of the country, Concepción, San Pedro, Amambay, Paraguarí, Canindeyú, Alto Paraná, Alto Paraguay, Boquerón and Presidente Hayes. Public and private sector health facilities that provide maternity care. | 631 | June 26 to August 4, 2021 | The list of services and providers was requested from each participating health region by a consultant, who contacted the institutions via WhatsApp. |
| Peru | National level. Distribution to obstetricians affiliated with the National College of Obstetricians of Peru. Includes private and public sector. | 446 | June 18 to August 31, 2021 | Through the National College of Obstetricians of Peru, which sent the regional coordinators from the central level, who in turn followed up with the members. |
| Dominican Republic | National. All public healthcare facilities under the coordination of the Maternal and Child Department. | 160 | July 23 to June 29, 2021 | Dissemination through the Maternal Health Coordinator, Maternal and Child Department, National Health Service, to the 9 regional directors of maternal health, with a request to be sent to the directors of health establishments. |
