# Supplementary material for: Digital health and the promise of equity in maternity care: A mixed methods multi-country assessment on the use of information and communication technologies in healthcare facilities in Latin America and the Caribbean
Source: PLoS One. 2024 Feb 27;19(2):e0298902. doi: 10.1371/journal.pone.0298902 (PMC10898739; doi:10.1371/journal.pone.0298902)
Supplement: S2 Table — (DOCX) [file pone.0298902.s002.docx]

| **S2 Table. Response rate by country.** | | | |
| --- | --- | --- | --- |
| **Country** | **Facility universe** | **Survey responses** | **Response rate** |
| **TOTAL** | **2642** | **1877** | **71%** |
| Argentina | 114 | 73 | 64% |
| Bolivia | 344 | 305 | 89% |
| Colombia* | 133 | 212 | 100% |
| Ecuador* | 370 | 402 | 100% |
| Guyana | 361 | 72 | 20% |
| Honduras | 83 | 83 | 100% |
| Paraguay | 631 | 260 | 41% |
| Peru | 446 | 431 | 97% |
| Dominican Republic | 160 | 39 | 24% |
| *Notes:* In Colombia, the facility list was developed based on the facilities listed on the national Registry of Healthcare Facilities (REPS) as facilities in the public sector with authorization to provide telemedicine and the survey was disseminated to others from these initial contacts. In Ecuador, there is a duplication of facilities as multiple providers completed the survey from the same site. | | | |
