# Supplementary material for: Digital health and the promise of equity in maternity care: A mixed methods multi-country assessment on the use of information and communication technologies in healthcare facilities in Latin America and the Caribbean
Source: PLoS One. 2024 Feb 27;19(2):e0298902. doi: 10.1371/journal.pone.0298902 (PMC10898739; doi:10.1371/journal.pone.0298902)
Supplement: S3 Table — (DOCX) [file pone.0298902.s003.docx]

| **S3 Table. Use of ICTs for abortion counseling by country** | |
| --- | --- |
| **Country** | **Use of ICT for abortion counselling** |
| Argentina | 20 (35.7) |
| Bolivia | 9 (3.6) |
| Colombia | 50 (29.6) |
| Dominican Republic | 2 (8.3) |
| Ecuador | 23 (8.8) |
| Guyana | 8 (16.3) |
| Honduras | 0 (0.0) |
| Paraguay | 12 (5.5) |
| Peru | 10 (2.5) |
| Total | 134 (9.0) |
