# Supplementary material for: Digital health and the promise of equity in maternity care: A mixed methods multi-country assessment on the use of information and communication technologies in healthcare facilities in Latin America and the Caribbean
Source: PLoS One. 2024 Feb 27;19(2):e0298902. doi: 10.1371/journal.pone.0298902 (PMC10898739; doi:10.1371/journal.pone.0298902)
Supplement: S1 Text — (DOCX) [file pone.0298902.s004.docx]

**S1 Text. Interview Guide**

**Interview Guidelines – civil servants or ministry officers, healthcare workers**

**Regional study on the use of digital strategies and telehealth to increase access to maternal health services during the COVID-19 pandemic in selected countries.**

Good morning/afternoon. My name is name, I am a consultant for the Latin American Center of Perinatology, Women and Reproductive Health of the Pan American Health Organization, World Health Organization. Thank you for your time and attention. We are conducting a regional study on the use of digital tools to ensure access to maternal health services during the COVOD-19 pandemic in 8 countries in Latin America and the Caribbean, which includes your country. This study is part of a collaborative research agreement between the Susan Thompson Buffet Foundation and PAHO, under the Technical Advice of Dr. Bremen de Mucio, member of the CLAP/PAHO and in collaboration with the PAHO’s national office.

The main objective is to identify which of the countries that are beneficiaries of this subsidy require technical and financial support to make progress in the field of telehealth processes for maternal health. The purpose of this interview is to learn about you view, as a civil servant/ministry officer/healthcare worker on the current situation of telehealth programs in the maternal health services, with an emphasis on antenatal care, and to find out more about promising experiences that could be replicated in other contexts.

The interview is based on a guided questionnaire and will take 20 minutes approximately. Information is confidential and findings will be presented globally, without revealing personal identification information. You may choose not to answer or to skip any question.

Do you have any question in regards to the interview or study? [wait for the answer]

Do you agree to participate? [wait for the answer; only continue if the answer is yes]

1. **Adoption of telehealth strategies in response to COVID-19 services for antenatal care**

1. Did you adopt any measures that considered or implies the use of digital tools or information and communication technology to provide maternal health services and reach pregnant women or new mothers during the pandemic? (such as modifying the organization of services, implementing a new national phone line for pregnant women, etc.?

2. Can you please describe any of these measures? Did you prioritize a certain region or specific sector of the population?

3. What specific ICTs tool did you use and how?

Below, you may find a few examples …

Launching of a new telephone line for pregnant women and new mothers to provide healthcare support, including WhatsApp

Creation of email addresses for qualified staff to respond to obstetric emergencies.

Development/use/adaptation of mobile applications (apps) to provide information via messages.

Portal and platform development for users.

Geolocation of pregnant women.

Budget allocation to provide remote patient monitoring devices (RPM) for women with a high risk pregnancy.

Development of mobile applications (apps) for remote patient monitoring (associated to devices).

Purchase/provision of mobile phones, phone and data cards for pregnant women to contact institutions by phone or email.

Remote access to patient information (medical records, laboratory results, X-rays, etc.)

Electronic information systems to share data with pharmacies, laboratories or other health care providers or organizations.

Virtual consultation platform or system.

Training of health professionals.

4. What kind of maternal health services were offered through ICTs methods? (contraceptive counseling, breastfeeding counseling, identification of risk indicators, support for references/counter references; inter-consultation system)

5. Do you have an electronic information system for pregnant women that allows remote access to medical records, laboratory results, X-rays, etc.?

6. Does this system have a security system to ensure confidentiality of information?

7. In your opinion, did the use of telehealth change after the COVID-19 pandemic?

8. Will you continue to use telehealth once the health emergency is solved?

1. **Funding**

Were budget resources allocated for the expansion of telehealth or the implementation of telehealth strategies? Was that a general initiative? Did it only apply to maternal or sexual and reproductive health?

Resources were only public, or was there any private funding?

Do you think the allocation of resources for telehealth was sufficient to meet the objectives?

**C. Facilitating factors**

**To what extent do you think the following factors have facilitated the use of ICT?**

Options: Barrier, did not facilitate, moderately facilitated, strongly facilitated

Health human resources trained in the use of ICT

PCs and connectivity.

Funding for telehealth services.

Level of evidence level for the effectiveness of the telehealth programs implemented.

Level of acceptance of the telehealth services by the health institutions.

National policies that recognize and regulate telehealth.

Quality of telehealth services.

Appropriate monitoring services.

Availability of qualified staff for IT technical support.

Services that are reachable, acceptable and accessible by the target population (just ask whether services were provided for women)

Lessons learned

1. What have been the main accomplishments in terms of the use of telehealth to provide remote maternal health care services?

2. What have been the three most important challenges in the use of technology to provide remote healthcare services?

3. What have been the main lessons learned during this process?

4. Did you use any impact measurement tool?

Apart from interviewing workers, we aim to identify promising practices in the use of maternal telehealth. Can you suggest any practice you implemented in connection with health services that may be deeply systematized?

Thank you for your participation. Your time and expertise are much appreciated. We will share the findings, lessons learned and conclusions with to you through the PAHO.
