# Supplementary material for: Digital health and the promise of equity in maternity care: A mixed methods multi-country assessment on the use of information and communication technologies in healthcare facilities in Latin America and the Caribbean
Source: PLoS One. 2024 Feb 27;19(2):e0298902. doi: 10.1371/journal.pone.0298902 (PMC10898739; doi:10.1371/journal.pone.0298902)
Supplement: S2 Text — (PDF) [file pone.0298902.s005.pdf]

A. Introducción

Centro Latinoamericano de Perinatología/Salud de la Mujer y Reproductiva

Encuesta para administradores y/o gerentes de establecimientos sanitarios sobre el uso de la telesalud para asegurar el acceso a los servicios de salud materna durante la pandemia de la COVID-19 en países seleccionados de las Américas

Información acerca del estudio

Agradecemos su interés en la presente encuesta regional sobre el uso de la telesalud para asegurar el acceso a los servicios de salud materna durante la pandemia de la COVID-19. El propósito es conocer cómo los establecimientos de salud usaron diversas herramientas digitales o tecnologías de la información y la comunicación (TICs) para adecuar los servicios de salud materna frente a la pandemia.

Esta encuesta está organizada por el Centro Latinoamericano de Perinatología/Salud de la Mujer y Reproductiva-Organización Panamericana de la Salud/Organización Mundial de la Salud (CLAP/SMR-OPS/OMS) en el marco del acuerdo entre dicha organización y la Fundación Susan Thompson Buffet. Desde ya agradecemos su tiempo y valiosos aportes.

La encuesta está dirigida a administradores, gerentes de programa y proveedores de salud que trabajan en establecimientos de salud públicos o privados que brinden servicios de salud materna en los siguientes países: Argentina, Bolivia, la República Dominicana, Ecuador, Guyana, Honduras, Paraguay y Perú. Se solicita que cada establecimiento asigne a una persona para completar la encuesta.

Se espera que los hallazgos sean un insumo para identificar qué países requieren apoyo técnico y financiero para avanzar en procesos de telesalud en el campo de la salud materna.

Los objetivos específicos son:

Conocer la situación de la oferta de programas de telesalud en los servicios de salud materna, con énfasis en la atención prenatal, en 8 países de las Américas. Identificar algunas experiencias prometedoras que pudieran ser replicables en países que lo necesitasen.

En promedio, le tomará unos 14 minutos completar la encuesta.

Para mayor información dirigirse a Ariadna Capasso, Consultora de CLAP-SMR-OPS/OMS, ariadnacapasso@gmail.com, con copia al Dr. Bremen de Mucio, Asesor Regional en Salud

Materna, CLAP-SMR-OPS/OMS, demuciob@paho.org y a Magdalena Bonasso, CLAP-SMR-OPS/OMS, bonassom@paho.org.

Consentimiento

La decisión de participar en esta encuesta es estrictamente voluntaria. No hay ninguna sanción por no participar. Usted tiene derecho a terminar su participación en cualquier momento cerrando la ventana del navegador. También tiene derecho a no contestar cualquier pregunta que no desee responder.

Su participación es totalmente confidencial. Los datos serán consolidados para el análisis; eliminaremos cualquier información de identificación personal de la base de datos y no colectaremos la dirección de IP. Los datos serán reportados globalmente sin utilizar información de identificación personal.

Entiendo y acepto los términos de mi participación arriba descritos.

- ○ Sí
- ○ No

Completo esta encuesta de forma voluntaria.

- ○ Sí
- ○ No

Instrucciones

Conteste cada pregunta siguiendo las instrucciones según corresponda. En las preguntas de múltiples opciones, existen dos tipos de formatos: aquellas en las que se puede seleccionar solo una opción (señaladas por redondeles) y aquellas en las que se pueden seleccionar múltiples opciones (señaladas por cuadrados). En algunas preguntas, verá la opción "Otros" con un espacio para completar según sea relevante (por ej. si las otras opciones no aplican).

B. Información general

¿En qué país brinda servicios de salud? (Seleccione una opción)

- ○ Argentina
- ○ Bolivia

- República Dominicana
- Ecuador
- Guyana
- Honduras
- Paraguay
- Perú

Indique el nombre de su establecimiento y la ciudad/pueblo donde está localizado

Nombre del establecimiento

Ciudad/pueblo

En su establecimiento ¿se utiliza alguna tecnología de la información y la comunicación (TIC) para la atención en salud materna? (Por ej., llamadas telefónicas, videollamadas, telemonitorización, mensajes de texto, etc.)

- Sí, de la misma manera que antes de la pandemia
- Sí, más que antes de la pandemia
- Sí, por primera vez a partir del inicio de la pandemia
- No

No usa TIC

¿Por qué considera que no se utilizaron TICs en los servicios de salud materna de su establecimiento? Por favor, clasifique en orden de importancia los motivos (siendo el 1 el motivo más importante y el 11 el menos importante)

Falta de recursos humanos para expandir la oferta de servicios.

Falta de equipo y/o conectividad en el establecimiento.

Falta de recursos financieros.

Falta de evidencia sobre la eficacia de los servicios brindados a través de la telesalud.

Falta de marco regulatorio (legislación, normativas).

Baja aceptabilidad de los servicios por el personal.

Baja aceptabilidad de los servicios por las mujeres gestantes.

No es una prioridad para las autoridades institucionales.

Calidad de los servicios de telesalud.

Baja accesibilidad de los servicios para la población meta (falta de equipo/conectividad de la población.)

Otros (por favor, especificar) [ ]

C. Uso de las tecnologías de la información y la comunicación (TIC)

¿Qué servicios de salud materna se ofrecen mediante el uso de la información y la comunicación (TIC)? (seleccione todas las opciones que correspondan)

- ☐ Controles prenatales de rutina
- ☐ Controles prenatales para diagnóstico de enfermedades (preclamsia, diabetes gestacional, etc.)
- ☐ Controles prenatales para manejo de enfermedades (preclamsia, diabetes gestacional, etc.)
- ☐ Asesoramiento para la interrupción legal o voluntaria del embarazo (según la legislación existente)
- ☐ Prescripción de medicamentos (con receta digital o que no requieran receta)
- ☐ Preparación para el parto
- ☐ Atención postnatal de rutina
- ☐ Telemonitorización de enfermedades a través de la distribución de dispositivos para el autocuidado y monitoreo remoto de enfermedades crónicas o específicas del embarazo durante el embarazo
- ☐ Telemonitorización de enfermedades a través de la distribución de dispositivos para el autocuidado y monitoreo remoto de enfermedades crónicas o específicas del embarazo durante el embarazo posparto
- ☐ Asesoramiento de lactancia materna
- ☐ Asesoramiento de planificación familiar y/o métodos anticonceptivos
- ☐ [ ] Otros (por favor, describir)

¿Qué estrategias digitales y/o tecnologías de la información y comunicación se utilizaron? (seleccione todas las opciones que apliquen)

- ☐ Consultas telefónicas (de línea o por WhatsApp o aplicación similar)
- ☐ Consultas por videollamadas (por Zoom, Skype, o similar)

- ☐ Habilitación de una línea telefónica de asistencia sanitaria, incluido por WhatsApp, para las mujeres gestantes
- ☐ Aplicaciones móviles (apps) para enviar mensajes con consejos y recordatorios
- ☐ Provisión de dispositivos de telemonitorización a las mujeres con embarazos de alto riesgo
- ☐ Aplicaciones móviles (apps) para la telemonitorización de pacientes a distancia (ligados a los dispositivos)
- ☐ Provisión de teléfonos móviles, tarjetas o servicio de datos para los móviles para que las gestantes puedan comunicarse con la institución por teléfono o correo electrónico
- ☐ Habilitación de correos electrónicos para emergencias obstétricas con personal dedicado
- ☐ Acceso a información del paciente (historias médicas, resultados de laboratorio, radiografías, etc.) de forma remota
- ☐ Sistema de información electrónico para compartir datos con farmacias, laboratorios u otros profesionales de la salud
- ☐ ________ Otras (por favor, especificar)

¿Aproximadamente, qué porcentaje de las consultas de atención en salud materna se cubren actualmente utilizando telesalud?

- ○ Ninguna
- ○ Pocas - menos del 25%
- ○ Algunas - entre el 25% y el 50%
- ○ Muchas - entre el 50% y el 75%
- ○ La mayoría - más del 75%

¿Su establecimiento planea seguir utilizando las estrategias digitales y/o tecnologías de la información y comunicación para llegar a las mujeres gestantes una vez resuelta la emergencia sanitaria?

- ○ Definitivamente sí
- ○ Probablemente sí
- ○ No sé
- ○ Probablemente no
- ○ Definitivamente no

D. Éxitos, barreras y lecciones aprendidas

¿En qué medida considera que los siguientes factores han facilitado el uso de las TIC?

	No ha facilitado	Ha facilitado moderadamente	Ha facilitado mucho
Recursos humanos capacitados en el uso de TIC.	○	○	○
Equipo y/o conectividad.	○	○	○
Financiamiento para los servicios de telesalud.	○	○	○
Nivel de la evidencia sobre la eficacia de los programas de telesalud implementados.	○	○	○
Protocolos institucionales de telesalud.	○	○	○
Nivel de aceptación de los servicios de telesalud por los proveedores.	○	○	○
Políticas nacionales que reconocen y reglamentan la telesalud.	○	○	○
Calidad de los servicios de telesalud.	○	○	○
Sistemas de supervisión adecuados para teleconsultas.	○	○	○
Disponibilidad de personal capacitado en informática para resolver problemas técnicos.	○	○	○

¿En qué medida considera que los siguientes factores relacionados a la población meta han facilitado llegar a las usuarias a través del uso de las TIC?

	No ha facilitado	Ha facilitado moderadamente	Ha facilitado mucho
Nivel de demanda por parte de las usuarias.	○	○	○
Nivel de aceptación de los servicios de telesalud por las usuarias.	○	○	○

	No ha facilitado	Ha facilitado moderadamente	Ha facilitado mucho
Nivel de accesibilidad de las usuarias a los servicios de telesalud (disponibilidad de equipo/conectividad.)	○	○	○
Costos de los servicios para las usuarias.	○	○	○
Adecuación de los servicios para las usuarias (aspectos culturales, lingüísticos, etc.)	○	○	○

¿Se utilizó alguna herramienta para evaluar el uso de las tecnologías de la información y la comunicación (TIC) en los servicios de salud materna?

- ○ Sí
- ○ No

E. Apoyo al personal

¿Se capacitó al personal de salud sobre cómo brindar atención de calidad de forma remota a través de la tecnología?

- ○ Sí
- ○ No

¿Su institución le brindó algún tipo de apoyo técnico o material a los proveedores de salud para la implementación de la telesalud? Por favor, seleccione todas las opciones que correspondan.

- ☐ Se designó un punto focal para consultas sobre telesalud para los profesionales de la salud
- ☐ Se contrató o designó a una persona capacitada en informática para resolver problemas técnicos
- ☐ Se distribuyeron teléfonos u otros dispositivos móviles o se cubrió el costo del servicio de datos para los proveedores de salud que brindan teleconsultas
- ☐ Se cubrieron los gastos de internet en los domicilios de los profesionales de la salud que brindan teleconsultas
- ☐ ________ Otro (por favor, especificar)

F. Protocolos de telesalud

¿Tiene su institución un reglamento o protocolo de telesalud?

- ◯ [ ] Sí. Año de adopción
- ◯ No

El reglamento o protocolo, ¿hace mención específica a la salud materna?

- ◯ Sí
- ◯ No

Si lo tiene disponible, por favor adjunte el reglamento o protocolo institucional de telesalud.

¿Se modificó el modelo de atención prenatal durante la pandemia?

- ◯ Sí
- ◯ No

G. Financiación

¿Recibió su institución financiación adicional específica para expandir los servicios de telesalud?

- ◯ Sí
- ◯ No

¿La financiación adicional fue otorgada durante la pandemia de la COVID-19?

- ◯ Sí
- ◯ No

La financiación fue principalmente (por favor, seleccione todas las opciones que correspondan)

- [ ] Pública (del gobierno nacional, estatal/provincial, o municipal/local)
- [ ] Privada (de empresas con fines de lucro)
- [ ] Privada (de donantes sin fines de lucro)
- [ ] Público/privada (de colaboraciones o programas co-financiados con fondos públicos y privados)

H. Gerencia de datos y confidencialidad

¿Cuenta su institución con un sistema de información electrónica de las gestantes que permita el acceso remoto a las historias clínicas, resultados de laboratorios, radiografías, etc.?

- ○ Sí
- ○ No

¿Existen sistemas de seguridad para asegurar la confidencialidad de las comunicaciones con las gestantes?

- ○ Sí
- ○ No

I. Preguntas generales acerca de la institución

Por favor, complete las siguientes preguntas acerca de su institución.

Tipo de institución (Seleccione una opción)

- ○ Hospital de referencia
- ○ Hospital regional o de distrito
- ○ Policlínica
- ○ Clínica o centro de salud
- ○ Puesto de salud o Unidades de Salud de la Familia
- ○ Casa materna
- ○ Red de salud
- ○ __________ Otro (especificar):

¿Cuál es el nivel de atención de su institución? (Seleccione una opción)

- ○ Primer nivel de atención
- ○ Segundo nivel de atención
- ○ Tercer nivel de atención

¿Qué tipo de institución es? (Seleccione una opción)

- ○ Pública (nacional o provincial/estatal)
- ○ Pública (académica o docente)
- ○ Pública (departamental, municipal o nivel más pequeño)
- ○ Privada con fines de lucro
- ○ Privada (académica o docente)
- ○ Privada (religiosa)
- ○ No gubernamental (sin fines de lucro)
- ○ ________ Otro (especificar)

¿Cuál es el área de captación a la que brinda servicios su institución? (Seleccione una opción)

- ○ Ciudad o municipio grande (>1 millón de habitantes)
- ○ Ciudad o municipio mediano (100 000 a 1 millón habitantes)
- ○ Ciudad o municipio pequeño (<100 000 habitantes)
- ○ Pueblo o zona rural
- ○ Campo de refugiados o de personas desplazadas
- ○ ________ Otra (especificar)

Aproximadamente, en promedio, pensando en los años 2019-2020, ¿qué porcentaje de la población de gestantes que se atiende en su institución...? (Las opciones no suman a 100%)

	0% a 100%
Es migrante (nacida en otro país)	☐
No habla el idioma nativo	☐
Vive bajo el nivel de pobreza	☐
Es de ascendencia indígena	☐

	0% a 100%
Es afrodescendiente	

J. Preguntas generales

¿Cuál es su cargo actual? (Seleccione una opción)

- Gerente de la institución (director/a, administrador/a)
- Jefa/e de servicio o departamento
- Jefa/e de equipo
- Miembro de equipo
- Miembro interino
- Otro (especificar):

K. Contacto

Gracias por su participación. Valoramos mucho su tiempo y experiencia. Nos gustaría contar con su información de contacto por si tenemos alguna pregunta de seguimiento. Si está de acuerdo con que nos comuniquemos con usted acerca del estudio en el futuro, por favor, ingrese su correo electrónico

Indique si desea que compartamos información acerca de los resultados del estudio con usted

- Sí
- No

Powered by Qualtrics
