# Supplementary material for: Digital health and the promise of equity in maternity care: A mixed methods multi-country assessment on the use of information and communication technologies in healthcare facilities in Latin America and the Caribbean
Source: PLoS One. 2024 Feb 27;19(2):e0298902. doi: 10.1371/journal.pone.0298902 (PMC10898739; doi:10.1371/journal.pone.0298902)
Supplement: S1 Graph — (DOCX) [file pone.0298902.s006.docx]

**S1 Graph. Future use of ICTs among respondents who used them (n = 1459)**
