# Supplementary material for: Digital health and the promise of equity in maternity care: A mixed methods multi-country assessment on the use of information and communication technologies in healthcare facilities in Latin America and the Caribbean
Source: PLoS One. 2024 Feb 27;19(2):e0298902. doi: 10.1371/journal.pone.0298902 (PMC10898739; doi:10.1371/journal.pone.0298902)
Supplement: Attachment — Submitted filename: PONE-D-23-21638_R1_reviewer.pdf [file pone.0298902.s008.pdf]

PLOS ONE

Digital health and the promise of equity in maternity care: A mixed methods multi-country assessment on the use of information and communication technologies in healthcare facilities in Latin America and the Caribbean

--Manuscript Draft--

**Manuscript Number:**	PONE-D-23-21638R1
**Article Type:**	Research Article
**Full Title:**	Digital health and the promise of equity in maternity care: A mixed methods multi-country assessment on the use of information and communication technologies in healthcare facilities in Latin America and the Caribbean
**Short Title:**	Digital health in maternity care in Latin America and the Caribbean
**Corresponding Author:**	Ariadna Capasso<br>Health Resources in Action<br>UNITED STATES
**Keywords:**	Digital Health; Maternity care; Obstetrics and Gynecology; Latin America and the Caribbean; LMIC
**Abstract:**	Introduction: Timely access to maternity care is critical to saving lives. Digital health may serve to bridge the care chasm and advance health equity. Conducted in the aftermath of the COVID-19 pandemic, this cross-sectional mixed-methods study assessed the use of information and communication technologies (ICTs) in healthcare facilities in nine Latin American and Caribbean countries to understand the landscape of ICT use in maternity care and the barriers and facilitators to its adoption.<br><br>Materials and methods: Between April 2021 and September 2022, we disseminated an online survey in English and Spanish among, mainly public, healthcare institutions that provided maternity care in Argentina, Bolivia, Colombia, the Dominican Republic, Ecuador, Guyana, Honduras, Paraguay and Peru. We also interviewed 27 administrators and providers in ministries of health and healthcare institutions.<br><br>Results: Most of the 1877 institutions that answered the survey reported using ICTs in maternity care (N=1536, 82%), ranging from 96% in Peru to 64% in the Dominican Republic. Of institutions that used ICTs, 59% reported using them more than before or for the first time since the pandemic began. ICTs were most commonly used to provide family planning (64%) and breastfeeding (58%) counseling, mainly by phone (82%). At the facility level, availability of equipment and internet coverage, coupled with skilled human resources, were the main factors associated with ICT use. At country level, government-led initiatives to develop digital health platforms, alongside national investments in the digital infrastructure, were the determining factors in the adoption of ICTs in healthcare provision.<br><br>Conclusion: Digital health for maternity care provision relied on commonly available technology and did not necessitate highly sophisticated systems, making it a sustainable and replicable strategy. However, disparities in access to digital health remain and many facilities in rural and remote areas lacked connectivity. Use of ICTs in maternity care depended on countries' long-term commitments to achieving universal health and digital coverage.
**Order of Authors:**	Ariadna Capasso
	Mercedes Colomar
	Dora Ramírez
	Suzanne Serruya
	Bremen de Mucio
**Response to Reviewers:**	PONE-D-23-21638<br>Digital health and the promise of equity in maternity care: A mixed methods multi-country assessment on the use of information and communication technologies in

Powered by Editorial Manager® and ProduXion Manager® from Aries Systems Corporation

healthcare facilities in Latin America and the Caribbean
Point-by-point response to reviewers
Journal Requirements
1.Please ensure that your manuscript meets PLOS ONE's style requirements, including those for file naming. The PLOS ONE style templates can be found at https://journals.plos.org/plosone/s/file?id=wjVg/PLOSOne_formatting_sample_main_body.pdf and https://journals.plos.org/plosone/s/file?id=ba62/PLOSOne_formatting_sample_title_authors_affiliations.pdf
RESPONSE: We modified the manuscript in accordance to PLOS ONE's style requirements.
2. Thank you for stating in your Funding Statement:
"This study was partially funded by Global Affairs Canada through an agreement with the Pan American Health Organization/the World Health Organization. Global Affairs Canada was not involved in the study design, collection, analysis, and interpretation of data, writing, and submission."
RESPONSE: We included an amended statement in the cover letter, where we also confirm that no additional funding was received for this study.
3. In your Data Availability statement, you have not specified where the minimal data set underlying the results described in your manuscript can be found. PLOS defines a study's minimal data set as the underlying data used to reach the conclusions drawn in the manuscript and any additional data required to replicate the reported study findings in their entirety. All PLOS journals require that the minimal data set be made fully available. For more information about our data policy, please see http://journals.plos.org/plosone/s/data-availability.
Upon re-submitting your revised manuscript, please upload your study's minimal underlying data set as either Supporting Information files or to a stable, public repository and include the relevant URLs, DOIs, or accession numbers within your revised cover letter. For a list of acceptable repositories, please see http://journals.plos.org/plosone/s/data-availability#loc-recommended-repositories. Any potentially identifying patient information must be fully anonymized.
We will update your Data Availability statement to reflect the information you provide in your cover letter.

RESPONSE: The minimal data set has been uploaded to the OpenICPSR repository, and can be found in repository number: https://doi.org/10.3886/E195667V1.
4. Please include captions for your Supporting Information files at the end of your manuscript, and update any in-text citations to match accordingly. Please see our Supporting Information guidelines for more information: http://journals.plos.org/plosone/s/supporting-information.

RESPONSE: Separate supporting information files have been uploaded for each element, the in-text citations have been modified to meet PLOS ONE formatting standards, and a list of captions have been included at the end of the manuscript file.

Review Comments to the Author
Please note, the line numbers listed below are those in the revised manuscript with tracked changes document.
5. Review Comments to the Author
Reviewer #1: The manuscript described a very interesting issue in health area and approached a promissing issue in the present and future time. The main objective is describing the use of information and communication technology in maternal care assistence in nine Latin American countries.

There are some questions to the authors, which are:
-Which was the criteria to select these countries? Why was Brazil not included, as it is one of the biggest countries in Latin America?
RESPONSE: This is an important point as Brazil is the country with the highest absolute number of maternal deaths in the region. However, this study was part of a Pan American Health Organization program to improve access to primary care in Latin America and the Caribbean and only included the countries in which this program was implemented.
We addressed this in the methods (new text in italics):
"This is a cross-sectional mixed methods study of health facilities providing maternity

	care in 9 Latin American and Caribbean countries that participated in a PAHO-led program to improve primary care access" (lines 163-165)<br><br>-Is there any specific reason to explain the difference of response rate among countries, beyond internet affordability?<br>RESPONSE: Difference in response rates among countries probably depends on the survey dissemination strategy, as well as on internet coverage and the accessibility of equipment. I think the manuscript makes the case that a State that has historically invested in developing a digital infrastructure - as well as a robust public healthcare system – is key to ICT use, in general.<br><br>-Is there a difference in the use of ICT between the public and private service? What is the percentage of public and private services in each country?<br>RESPONSE: This is a great question. Private services probably do have a more developed ICT infrastructure because they have more resources. For example, in Peru, members of EsSalud, which is the government's paid health insurance for employed citizens, have more options to access telemedicine, than members of SIS, which is the government's free health insurance. This manuscript primarily focused on free public services for the most vulnerable populations who face the greatest difficulties accessing health care. Each country's health care systems is pretty fragmented. Determining the percentage of public versus public services available in each would be beyond the scope of this manuscript. However, it would be an interesting future study.<br><br>-Page 8: The authors described that "in 7 countries, interviews were conducted with maternal health...". The question is: in the other 2 countries, with whom the interview was conducted?<br>RESPONSE: This is a great question. We did not conduct qualitative data collection in two countries. We only conducted interviews in countries where we learned about programs in maternity care that used ICTs, so that we could gather more details about these.<br>We clarified this point in line 238 (added text in italics):<br>"In the 7 countries where we found promising examples of use of ICTs in maternity care…"<br><br>-In table 1 (page 12 and 13): why the authors included the column "Used ICTs (%by column)"? I suggest to exclude this column, as there are marked differences between the categories of each item (institution type, level of care and country).<br>RESPONSE: Thank you for this great suggestion. We removed the column.<br><br>-Review the numering of tables – there are 2 tables numbered as "1".<br>RESPONSE: We were referring to Table 1 twice, the first time to describe the types of institutions that responded to the survey and the other to describe the prevalence of ICT use. Since we modified the table and added a figure (new Fig 1), this is no longer an issue.<br>Reviewer #2: This is a good article including a massive survey conducted in the region. Please consider the following comments, that I structured in general and specific:<br><br>General comments<br>1. I didn't understand if the NYU IRB actually reviewed your submission and said it didn't need ethics approval, or if this was the general instruction that appear in its website. It does look that the informed consents that were used should have been approved by an ethics board.<br>RESPONSE: Thank you for this point. As per NYU publicly-available ethics committee guidelines, this study did not require submission to the IRB as it did not constitute human subjects' research. The information collected is not about individuals, but about organizations. The NYU IRB guidance is found here: Does Your Project Require an Application to the NYU IRB Office? Decision Tree #1. Available at: https://www.nyu.edu/content/dam/nyu/research/documents/IRB/IRBDecisionTree.pdf. Even though it didn't required IRB review, we followed ethical standards by using the consent to inform participants about how the information would be used.<br><br>2. I suggest the authors might review the English, and perhaps asking a native speaker to take a quick look at it. There are several examples in which the English could be

Powered by Editorial Manager® and ProduXion Manager® from Aries Systems Corporation

improved (e.g., “maternal mortality is a grave public health”).
RESPONSE: Thank you for your feedback. We have carefully copy edited the manuscript to ensure that there are no typos or grammatical errors.

3. The research questions and how the methods address them are a bit unclear from my perspective. RQ1 and 3 seems to be served by the survey and interviews, but it is not clear how RQ2 would be served by both methods.
RESPONSE: We included multiple questions both in the survey and interviews about the context in which ICTs were adopted, including when they were funded and adopted, among others, to help us understand if and how changes in ICT use occurred during COVID-19.

4. Additionally, it is not clear how the methods would be providing complementary perspectives to address the same questions (e.g., what is the difference of addressing RQ1 using the survey as opposed to using the interview results).
RESPONSE: The methods are complementary because whereas the survey allowed us to reach more institutions across the region and quantify the use of ICTs in the 9 countries assessed, the qualitative data allowed us to gather more nuanced information related to the impetus for ICT adoption, the context in which the adoption of ICTs occurred, the exact use of ICTs (impetus, team involved, intervention characteristics, etc.).
We explained this in lines 248-251:
“The information gathered from the different data collection methods was complementary: whereas the survey allowed us to collect quantitative data from many institutions, the qualitative data provided us nuanced information on the context in which specific interventions were implemented.”

5. It would be important to specify a bit more RQ2, and what is understood by “shape the implementation”
RESPONSE: We appreciate the point and have modified the language in RQ2 to address this point (rephrased RQ2 in italics, line 156):
1)Understand changes in the use of ICTs as a result of the COVID-19 pandemic

6. I was completely confused when the CFIR part came in the methods, and the results/discussion. If you think you could structure the whole research (including the questionnaire, interview guide and key findings) using CFIR, then please do so. But how it is now, it seems a different paper, because it is not clear what research question is being answered with that analysis.
RESPONSE: We made significant changes to the introduction and discussion to address this issue; we reorganized the discussion and removed the sections related to the CFIR framework. Whereas we still believe that CFIR provides a useful framework to understand the adoption of innovations in a real-world setting, we agree that this content would best suit a separate paper.

7. By addressing the previous comment, you could also reduce the length of the discussion section which is too long for a paper.
RESPONSE: Thank you for the suggestion. Indeed, the manuscript is much shorter with the removal of the CFIR sections.

8. In the classification of institutions type, I am not sure about the difference between them (e.g., is a referral hospital not a health center?)
RESPONSE: A health center is a health facility that only provides primary level care, as compared to a hospital which can offer higher complexity health services. To avoid confusion, we clarified this point by changing the terminology to “primary care health center.”

9. One thing that wasn’t clear to me is how use of ICTs is measured. It might be important to clearly state that in the methods section as well as reflecting around this in the discussion section.
RESPONSE: ICT use was measured by the following survey question:
Does your facility use any information and communication technology (ICT) tools for maternal health care (e.g., phone calls, video calls, telemonitoring, text messaging, etc.)?
The answer options were: Yes, the same way as we did before the onset of the

pandemic (1); Yes, more than before the pandemic (2); Yes, we started using ICT tools when the pandemic was already under way (3); and No (4).
We added this information to the methods:
"ICT use was assessed by the question "Does your facility use any information and communication technology (ICT) tools for maternal health care (e.g., phone calls, video calls, telemonitoring, text messaging, etc.)?" (Answer options: Yes, the same way as we did before the onset of the pandemic; Yes, more than before the pandemic; Yes, we started using ICT tools when the pandemic was already under way; and No). The variable was dichotomized Yes/No." (lines 215-219)

10. I got confused in page 24 for the "institutional factors and strategies". I don't understand how they are not a subset of factors that are explainednin figure 1?
RESPONSE: This confusion is understandable. Figure 1 presented survey respondents' perceptions of the factors that facilitated ICT use, whereas Table 3 are the actual strategies implemented by institutions to facilitate ICT use. To avoid confusion, we incorporated findings from the "institutional factors and strategies" section into the factors associated with ICT use.

Specific comments
Introduction
1. Not sure if eradicating maternal deaths is actually an objective. I tend to believe that the verb eradicating is normally used with diseases. Maybe it might be more relevant to use reducing maternal deaths.
RESPONSE: Thank you. We agree with the comment and changed the language to reducing. (line 67)

2. At the end of introduction, the COVID-19 pandemic is introduced at the end of this section, but seems very disconnected from the rest of the section.
RESPONSE: We appreciate this suggestion. We wove in the introduction to COVID-19 within the section presenting prior evidence on the use of ICTs in the region. The main point we were trying to make was that by creating barriers to accessing services in person, COVID-19 may have accelerated the adoption of ICTs. Thus, warranting this study on the use of ICTs in the post-pandemic period (lines 121-125).

3. It is important the the introducxtion could justify the research questions included. Reading the RQ1, it seems that could have been already answered by reference 14. What additional value is being added by this research piece by answering that research question?
RESPONSE: Reference 14 is a desk review of published evidence on the use of ICTs in sexual and reproductive health. However, the vast majority of health interventions carried out by health facilities in Latin America and the Caribbean are not documented. Thus, any study based solely on desk review is vastly limited. Primary data collection via survey and interviews provides a more robust picture of the situation of ICT use in the region.
We clarified this in the manuscript:
"A caveat of this study is that, since the majority of interventions carried out in facilities across Latin America and the Caribbean are not documented in the literature, a desk review provides only a partial picture of the reality on the ground.." (lines 138-141)

4. Additionally, authors should provide a more robust explanation of the data integration part of the mixed-methods. Are you using triangulation, what type of strategy?
RESPONSE: The survey findings were used to present quantitative data, primarily related to prevalence of ICT use, types of ICTs used, and services provided. The interviews served to learn more about specific practices and the context in which ICTs were used. As mentioned above, the methods are complementary because whereas the survey allowed us to reach more institutions and quantify the use of ICTs, the qualitative data allowed us to gather more nuanced information related to the impetus for ICT adoption, the context in which the adoption of ICTs occurred, the exact use of ICTs (purposes, technology used, team involved, etc.).
We explained this in lines 248-251:
"The information gathered from the different data collection methods was complementary: whereas the survey allowed us to collect quantitative data from many institutions, the qualitative data provided us nuanced information on the context in

Powered by Editorial Manager® and ProduXion Manager® from Aries Systems Corporation

which specific interventions were implemented."

5. It would be important the the authors describe why these nine countries were selected, and why only in 7 were the interviews conducted.
RESPONSE: This is a great point. This study was part of a Pan American Health Organization program to improve access to primary care in Latin America and the Caribbean and only included the countries in which this program was implemented. We only conducted interviews in countries where we learned about programs in maternal health that used ICTs, so that we could learn more about these.
We addressed this in the methods (new text in italics):
"This is a cross-sectional mixed methods study of health facilities providing maternity care in nine Latin American and Caribbean countries that participated in a PAHO-led program to improve primary care access" (lines 164-165)
"In the 7 countries where we found promising examples of use of ICTs in maternal health care…" (line 238-239)

6. Reference 13 is not a review, as it is mentioned in page 6 line 124.
RESPONSE: Thank you for the observation. We removed that sentence and reworded that paragraph to better reflect the evidentiary sources. (lines 129-134)

Methods
1. I suggest the authors use clear subheadings following participants selection, data collection methods and data analysis, and clearly splitting the two parts of their study.
RESPONSE: Thank you for the suggestion. We separated the quantitative and qualitative descriptions of the study methods into sections.
2. Also, I believe this is a mixed-methods study that includes a cross-sectional study as a quantatitve component.
RESPONSE: This is both a mixed-methods study because it uses quantitative and qualitative data collection methods AND a cross-sectional study because there is no repeating or longitudinal data – data from each resondents was collected at one time point.

3. Page 8, line 159. Please use another paragraph when "in 7 countries" starts.
RESPONSE: This section was moved when we reorganized the methods. We made sure it starts a new paragraph. (line 238)

4. Could you please explain why you had duplicates and a confidence interval for the response rate?
RESPONSE: We had duplicates because some respondents started the online survey and stopped for one reason or another (e.g., their internet was cut, they were interrupted by something else), and then instead of continuing the survey, they restarted it. This only happened when they used a different device to try to complete the survey. We calculated the confidence interval of the mean response rate to account for random variance across countries.
We clarified: "Duplicates occurred when respondents continued working on unsubmitted surveys from a different device." (lines 275-276)

5. Page 9, lines 190-192, all these type of information I am inclined to say that it is part of the results.
RESPONSE: These lines were moved to the beginning of the results section. (lines 275-279)

Results
1. Page 11 line 238: There is an error.
RESPONSE: Thank you for pointing this out. We changed to the actual percentage, 76%. (line 302)

2. I would suggest that one key issue mentioned is that 78% of the institutions were primary care, meaning that we don't have much information about the uses of digital health in hospital.
RESPONSE: Thank you. We have included this point in the discussion.

3. Table 1 title: Please specify that these are the results from the survey.
RESPONSE: The title of the table was modified to include that these were the results

Powered by Editorial Manager® and ProduXion Manager® from Aries Systems Corporation

	from the survey (lines 304).<br><br>4. In table 1, I think the % by row is confusing because this is not a representative sample.<br>RESPONSE: Thank you for your suggestion. We removed the percentage by row column.<br><br>5. Table numbers are wrong.<br>RESPONSE: We were referring to Table 1 twice, the first time to describe the types of institutions that responded to the survey and the other to describe the prevalence of ICT use. Since we modified the table and added a figure (new Fig 1), this is no longer an issue.<br><br>6. In table 2, it might be good to have more details on the intersection between the 2 variables shown (e.g., what type of ICT was used for gestational disease management?).<br>RESPONSE: This is a good suggestion but cannot be answered with the current data. We could do crosstabs on the two variables, but that still wouldn't tell us specifically what types of ICTs were used for a specific condition such as gestational disease management.<br><br>7. Page 17, line 314-315. I think this is should be included in the methods<br>RESPONSE: Thank you for the suggestion. We moved this to the survey questionnaire section. (lines 215-217)<br><br>8. Table 3, could you also provide frequencies?<br>RESPONSES: Thank you the suggestion. We added the frequencies to Table 3.<br><br>Discussion<br>1. I didn't see strengths, while your survey was massive!<br>RESPONSE: Thank you! We added strengths to the Strengths and Limitations subsection (line 780).<br><br>2. I didn't see implications for practice either.<br>RESPONSE: Valuable point. We added a section on implications for practice in the discussion (starting in line 813).
**Additional Information:**	
**Question**	**Response**
**Financial Disclosure**<br><br>Enter a financial disclosure statement that describes the sources of funding for the work included in this submission. Review the submission guidelines for detailed requirements. View published research articles from *PLOS ONE* for specific examples.<br><br>This statement is required for submission and **will appear in the published article** if the submission is accepted. Please make sure it is accurate.	This study was partially funded by Global Affairs Canada through an agreement with the Pan American Health Organization/the World Health Organization. Global Affairs Canada was not involved in the study design, collection, analysis, and interpretation of data, writing, and submission.

**Unfunded studies**<br>Enter: *The author(s) received no specific funding for this work.*<br><br>**Funded studies**<br>Enter a statement with the following details:<br>• Initials of the authors who received each award<br>• Grant numbers awarded to each author<br>• The full name of each funder<br>• URL of each funder website<br>• Did the sponsors or funders play any role in the study design, data collection and analysis, decision to publish, or preparation of the manuscript?<br>• **NO** - Include this sentence at the end of your statement: *The funders had no role in study design, data collection and analysis, decision to publish, or preparation of the manuscript.*<br>• **YES** - Specify the role(s) played.<br><br>* typeset	
**Competing Interests**<br><br>Use the instructions below to enter a competing interest statement for this submission. On behalf of all authors, disclose any competing interests that could be perceived to bias this work—acknowledging all financial support and any other relevant financial or non-financial competing interests.<br><br>This statement is required for submission and **will appear in the published article** if the submission is accepted. Please make sure it is accurate and that any funding sources listed in your Funding Information later in the submission form are also declared in your Financial Disclosure statement.<br><br>View published research articles from *PLOS ONE* for specific examples.	The authors have declared that no competing interests exist.

Powered by Editorial Manager® and ProduXion Manager® from Aries Systems Corporation

**NO authors have competing interests**<br><br>Enter: *The authors have declared that no competing interests exist.*<br><br>**Authors with competing interests**<br><br>Enter competing interest details beginning with this statement:<br><br>*I have read the journal's policy and the authors of this manuscript have the following competing interests: [insert competing interests here]*<br><br>* typeset	
**Ethics Statement**<br><br>Enter an ethics statement for this submission. This statement is required if the study involved:<br><br>• Human participants<br>• Human specimens or tissue<br>• Vertebrate animals or cephalopods<br>• Vertebrate embryos or tissues<br>• Field research<br><br>Write "N/A" if the submission does not require an ethics statement.<br><br>General guidance is provided below. Consult the submission guidelines for detailed instructions. **Make sure that all information entered here is included in the Methods section of the manuscript.**	The New York University Institutional Review Board (IRB) does not consider the present study human subjects research because the information collected is not about individuals but about methods, policies, procedures, and organizations, and does not require application to the IRB (See NYU IRB Decision Tree found here: https://www.nyu.edu/content/dam/nyu/research/documents/IRB/IRBDecisionTree.pdf).

Powered by Editorial Manager® and ProduXion Manager® from Aries Systems Corporation

**Format for specific study types**<br><br>**Human Subject Research (involving human participants and/or tissue)**<br>• Give the name of the institutional review board or ethics committee that approved the study<br>• Include the approval number and/or a statement indicating approval of this research<br>• Indicate the form of consent obtained (written/oral) or the reason that consent was not obtained (e.g. the data were analyzed anonymously)<br><br>**Animal Research (involving vertebrate animals, embryos or tissues)**<br>• Provide the name of the Institutional Animal Care and Use Committee (IACUC) or other relevant ethics board that reviewed the study protocol, and indicate whether they approved this research or granted a formal waiver of ethical approval<br>• Include an approval number if one was obtained<br>• If the study involved *non-human primates*, add *additional details* about animal welfare and steps taken to ameliorate suffering<br>• If anesthesia, euthanasia, or any kind of animal sacrifice is part of the study, include briefly which substances and/or methods were applied<br><br>**Field Research**<br><br>Include the following details if this study involves the collection of plant, animal, or other materials from a natural setting:<br>• Field permit number<br>• Name of the institution or relevant body that granted permission	
**Data Availability**<br><br>Authors are required to make all data underlying the findings described fully available, without restriction, and from the time of publication. PLOS allows rare exceptions to address legal and ethical concerns. See the PLOS Data Policy and FAQ for detailed information.	Yes - all data are fully available without restriction

A Data Availability Statement describing where the data can be found is required at submission. Your answers to this question constitute the Data Availability Statement and **will be published in the article**, if accepted.<br><br>**Important:** Stating 'data available on request from the author' is not sufficient. If your data are only available upon request, select 'No' for the first question and explain your exceptional situation in the text box.<br><br>Do the authors confirm that all data underlying the findings described in their manuscript are fully available without restriction?	
**Describe where the data may be found in full sentences. If you are copying our sample text, replace any instances of XXX with the appropriate details.**<br><br>• If the data are **held or will be held in a public repository**, include URLs, accession numbers or DOIs. If this information will only be available after acceptance, indicate this by ticking the box below. For example: *All XXX files are available from the XXX database (accession number(s) XXX, XXX.).*<br>• If the data are all contained **within the manuscript and/or Supporting Information files**, enter the following: *All relevant data are within the manuscript and its Supporting Information files.*<br>• If neither of these applies but you are able to provide **details of access elsewhere**, with or without limitations, please do so. For example:<br><br>*Data cannot be shared publicly because of [XXX]. Data are available from the XXX Institutional Data Access / Ethics Committee (contact via XXX) for researchers who meet the criteria for access to confidential data.*<br><br>*The data underlying the results presented in the study are available from (include the name of the third party*	The data underlying the results presented in the study are available on OpenICPSR: https://www.openicpsr.org/openicpsr/project/195667/version/V1/view.

Powered by Editorial Manager® and ProduXion Manager® from Aries Systems Corporation

and contact information or URL).<br>• This text is appropriate if the data are owned by a third party and authors do not have permission to share the data.<br><br>* typeset	
Additional data availability information:	

Manuscript Click here to access/download;Manuscript;Manuscript.docx

Digital health and the promise of equity in maternity care: A mixed methods multi-country assessment on the use of information and communication technologies in healthcare facilities in Latin America and the Caribbean

Short Title: Digital health in maternity care in Latin America and the Caribbean

Ariadna Capasso[1,2*], Mercedes Colomar[3], Dora Ramírez[4], Suzanne Serruya[3], Bremen de Mucio[3]

[1] Health Resources in Action, Boston, MA, USA

[2] New York University, New York, NY, USA

[3] Pan American Health Organization/World Health Organization, Montevideo Uruguay

[4] Independent Consultant, Asunción, Paraguay

* Corresponding author

E-mail: acapasso@hria.org (AC)

// Abstract

Introduction: Timely access to maternity care is critical to saving lives. Digital health may serve to bridge the care chasm and advance health equity. Conducted in the aftermath of the COVID-19 pandemic, this cross-sectional mixed-methods study assessed the use of information and communication technologies (ICTs) in healthcare facilities in nine Latin American and Caribbean countries to understand the landscape of ICT use in maternity care and the barriers and facilitators to its adoption.

Materials and methods: Between April 2021 and September 2022, we disseminated an online survey in English and Spanish among, mainly public, healthcare institutions that provided maternity care in Argentina, Bolivia, Colombia, the Dominican Republic, Ecuador, Guyana, Honduras, Paraguay and Peru. We also interviewed 27 administrators and providers in ministries of health and healthcare institutions.

Results: Most of the 1877 institutions that answered the survey reported using ICTs in maternity care (N=1536, 82%), ranging from 96% in Peru to 64% in the Dominican Republic. Of institutions that used ICTs, 59% reported using them more than before or for the first time since the pandemic began. ICTs were most commonly used to provide family planning (64%) and breastfeeding (58%) counseling, mainly by phone (82%). At the facility level, availability of equipment and internet coverage, coupled with skilled human resources, were the main factors associated with ICT use. At country level, government-led initiatives to develop digital health platforms, alongside national investments in the digital infrastructure, were the determining factors in the adoption of ICTs in healthcare provision.

Conclusion: Digital health for maternity care provision relied on commonly available technology and did not necessitate highly sophisticated systems, making it a sustainable and replicable strategy. However, disparities in access to digital health remain and many facilities in rural and remote areas lacked connectivity. Use of ICTs in maternity care depended on countries' long-term commitments to achieving universal health and digital coverage.

Introduction

Maternal mortality is a grave public health problem in Latin America and the Caribbean, where an estimated 8400 women died in 2020 from causes related to pregnancy and childbirth [1]. It is the only region in the world that has shown no significant improvements in maternal mortality reduction since 2000 [1]. Further, the slow progress made towards reducing maternal deaths over the last two decades was undone by the COVID-19: the maternal mortality ratio increased from 77 deaths per 100,000 live births in 2019 to an estimated 88 deaths per 100,000 live births in 2020 [1].

Deep-seated structural inequalities contribute to disparities in maternal deaths within and between countries [1, 2]. Women of low socioeconomic status, living in rural and remote areas, and of indigenous and African descent, bear the burden of maternal mortality [2]. The maternal mortality ratio of indigenous and afrodescendent women was 1.5 to 3 times higher than that of women of other race/ethnicities every year in Brazil, Guatemala, and Paraguay between 2015 and 2021 [2]. In Paraguay, the maternal mortality ratio among women who did not attend school was 5 times higher than among those with a graduate degree in 2020 [2]. A study examining deaths related to pregnancy and childbirth associated with COVID-19 found that 92% of deaths were among non-White women and 33% among those who had not finished secondary school [3]. The tragedy is that most maternal deaths are preventable with universal access to sexual and reproductive care [4].

Digital health can be a powerful tool to improve population health in resource-constrained settings. In 2005, the World Health Organization (WHO) member states embraced the adoption

of digital health as a strategy to bridge the healthcare access gap and attain universal health coverage [5]. WHO defines digital health as “the cost-effective and safe use of information and communication technologies in support of health and health-related areas,” stating that strengthening health systems through digital health “reinforces fundamental human rights by improving equity, solidarity, quality of life, and quality of care” [6]. In 2011, building on this commitment and under the umbrella of the Pan American Health Organization (PAHO), the countries of Latin America and the Caribbean adopted a plan of action to accelerate progress towards attaining universal health coverage and reducing inequities in access to care through the integration of information and communication technologies (ICTs) in healthcare systems and services [7].

Digital health strategies have two components: the technology used and the main purpose for its use. With this in mind, digital health encompasses a wide variety of strategies that incorporate ICTs to provide and obtain health-related information and advice when distance is a factor, including the remote delivery of healthcare services by providers to patients, virtual consultations among healthcare providers, access to health information by patients (e.g., a health application, an online platform, or a phone line), storage and management of health data (e.g., electronic medical records), telemonitoring through devices that track patient health information at a distance, and management of scheduling and reminders [8]. Digital health interventions in maternity care include virtual routine antenatal and postpartum care visits to provide health counseling, identify warning signs, and answer patient questions; wearables and applications for telemonitoring of pregnant patients with existing conditions, such as gestational diabetes and hypertension; breastfeeding, sexual and reproductive health, and mental health counseling by

phone or video call; using SMS and/or e-mails to send appointment reminders and health information to pregnant and postpartum women; remote consultations between primary care and specialty providers to resolve complex cases; and maternal health data management and surveillance through platforms such as PAHO's Perinatal Information System, among others.

The last PAHO/WHO digital health survey, conducted in 2016, revealed that countries in Latin America and the Caribbean had made significant progress in the area. For example, 61% of countries had a national digital health policy; 90% used teleradiology to store and share radiological images for diagnosis or consultation; 58% used telemonitoring to track patients' vitals at home, particularly for patients with chronic conditions; and 100% employed social media for health education [9]. However, the survey, completed by health ministries, did not inquire about the use of ICTs in maternity care, specifically, nor on the specificities of types of technologies used and services provided with the assistance of ICTs. Further, this data are likely outdated given the rapid penetration of digital technologies in all sectors, particularly since the COVID-19 pandemic [10]. For example, a global systematic review found significant decreases in in-person and increases in virtual antenatal care visits during the COVID-19 pandemic [11]. A 2020 global survey of healthcare providers found that 24% of those in middle income countries used some ICT in maternity care during the COVID-19 pandemic [12].

The evidence on digital health in maternity care is mixed. A review of interventions in high-income countries found that digital health interventions that supplemented in-person mental health and diabetes care during pregnancy with remote contacts resulted in similar or improved clinical outcomes [13]. Further, a reduced-visit antenatal care schedule using telehealth to

replace routine in-person visits in the U.S. obtained similar clinical outcomes and higher patient satisfaction than usual care [14]. A systematic review of ICT use in sexual and reproductive health services in Latin America and the Caribbean found that most interventions entailed the use of SMS for reminders or social media or web-based platforms for education, but fewer involved direct service provision [17]. A caveat of this study is that, since the majority of interventions carried out in health facilities across Latin America and the Caribbean are not documented in the literature, a desk review provides only a partial picture of the reality on the ground. The following are two examples of evaluated digital health interventions in the region. A hybrid antenatal care model for women with high-risk pregnancies in Peru appeared feasible, but no outcomes were reported [15]. In Colombia, an intervention to strengthen the capacity of rural hospitals to respond to obstetric emergencies entailing telehealth and distance learning resulted in a 29% reduction in perinatal deaths [16].

Given the rapid growth of digital health in the aftermath of COVID-19 and the limited evidence on the extent of ICT integration in maternity care, this study aimed to:

1) Describe the use of ICTs in maternity care in a sample of health facilities in 9 countries of Latin America and the Caribbean,
2) Understand changes in the use of ICTs as a result of the COVID-19 pandemic, and
3) Identify the main factors associated with ICT use in maternity care.

Materials and methods

This is a cross-sectional mixed methods study of health facilities providing maternity care in 9 Latin American and Caribbean countries that participated in a PAHO-led program to improve

primary care access: Argentina, Bolivia, Colombia, the Dominican Republic, Ecuador, Guyana, Honduras, Paraguay and Peru. Quantitative data was collected with an online survey questionnaire and qualitative data via key informant interviews, as detailed below. Data for 8 countries were collected between April and September 2021, and for Colombia between July and September 2022. The New York University Institutional Review Board (IRB) did not consider the present study human subjects research because the information collected was not about individuals but about methods, policies, procedures, and organizations; thus, the study did not require IRB submission.

Survey sampling

The survey sampling frame varied by country and was based on what was deemed feasible by national partners (See S1 Table. Sampling frame by country). Briefly, the Dominican Republic, Guyana, Honduras and Peru considered a national sample of all facilities; Argentina and Bolivia considered a national sample of maternity hospitals and facilities associated with the Department of Telemedicine of the Ministry of Health, respectively; and Colombia, Ecuador, and Paraguay focused on priority regions. With the help of national partners, including ministries of health and PAHO country offices, an invitation to complete the survey with a link to an online questionnaire was distributed via e-mail and WhatsApp to maternal health unit directors or telehealth focal points. Instructions asked the potential respondents to complete one survey per facility and to obtain information from other departments, as needed. When the contact information was available, telephone follow-up was conducted a week after the initial invitation was sent. Respondents provided informed consent online by checking two boxes affirming that they understood the terms of the survey and that their participation was voluntary.

Survey Questionnaire

A survey questionnaire was developed in Spanish by an international team of public health practitioners, researchers, and providers with expertise in maternal health and digital health. The questionnaire was informed by PAHO's conceptual framework for telemedicine implementation [8] and guided by the global eHealth survey [9]. The questions were organized under the following themes: 1) general information about the institution and the population served; 2) whether ICTs were used, types of ICTs used, if any, and services provided using ICTs; and 3) enablers of ICT use. To gather data on point 3, respondents were offered a list of five social and nine institutional factors commonly associated with ICT use and asked to rate the extent to which each facilitated their adoption. These factors included budget appropriations, and support to providers, such as training and reimbursement for expenditures incurred when using personal devices. ICT use was assessed by the question "Does your facility use any information and communication technology (ICT) tools for maternal health care (e.g., phone calls, video calls, telemonitoring, text messaging, etc.)?" (Answer options: Yes, the same way as we did before the onset of the pandemic; Yes, more than before the pandemic; Yes, we started using ICT tools when the pandemic was already under way; and No). The variable was dichotomized (Yes/No). The questionnaire was piloted with maternity care providers and administrators in each country to assess face validity and tailor the wording of questions and response options to each national context. The questionnaire was then translated into English and programmed in both languages on Qualtrics (Provo, UT, USA) (See S2 Text. Online survey questionnaire).

Quantitative Analysis Plan

Quantitative analysis involved producing descriptive statistics (frequencies and percentages) using Stata version 17.0 (StataCorp, College Station, TX, USA). Descriptive statistics revealed the over-representation of facilities from Peru and Ecuador. A sensitivity analysis was conducted, showing that in Ecuador and Peru 70% and 96% of respondents used digital health, respectively, compared to 82% on average across all countries. We did not apply any statistical corrections as sampling strategies varied by country.

Qualitative Data Collection and Analysis

In the 7 countries where we found promising examples of ICT use in maternity care, key informant interviews were conducted with maternal health and telehealth focal points in health ministries, and with maternal health providers, to learn more about these interventions. Consent was obtained orally prior to beginning the interview. Overall, 27 in-depth interviews were conducted, lasting from 40 to 60 minutes each. The key informant interview guide consisted of four sections to better understand the digital health strategies implemented and the factors associated with implementation including: 1) details about any digital health intervention used; 2) funding to develop and implement the intervention; 3) enablers of implementation; and 4) lessons learned and future plans for digital health (See S1 Text. Interview guide).

The information gathered from the different data collection methods was complementary: whereas the survey allowed us to collect quantitative data from many health facilities, the qualitative data provided us nuanced information about specific interventions and their implementation context. The qualitative analysis was conducted in NVivo 14 (Lumivero, Denver, CO, USA). We employed a conventional content analysis approach to identify key

contextual factors related to utilization of ICTs [18]. Quantitative and qualitative analyses were done simultaneously in a concurrent design.

Secondary Data

To better understand the setting in which the digital health intervention was implemented, we explored macro factors that could affect the ability of facilities to use ICTs. We examined national level factors related to the digital infrastructure, access, and affordability of ICTs, and a general economic marker. Digital environment data are drawn from the International Telecommunication Union (ITU) Digital Development Dashboard [19]; and the country economies are based on the 2022-2023 World Bank classifications [20]. Lower-middle-income countries are those with a per capita Gross National Income (GNI) of $1,086-$4,255 and upper-middle-income countries are those with a per capita GNI of $4,256-$13,205. For purposes of visualization, the Infrastructure and Access values below the regional average are marked in red on the table. The Broadband Commission for Sustainable Development established as a 2025 target that broadband services be affordable in developing countries, at a cost of less than 2% of the monthly per capita GNI [21]. Affordability values above 2% GNI, the global affordability threshold, are marked in red.

Results

Sample characteristics

After removing 24 duplicates, we analyzed 1877 survey responses. Duplicates occurred when respondents continued working on unsubmitted surveys from a different device. The mean

response rate was 71% (95% Confidence Interval: 44%-97%) (See S2 Table. Response rate by country). Three-quarters (75%) of respondents completed 100% of the survey, and the rest were partial completers.

Primary health care centers (53%) and health posts (23%) comprised the majority of the sample (Table 1). Consistent with this, over three-quarters of institutions represented were primary care level institutions (76%). Peru (23%) and Ecuador (21%) made the largest proportion of the sample, followed by Bolivia (16%).

Table 1. Use of information and communication technologies (ICTs) in the provision of maternity care in selected countries of Latin America and the Caribbean, by type of institution and country (2021-2022), based on survey responses

**Variable**	**Total**	**Used ICTs**
	**n (%)**	
	**1877 (100.0)**	**1536 (81.8)**
**Institution type**		
Referral hospital	160 (8.6)	150 (9.8)
District/regional hospital	180 (9.7)	127 (8.3)
Primary health care center or clinic	990 (53.1)	794 (52.0)
Health post	422 (22.6)	360 (23.6)

Administrative center/health network	113 (6.1)	96 (6.3)
**Level of care**		
Primary	1440 (78.1)	1175 (77.8)
Secondary	230 (12.5)	176 (11.7)
Tertiary	145 (7.9)	135 (8.9)
**Country**		
Argentina	73 (3.9)	56 (3.7)
Bolivia	305 (16.3)	254 (16.5)
Colombia	212 (11.3)	183 (11.9)
Dominican Republic	39 (2.1)	25 (1.6)
Ecuador	402 (21.4)	277 (18.0)
Guyana	72 (3.8)	50 (3.3)
Honduras	83 (4.4)	58 (3.8)
Paraguay	260 (13.9)	219 (14.3)
Peru	431 (23.0)	414 (27.0)

Use of ICTs

Overall, 82% (n = 1536) of health facilities reported using ICTs in maternity care (Fig 1). Peru was the country with the highest proportion of facilities reporting ICT use (96%), followed by Colombia (86%). The Dominican Republic was the country with lowest proportion of facilities reporting ICT use (64%). When examining ICT use by institution type and care level, the highest

proportion of ICT use was indicated by referral hospitals (94%) and tertiary care level centers (93%), respectively. In comparison, 80% of primary health care centers used ICTs.

Fig 1. Proportion of institutions that used ICTs, by country (2021-2022), based on survey responses (N = 1877)

Family planning (64%) and breastfeeding (58%) counseling were the services most commonly provided through the use of ICTs (Table 2). The services less frequently offered via ICT were safe abortion counseling (9%), medical prescription orders (24%), and telemonitoring or long-distance monitoring of high-risk conditions using applications or digital devices (25%). Of note, the proportion of facilities that reported ICT use to provide safe abortion counseling was higher in countries where the procedure is legal in all or certain circumstances, Argentina (36%) and Colombia (30%), and lowest where it is highly criminalized, such as Honduras (0%) (See S3 Table. Use of ICTs for abortion counseling by country). Other services mentioned included teleconsultations between professionals, management of domestic violence situations, management and treatment of sexually transmitted infections, and care of pregnant women with COVID-19 infection, among others.

In terms of the ICTs used in maternity care, an overwhelming majority of facilities used the phone (82%), including applications that enable calling, to communicate with clients (Table 2). To a much lesser extent, other ICTs used included electronic medical records to access client health data (27%), a hotline or dedicated phone number to address pregnancy concerns (27%), and video calls for telemedicine consultations (27%). Provision of telemonitoring devices to

pregnant women and engaging in remote recording and monitoring of pregnancy-related conditions, such as blood sugar levels or blood pressure measurements, by providers were not commonly used. Other responses included using social media (such as Facebook), and national health information platforms (such as WawaRed in Peru [22] or the Federal Platform for Telehealth and Distance Communication in Argentina [23]). Digital health norms and protocols are important to ensure quality of telecare and compliance with ethical standards. Among respondents, 38% of institutions had a telehealth protocol in place and 50% had systems to ensure data security.

Table 2. Maternity care and information and communication technologies (ICTs) in nine Latin American and Caribbean countries (2021-2022), based on survey responses

Variable	Proportion N = 1494 (100.0)*
**Types of services provided using ICTs**	
Safe abortion counseling	134 (9.0)
Orders for medical prescriptions	361 (24.2)
Telemonitoring	374 (25.0)
Gestational disease management	459 (30.7)
Diagnostic antenatal care visit	571 (38.2)
Birth preparation	585 (39.2)
Routine postnatal care visit	628 (42.0)
Routine antenatal care visit	779 (52.3)
Breastfeeding counseling	867 (58.0)

Variable	Proportion<br>N = 1494 (100.0)*
Family planning counseling	962 (64.4)
Other	357 (23.9)
**ICTs used****	
Telemonitoring applications	88 (6.0)
Email address for pregnancy concerns	92 (6.2)
Provision of phones or data to pregnant women	118 (8.0)
Provision of telemonitoring devices to pregnant women	122 (8.3)
Web-based platform for data sharing	185 (12.6)
mHealth (text messages)	217 (14.7)
Video calls for telemedicine consultations	392 (26.6)
Hotline to address pregnancy concerns	397 (26.9)
Electronic medical records	404 (27.4)
Phone calls, including via Apps, for consultations	1205 (81.8)
Other	133 (9.0)
**Protocols and data security*****	
Telehealth protocol in place	378 (27.7)
Data security system	664 (49.6)

Note: *Does not add to 100 because multiple options were possible; ** n = 1,474 due to missing; *** n = 1363 and 1339, respectively, due to missing

Interviewees provided more nuanced descriptions of how ICTs were used to provide services to women during pregnancy and the puerperium. Interviewees described using ICTs for scheduling appointments, to conduct routine antenatal visits, for remote gestational disease management, and for medication prescribing. In Colombia national guidelines during COVID-19 recommended antenatal teleconsultations to conduct anamnesis, maternal risk assessment and monitoring, behavioral counseling, and laboratory analysis requests [24]. Protocols of a tertiary maternity hospital in Peru for remote antenatal care visits recommended similar services, but also included orders for imaging, as relevant to gestational age, as well as COVID-19 tracing [15]. Interviewees in Peru considered having electronic medical records, which providers could access from home, if needed, as a key enabler of teleconsultations.

In addition to direct services, an interviewee from the Ministry of Health of Bolivia, considered ICTs critical to coordinate obstetric emergency responses. He described that staff in referral and emergency coordinating centers in each region used cell phones to coordinate the response to obstetric emergencies and referrals, including ambulance dispatch. Other interviewees from Bolivia and Paraguay mentioned the development of geospatial analysis tools to inform reproductive health supply chain management in remote areas based on the numbers of reproductive-aged and pregnant women. In both cases, the development of these tools was funded by international development and tested as pilot trials. They were complex and resource intensive interventions, and whereas promising in small scale implementation, they proved difficult to sustain, scale-up and integrate into public healthcare systems.

Interviewees from Argentina, Colombia, Peru and Paraguay mentioned the phone as the most common ICT used to reach pregnant women, both for phone calls and text messaging. In Peru

and Paraguay, nurse midwives mentioned forming WhatsApp groups of pregnant women to provide healthy behavior recommendations and to answer questions. A midwife in Paraguay explained, "*each midwife had her own list of patients. [Providers] became more accessible to clients by using WhatsApp to answer concerns and to discuss medical lab results.*" Interviewees described how their facility used social media tools (such as Facebook) for risk communications, to share facility updates, to provide general health recommendations to pregnant women (e.g., vaccination reminders), and for event announcements (e.g., birth preparation workshops). Interviewees in Peru and Argentina described using ICTs, specifically Zoom and YouTube, to offer birth preparation classes. A midwife in Peru recounted opening an educational YouTube channel for pregnant women and recent mothers featuring different specialists, such as pediatricians, endocrinologists, and nutritionists, among others.

Factors associated with ICT use

In terms of strategies implemented by institutions to promote ICT use, overall, 45% of facilities indicated providing some support to staff (Table 3). Of these, 46% trained staff on ICT use, either through in-house programs or by collaborating with health authorities. Only a small percentage of facilities (6%) reimbursed providers for costs incurred when using personal devices or the internet, and 16% furnished providers with cell phones or other mobile devices for telehealth. Roughly 24% of facilities had dedicated IT personnel to address any technological issues encountered while using ICTs, and 60% had a designated telehealth focal point who served as a point of contact for coordinating telehealth use and addressing provider inquiries.

Respondents perceived ICT acceptability by users (26%) as the strongest facilitating social factor. Conversely, cost (37%) and accessibility of ICTs (30%) were the two factors perceived to least facilitate ICT adoption (Fig 2). In terms of institutional factors, 38% of respondents indicated that providers trained in the use of ICTs, and 28% that the availability of adequate equipment and technology, were the main facilitators of ICT adoption. Conversely, 49% of respondents perceived that funding for telehealth was not a facilitating factor. This is not surprising, as only 9% of institutions reported receiving such funding. These digital health initiatives were mostly funded by the public sector (83%). Among these, over half (56%) received the funding during the COVID-19 pandemic.

Fig 2. Social and institutional factors associated with ICT use among institutions that used ICTs, by level of importance.

Table 3. Staff support and funding to support ICT use in maternity care among facilities that used ICTs, based on survey results, 2021-2022 (n = 1391)

**Staff support**	**n (%)**
Facility supported staff	625 (44.9)
Trained providers in ICT use	643 (46.2)
Reimbursed personal internet costs*	39 (6.2)
Distributed cell phones to providers*	103 (16.5)
IT staff assigned to solve problems*	147 (23.5)
Designated telehealth focal point*	378 (60.5)

Institution received funding for telehealth	117 (8.7)
During the pandemic**	65 (55.6)
Public funds**	98 (83.8)

*Percentage calculated over number of institutions that provided support (n = 625);

**Percentage calculated over those who received funding (n = 117)

Qualitative data provided more context to the adoption of ICTs. There were mixed responses related to willingness of providers and patients to use ICTs. Some interviewees mentioned that providers and users were open to using ICTs, particularly cell phones which are ubiquitous in many Latin American contexts, to provide and receive services. An interviewee from Bolivia explained, *“[using ICTs] is acceptable to many women by telephone. And it is accessible. Many users have cell phones with WhatsApp.”* An interviewee from Paraguay noted that communicating with providers by message, calls, and videocalls was acceptable to users but, *“it’s very precarious, there are few phones available, and the lines become very slow.”* However, other interviewees mentioned that providers were reticent to adopt new technologies in their practice. One interviewee described, *“The obstetricians were afraid of giving advice on Zoom. We had to offer a tutorial on the ABC of using Zoom to get them to participate.”* In this sense, several interviewees mentioned that capacity building on the use of ICTs, including on protocols for their use, and having IT staff available to solve technological issues, were necessary to promote their adoption. A barrier mentioned by an interviewee from Bolivia was the lack of computer science engineers working at the Ministry of Health. Some interviewees explained that many low-income households access internet via a cell phone, the only digital device at home. Thus, to receive phone calls or participate in online sessions, women had to

negotiate the use of the cell phone with their partners, and even their children. One interviewee described, "*We had to really work on empowering women to talk to their husbands and convince them that it was important to leave the cell phone with internet at home.*"

According to interviewees, having the digital infrastructure in place was a determinant of ICT use. In Argentina and Peru, for example, the government has invested in building the digital infrastructure for decades. Most public facilities are equipped with internet connection and IT equipment, such as computers. Instead, in Guyana, Honduras, and Paraguay, interviewees mentioned that some regions do not have internet coverage and that many primary care facilities in the public sector lack basic digital infrastructure. A midwife from Paraguay, for example, mentioned staff having to pay out-of-pocket for the internet at work. She explained*, "A group of 22 nurse midwives came together to pay for the monthly internet fee, this allows us to upload productivity, use email, connect via WhatsApp."* One of the biggest challenges reported were the areas with poor internet connection and intermittent connectivity. Interviewees perceived this as a source of disparities, as this issue mainly pertained to rural areas. An interviewee from a rural municipality in Colombia described the challenges of building telehealth services, "*Sometimes there are people who have to climb a hill or climb a tree to be able to catch a signal and sometimes the calls are lost.*" In Paraguay, an interviewee indicated that service providers serving indigenous communities did not use digital health at all given lack of connectivity and IT equipment.

Digital health and the COVID-19 pandemic

During 2020, the COVID-19 pandemic disrupted health services and presented access to care barriers to pregnant and postpartum women. We asked respondents in facilities that used ICTs, the extent to which their use changed as a result of the pandemic. Overall, 42% of respondents said that their ICT use did not change since the COVID-19 pandemic, 39% that they used ICTs more than prior to the pandemic, and 20% that they started using ICTs as a result of the pandemic (Fig 3). Honduras (76%) and the Dominican Republic (68%) had the highest proportion of facilities that did not change their ICT use during the pandemic; facilities in Argentina (63%) and Paraguay (46%) were the most likely to report using ICTs more than before COVID-19; and Peru was the country with more facilities indicating using ICTs for the first time (32%). When asked if they would continue using ICTs in the future, the majority of respondents (84%) indicated they would. The proportion that indicated this was marginally larger in the group who used ICTs more than before the pandemic (90%) (S1 Graph. Future use of ICTs among respondents who used them). One interviewee reflected, *"telehealth will continue in part because it gives us greater flexibility, but we still have to address the problem of connectivity in remote districts."*

Fig 3. ICT use in response to COVID-19

Qualitative analysis revealed multilevel approaches to minimize COVID-19 infection risk among pregnant women and providers while maintaining continuity of care. At the national level, the Ministry of Health and Social Protection of Colombia issued protocols for the provision of maternity and infant care during the emergency. The protocols recommended a hybrid model of antenatal visits that involved a combination of antenatal teleconsultations (25%), home visits (25%), and face-to-face consultations (50%) [24]. According to an interviewee, disparities

emerged in the implementation of this model. Whereas healthcare providers in the private sector had the infrastructure to provide telehealth consultations, they overused them to reduce costs, not properly evaluating obstetric risks. On the other hand, institutions in the public sector faced challenges in implementing the remote visits, even via telephone, due to a lack of connectivity and equipment. The hybrid antenatal care model was discontinued in 2022 with the end of the pandemic.

In Argentina, the ministry of health extended a telehealth platform used for consultations between providers for management and referral of patients with possible COVID-19 infection. The main use of the platform was to evaluate suspected COVID-19 cases and guide the diagnosis and treatment of the patients. Teleconsultations were offered in real-time via videoconferencing. Whereas the platform was not specific to maternity care, pregnant women and recent mothers were considered a priority group.

At the institutional level, a tertiary care maternity hospital in Peru implemented a hybrid antenatal care model for high-risk pregnancies, which entailed alternating consultations and face-to-face visits (50%/50%) [15]. Reflecting on the model’s implementation, an administrator noted the importance of an institutional culture of innovation and prior experience in using ICTs to build upon, *“We were the first institution [in the country] to use telehealth. In 2007, we created the maternal-perinatal telehealth network with the institution's own funds, which facilitated interconsultations between our hospital and other facilities throughout the country... When in-person consultations closed on March 15, 2020, we began testing a telehealth model.”*
Interviewees considered the use of electronic medical records as a facilitating factor, which

enabled providers to access patients' clinical histories from home. Another facilitating factor was having IT personnel on staff to support providers in case of technical difficulties. The hybrid model was widely accepted by users and has expanded since its launch in 2020 to include an app. One limitation of the approach was that it was mainly applied in urban areas of Lima, with good connectivity, in a highly equipped institution, and whose users had access to cell phone or other digital devices.

Another institutional example was a maternity hospital in Argentina which began offering its once in-person birth preparation classes virtually via Zoom, while continuing the normal in-person schedule of antenatal care visits. The modality was highly feasible and acceptable to users. Further, the approach resulted in more family members participating compared to the traditional face-to-face model.

To reduce crowding, facilities in several countries began scheduling antenatal care visits with a set day and time by phone. Prior to the pandemic, providers would only give women a day or week for a return visit. However, interviewees noted that scheduling in advance was difficulted by having physical rather than digital agendas, which made it hard to coordinate scheduling across providers, by women not coming to the scheduled visit, by problems anticipating how many patients would come in person without an appointment, and by problems rescheduling in case of an unexpected occurrence (e.g., if the provider is unavailable). Further, some facilities that instructed women to make appointments by phone, did not have enough human resources to answer the phone. As a result, users spent long hours on the phone trying to schedule or reschedule appointments.

In other cases, ICT use was adopted informally by individuals. In institutions across several countries, nurse midwives began calling their patients to check on them. An interviewee from Argentina explained, *“[Concerned with the lack of controls because of the pandemic...], and having the perinatal clinical history available, midwives began offering teleconsultations to pregnant women.”* These informal approaches, however, were used temporarily and in response to the emergency and were largely discontinued.

National digital and economic setting

To better understand the outer setting in which ICT interventions were implemented in maternity care, this section presents data on economic markers and the national digital infrastructure, including digital coverage, penetration, and affordability. As can be seen in Table 4, all the countries considered in this study are middle-income economies, with Bolivia and Honduras categorized as lower-middle-income and the other countries as upper-middle-income. In terms of digital coverage, most of the population in the countries under study are covered by basic mobile-cellular network. However, gaps remain, particularly in rural areas, with more than 10% of the population not covered by 3G mobile-cellular network in Bolivia, Honduras and Peru. As described above, interviewees in many hard-to-reach areas noted that internet connection was unstable or difficult to access. When examining internet use, we observe large differences by country, with the percentage of the population using the internet ranging from 87% in Argentina to 48% in Honduras. In terms of access to ICTs at home, with the exception of Argentina where the government has actively sought to universalize access by distributing personal computers to

public school children, a majority of households do not have home computers. Internet at home is primarily accessed through mobile phones.

Mobile-broadband subscriptions may include three distinct services: voice, SMS messaging and data. Table 4 presents the monthly cost (expressed as % of per capita GNI) of a basic voice and SMS only (no data) basket and of a high-consumption bundle with an allowance of up to 140 minutes of calls, 70 SMS messages, and 2 GB of data. Many of the countries under study lag behind in affordability, with Colombia and Peru being the only two countries where the cost of subscriptions is below the 2% affordability threshold.

Table 4. National digital and economic setting, 2021

Country	Infrastructure & Access					Affordability		Country Income
	Network Coverage		% Population using the Internet	ICTs at Home				
	Population covered by mobile network	Population covered by 3G mobile network		Internet access at home	Computer at home	Mobile high consumption basket as % of GNI	Mobile voice and SMS (no data) basket as % of GNI	
Argentina	98%	98%	87%	90%	64%	4.0%	2.1%	Upper-middle
Bolivia	100%	88%	66%	57%	28%	7.9%	3.6%	Lower-middle
Colombia	100%	100%	73%	61%	38%	1.9%	1.1%	Upper-middle
Dominican Republic	100%	99%	85%	46%	43%	4.4%	1.6%	Upper-middle
Ecuador	96%	95%	70%	60%	40%	3.2%	2.8%	Upper-middle
Guyana	97%	93%	85%		41%	5.7%	2.8%	Upper-middle
Honduras	89%	82%	48%		16%	10.5%	7.2%	Lower-middle
Paraguay	99%	95%	77%	45%	28%	3.0%	3.0%	Upper-middle
Peru	89%	87%	71%	49%	34%	1.7%	1.7%	Upper-middle
Regional	96%	92%	81%	75%	60%	3.0%	2.0%	

Source of digital environment data: International Telecommunication Union (ITU); Country income based on the World Bank classification. For Infrastructure and Access, the values below the regional average are marked in red; for Affordability values above 2%, the global affordability threshold, are marked in red.

Discussion

This mixed methods study was conducted in nine middle-income Latin American and Caribbean countries to understand how, primarily public sector, facilities used ICTs to provide maternity care. Because it was conducted following the COVID-19 pandemic, we were able to examine the extent to which digital health interventions were implemented in response to the sanitary crisis. We found that ICTs were used for various services along the continuum of antenatal to postnatal care and differed across countries and contexts. Overall, 4 out of 5 institutions reported incorporating ICTs in some way in maternity care. The widespread use of digital health is not surprising given the region's commitment to advancing universal health coverage through technology [25]. Of note, we also found that a digital divide remains, with some populations still unable to benefit from technological developments.

The most frequent uses of ICTs were to provide family planning and breastfeeding counseling over the phone, a largely available technology in the region. These findings are in line with those of a global survey of maternity health professionals, which found that among providers using ICTs, common uses included routine antenatal care (65%), child preparation classes (59%), breastfeeding counseling (50%), and family planning counseling (40%) [12].

Consistent with the literature [26, 27], COVID-19 seems to have accelerated the use of digital health. We found that one-fifth of facilities that used ICTs did so for the first time during the pandemic. Factors associated with the use and adoption of ICTs in maternity care can be classified into individual or team, institutional, and external-level factors, as described below.

Individual and health team-level factors

We found a wide range of attitudes related to ICT use among providers, from reticence to change to willingness to adopt new technologies and modify existing protocols. The barriers to access created by COVID-19 acted as an incentive for individual providers and health teams to embrace change. In fact, we documented several instances in which maternity care providers initiated the use of ICTs to reach pregnant women during COVID-19. Their initiative, motivation, and creativity were critical to the use of ICTs. Often, the reticence to ICT use was related to low perceived self-efficacy and lack of skills to use new technologies. Indeed, provider training in the use of ICTs was ranked as the leading facilitating factor for their adoption. The adoption of ICTs during the COVID-19 pandemic occurred within a high-stress context, with healthcare providers experiencing burnout, uncertainty, and fear. Learning new processes and acquiring new skills is time-consuming, time which providers did not have during the sanitary emergency.

Institutional-level factors

While the contributions of individual and health teams were important, perhaps the determining institutional factor affecting the use of ICTs was the information technology infrastructure. Different strategies require different structures, but at a basic level, inadequate telecommunication systems, including reliable internet connection and mobile network signal, was a major barrier to the adoption of ICTs. Lack of ICT equipment, including computers or tables, as needed, was another barrier.

We also found that, often, the workflows, processes, and staffing structures were not compatible with the use of ICTs. Using ICTs requires adequate staffing for providers to have capacity to

care for both remote and in person patients, and for support staff, including nursing and administrative staff, to arrange scheduling and pull up patient records. Finally, supervisors should be available to provide guidance and feedback on telehealth provision; and systems must be in place to count telehealth services towards the provider's productivity. In the context of scarce resources, providing basic health services was the priority, with limited opportunities to allocate the human, time and financial resources to fully embrace an innovation.

National-level factors

Ultimately, national-level factors were the most important determinants of ICT use. The adoption of digital health at the facility level depended largely on preexisting country commitment to achieving universal health coverage and building the digital infrastructure, evidenced in nearly universal broadband coverage, existing digital healthcare platforms, and public sector facilities with IT equipment. Facilities in countries with a history of investing in developing the digital infrastructure to ensure access to the entire population and that had begun to build national digital health platforms faced fewer barriers to the adoption of ICTs in maternity care than those without. For example, to carry out teleconsultations between providers in different institutions, interoperable systems to share patient records securely, including laboratory results and imaging, must be in place. Among institutions surveyed, most of the funding to expand ICT use was public and received during the pandemic, highlighting the importance of government investments. Overall, our findings are consistent with prior surveys, that identified the national digital infrastructure and government support for telehealth programs, including public funding, as the top factors determining their use [6].

Another factor impacting the adoption of ICTs was the lack of a policy framework to regulate the use of ICTs, particularly of direct provider-patient care, in many Latin American and Caribbean countries at the beginning of the pandemic [28]. Whereas policy regulations around telehealth lagged behind practice, several governments issued new policies to regulate telehealth during the COVID-19 [29], including mandates to public health insurances to reimburse remote care provision.

Strengths and limitations

This study findings should be considered in the light of some limitations. Despite efforts to obtain a representative sample of facilities in each county, it should be noted that facilities in distant areas and areas with low internet connectivity were harder to reach. This bias is particularly pertinent to the subject of this analysis, as we may have over-represented the use of ICTs in maternal health. Further, response rates varied by country, and were particularly low in the Dominican Republic. To mitigate this bias, national consultants travelled to hard-to-reach areas to administer the survey in person and/or called potential respondents to administer the survey verbally. Through our extensive outreach, we were able to obtain a large number of responses, making our findings robust. Because we limited the sample to facilities that provided antenatal care, a majority of institutions represented were primary care institutions. A sample with a larger representation of tertiary care facilities may have yielded different findings, such as more frequent use of complex technologies to provide care. The survey was validated with healthcare providers in each country. However, there may have been differences among respondents in the interpretation of the survey questions, thus introducing measurement errors. The mixed methods study focused on the experiences of health providers and administrators

primarily in public sector facilities that serve the highest-need populations. Future research could focus on user experiences, such as the satisfaction, acceptability and cultural appropriateness of using ICTs to receive maternity care. Another limitation is the cross-sectional design, which captured the experiences of facilities at a specific moment in time. The responses illustrated how ICTs were used at the time of the data collection and the changes in their use during the pandemic. However, it was not clear how widely these had been used prior to the pandemic or the extent to which they will continue to be used after the resolution of the sanitary crisis. Future longitudinal research would provide valuable insights into the continuity and evolution of ICT use in maternity care beyond the immediate context of the pandemic. Such research could assess the long-term benefits and challenges associated with telehealth, shedding light on its effectiveness, sustainability, and impact. Despite these limitations, this study is the first to collect data on ICT use in maternity care from institutions in Latin America and the Caribbean at such a large scale. Data from this study contribute to our understanding of the prevalence of ICT use, the technologies used, the services provided, and the factors affecting their use and can inform the design of interventions to improve maternity care through the integration of digital health solutions.

Implications for practice

The integration of ICTs in maternity care is a promising strategy in Latin America and the Caribbean. Considerations for their adoption include the level of complexity of the intervention and the cost and availability of the technology required to implement it. Other considerations include the facility’s staffing levels, the digital infrastructure, and institutional workflows and protocols, as well as the existing national regulatory framework. Many of the interventions we

found relied on the phone, a widely available technology that many people know how to use. However, implementing any innovation requires substantial planning, coordination, and training. In our study, interviewees reported that apparently simple interventions faced challenges. For example, facilities trying to implement advanced scheduling of antenatal care visits by phone encountered insufficient human resources to schedule and/or reschedule visits, and to coordinate care given no-shows and patients without appointments. The more complex the system or platform, the more resources and time are needed for its development and implementation. Health data sharing, for instance, requires systems that are secure and interoperable across institutions.

For institutions ready to expand the use of telehealth, PAHO has developed a tool for institutions to self-assess their readiness to use ICTs, identify gaps, and prioritize investments in improving data use and data collection [30]. The tool provides guidance on assessing telehealth readiness of the infrastructure, human resources and capacity, and the use health data to improve care quality. Further, WHO has also issued guidelines for facilities seeking to incorporate telehealth for systems strengthening [31].

Conclusions

In this mixed methods study, we found that many facilities throughout the Latin American and Caribbean region incorporated some form of ICTs in maternity care. Respondents considered ICTs as a promising tool to improve care quality and access. During the COVID-19 pandemic, many facilities turned to ICTs to connect with pregnant women, communicate with other providers, and manage patient flow to reduce congestion. Facilities relied on low-cost

technologies that were readily available. However, findings also highlighted that ICTs may exacerbate disparities in maternity care. In particular, primary care facilities in rural areas with deficient digital infrastructure were disproportionately excluded from the benefits of telehealth.

Furthermore, the study findings revealed that while there were examples of bilateral investments to develop complex digital platforms, they were not sustainable in the long run. Instead, systems led and coordinated by the government, as part of concerted national efforts to expand the use of ICTs in healthcare, alongside the development of the national digital infrastructure, were found to be the most sustainable and promising for the future.

Overall, more research is needed to understand which digital strategies will persist post-COVID and their impact on access to care and maternal and perinatal outcomes. Research focused on understanding implementation aspects of digital strategies will be particularly useful in promoting scale-up and increasing uptake. Examining the long-term use and outcomes of these digital strategies will provide valuable insights into their effectiveness and potential for addressing existing health disparities.

Acknowledgments

The authors wish to thank the following individuals for their support in outreach and national data collection: Andrea Mirella Arpita Rojas, Carlos Ayala, María Victoria Bertolino, Alana Browne, Guillermo Carroli, Evelyne Ancion Degraff, Alexia Escóbar Vásquez, Xiomara Fernández, Rosalinda Hernández, Elodia Jara, Haydée Padilla, Margarita Pérez, Bertha Pooley, Kenia Mariel Ramos Aguilar, Nora Redondo, Catherina Rodriguez, Mirian Rojas, Erick

Rousselin, Claudio Sosa, Luis Urbina, Nora Edith Valdivia Soto, Janice Woolford, as well as all the interviewees in ministries of health and health facilities for their time and invaluable contributions.

References

1. World Health Organization. Trends in maternal mortality 2000 to 2020: estimates by WHO, UNICEF, UNFPA, World Bank Group and UNDESA/Population Division. Geneva: World Health Organization; 2023. Available from: https://www.who.int/publications/i/item/97892400687592.
2. Cuevas L. Informe sobre los datos actualizados y análisis de indicadores clave de salud materna y razón de mortalidad materna de países de Latino América y el Caribe, periodo 2015-2021 [Report on updated data and analysis of key maternal health indicators and maternal mortality ratio in Latin American and Caribbean countries, period 2015-2021]. Panama City: Regional Task Force for the Reduction of Maternal Mortality; 2023.
3. Maza-Arnedo F, Paternina-Caicedo A, Sosa CG, de Mucio B, Rojas-Suarez J, Say L, et al. Maternal mortality linked to COVID-19 in Latin America: Results from a multi-country collaborative database of 447 deaths. Lancet Reg Health Am. 2022;12:100269. doi:10.1016/j.lana.2022.100269.
4. Chou D, Daelmans B, Jolivet RR, Kinney M, Say L; Every Newborn Action Plan (ENAP) and Ending Preventable Maternal Mortality (EPMM) working groups. Ending preventable maternal and newborn mortality and stillbirths. BMJ. 2015 Sep 14;351:h4255. doi: 10.1136/bmj.h4255.
5. World Health Organization. WHA58.28 eHealth Ninth plenary meeting, 25 May 2005 – Committee A, seventh report), (2005). Available from: https://apps.who.int/gb/ebwha/pdf_files/WHA58/WHA58_28-en.pdf

6. World Health Organization. Global difusion of eHealth: making universal health coverage achievable. Report of the third global survey on eHealth. Geneva: WHO; 2016. Available from: https://apps.who.int/iris/handle/10665/252529

7. Pan American Health Organization. CD51/13: Strategy and plan of action on eHealth, Washington D.C.: PAHO; 2011. Available from: https://iris.paho.org/handle/10665.2/7663

8. Pan American Health Organization. Framework for the implementation of a telemedicine service. Washington, D.C.: PAHO; 2016. Available from: https://iris.paho.org/handle/10665.2/28414

9. Pan American Health Organization. eHealth in the Region of the Americas: breaking down the barriers to implementation. Results of the World Health Organization's Third Global Survey on eHealth. Washington, D.C.: PAHO; 2016. Available from: https://iris.paho.org/handle/10665.2/31286

10. Jung J, Katz R. Impacto del COVID-19 en la digitalización de América Latina [Impact of COVID-19 on the digitalization of Latin America]. Santiago: Economic Commision for Latin America and the Caribbean; 2023. Available from: https://repositorio.cepal.org/server/api/core/bitstreams/cf05ce4b-b465-4740-86a1-6b707267e99b/content

11. Townsend R, Chmielewska B, Barratt I, Kalafat E, van der Meulen J, Gurol-Urganci I, et al. Global changes in maternity care provision during the COVID-19 pandemic: A systematic review and meta-analysis. eClinicalMedicine. 2021;37:100947. doi: 10.1016/j.eclinm.2021.100947

12. Galle A, Semaan A, Huysmans E, Audet C, Asefa A, Delvaux T, et al. A double-edged sword-telemedicine for maternal care during COVID-19: findings from a global mixed-methods study of healthcare providers. BMJ Glob Health. 2021;6(2). doi: 10.1136/bmjgh-2020-004575

13. Cantor AG, Jungbauer RM, Totten AM, Tilden EL, Holmes R, Ahmed A, et al. Telehealth strategies for the delivery of maternal health care: A rapid review. Ann Intern Med. 2022;175(9):1285-97. doi: 10.7326/M22-0737

14. Peahl AF, Smith RD, Moniz MH. Prenatal care redesign: creating flexible maternity care models through virtual care. Am J Obstet Gynecol. 2020;223(3):389.e1-389.e10. doi: 10.1016/j.ajog.2020.05.029.

15. Meza-Santibañez LH, Novoa R, Torres-Osorio J, Jáuregui-Canchari V, Rodríguez-Hilario N, Guevara-Ríos E, et al. Implementation of a mixed model of in person and virtual prenatal care during the COVID-19 pandemic at the National Maternal Perinatal Institute in Lima, Peru. Revista Peruana de Ginecología y Obstetricia. 2021;67(2). doi: 10.31403/rpgo.v67i2313

16. Escobar MF, Echavarria MP, Vasquez H, Nasner D, Ramos I, Hincapié MA, et al. Experience of a telehealth and education program with maternal and perinatal outcomes in a low-resource region in Colombia. BMC Pregnancy Childbirth. 2022;22(1):604. doi: 10.1186/s12884-022-04935-1

17. Pan American Health Organization. Technical study on the availability of digital solutions to improve maternal and sexual and reproductive health. Washington, D.C.: PAHO; 2021.

18. Hsieh HF, Shannon SE. Three approaches to qualitative content analysis. Qual Health Res. 2005;15(9):1277-88. doi: 10.1177/1049732305276687

19. International Telecommunication Union (ITU). Digital Development Dashboard. Geneva: ITU; 2023. Available from: https://www.itu.int/en/ITU-D/Statistics/Dashboards/Pages/Digital-Development.aspx.

20. World Bank. World Bank country classifications by income level, 2022-2023. Geneva: World Bank; 2022. Available from: https://blogs.worldbank.org/opendata/new-world-bank-country-classifications-income-level-2022-2023.

21. Broadband Commission for Sustainable Development. 2025 Targets: Connecting the Other Half. Available from: https://www.broadbandcommission.org/broadband-targets/.

22. Pérez-Lu JE, Iguiñiz Romero R, Bayer AM, García PJ. Reduciendo las inequidades en salud y mejorando la salud materna mediante la mejora de los sistemas de información en salud: Wawared Perú [Wawared Peru: reducing health inequities and improving maternal health by improving information systems in health]. Rev Peru Med Exp Salud Publica. 2015;32(2):373-377.

23. Argentina. Ministerio de Salud. Qué es la Telesalud. [What is telehealth?] Argentina.gob.ar, 2020. Available from: https://www.argentina.gob.ar/salud/telesalud/definicion.

24. Colombia. Ministerio de Salud y Protección Social (MSPS). Lineamientos provisionales para la atención en salud de las gestantes, recién nacidos y para la lactancia materna, en el contexto de la pandemia de covid-19 en Colombia [Interim guidelines for health care for pregnant women, newborns and breastfeeding, in the context of the Covid-19 pandemic in Colombia]. Bogota; MSPS; junio 2020. Available from:

https://www.minsalud.gov.co/Ministerio/Institucional/Procesos%20y%20procedimientos/GIPS14.pdf

25. World Health Organization, World Bank. Tracking universal health coverage: 2017 global monitoring report. WHO and World Bank; 2017. Available from: https://apps.who.int/iris/handle/10665/310924

26. Madubuonwu J, Mehta P. How telehealth can be used to improve maternal and child health outcomes: a population approach. Clin Obstet Gynecol. 2021;64(2):398-406. doi:10.1097/GRF.0000000000000061027.

27. Bouabida K, Lebouché B, Pomey MP. Telehealth and COVID-19 pandemic: an overview of the telehealth use, advantages, challenges, and opportunities during COVID-19 pandemic. Healthcare (Basel). 2022;10(11):2293. doi:10.3390/healthcare1011229328.

28. Aizenberg M. Regulatory framework for telemedicine: current status and next steps. Washington, D.C.: Inter-American Development Bank; 2022. Available from: https://publications.iadb.org/publications/english/viewer/Regulatory-Framework-for-Telemedicine-Current-status-and-Next-Steps.pdf

29. Camacho-Leon G, Faytong-Haro M, Carrera K, Molero M, Melean F, Reyes Y, et al. A narrative review of telemedicine in Latin America during the COVID-19 pandemic. Healthcare (Basel). 2022;10(8). doi: 10.3390/healthcare10081361

30. Pan American Health Organization. Rapid assessment tool for critical data gathering. Washington, D.C.: PAHO; 2021. Available from: https://iris.paho.org/handle/10665.2/53614

31. World Health Organization. WHO guideline recommendations on digital interventions for health system strengthening. Geneva: WHO; 2019. Available from: https://iris.who.int/bitstream/handle/10665/311941/9789241550505-eng.pdf?ua=1

Supporting information

S1 Table. Sampling frame by country.

S2 Table. Response rate by country.

S3 Table. Use of ICTs for abortion counseling by country.

S1 Text. Interview guide.

S2 Text. Online survey questionnaire.

S1 Graph. Future use of ICTs among respondents who used them (n = 1459).

Figure 2

Click here to access/download;Figure;Fig2.tif

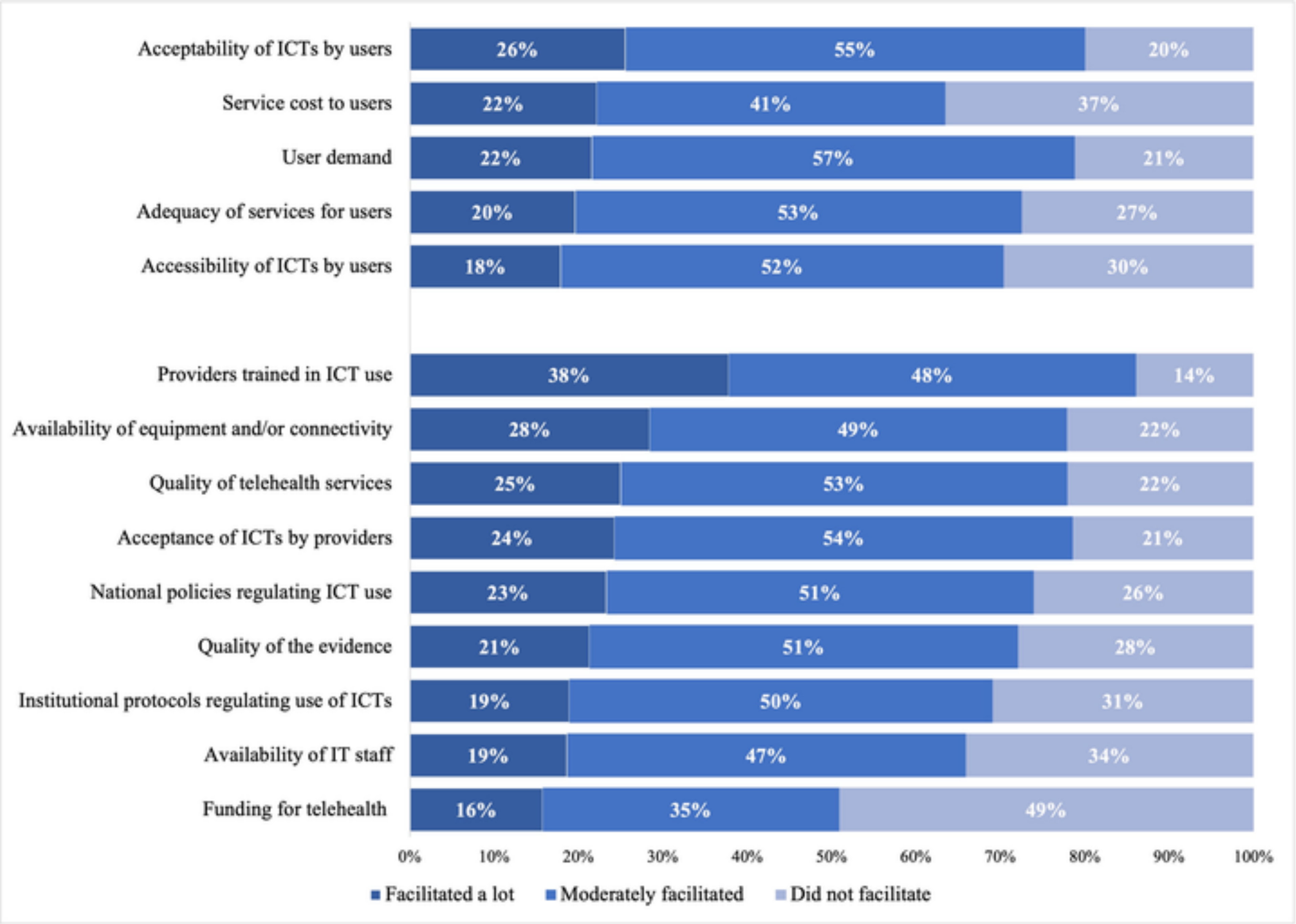

Figure 3

Click here to access/download;Figure;Fig3.tif

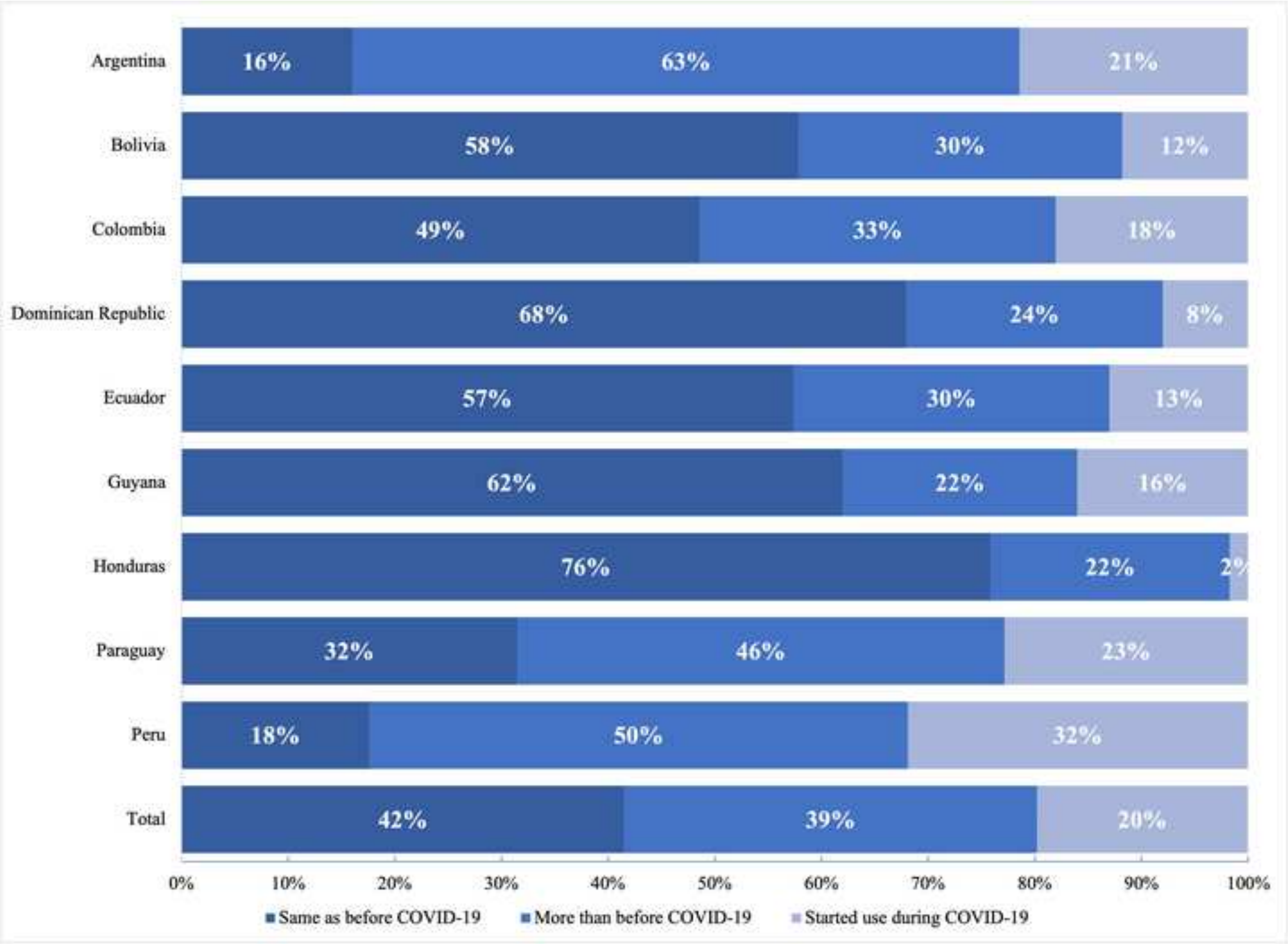

Figure 1

Click here to access/download;Figure;Fig1.tif

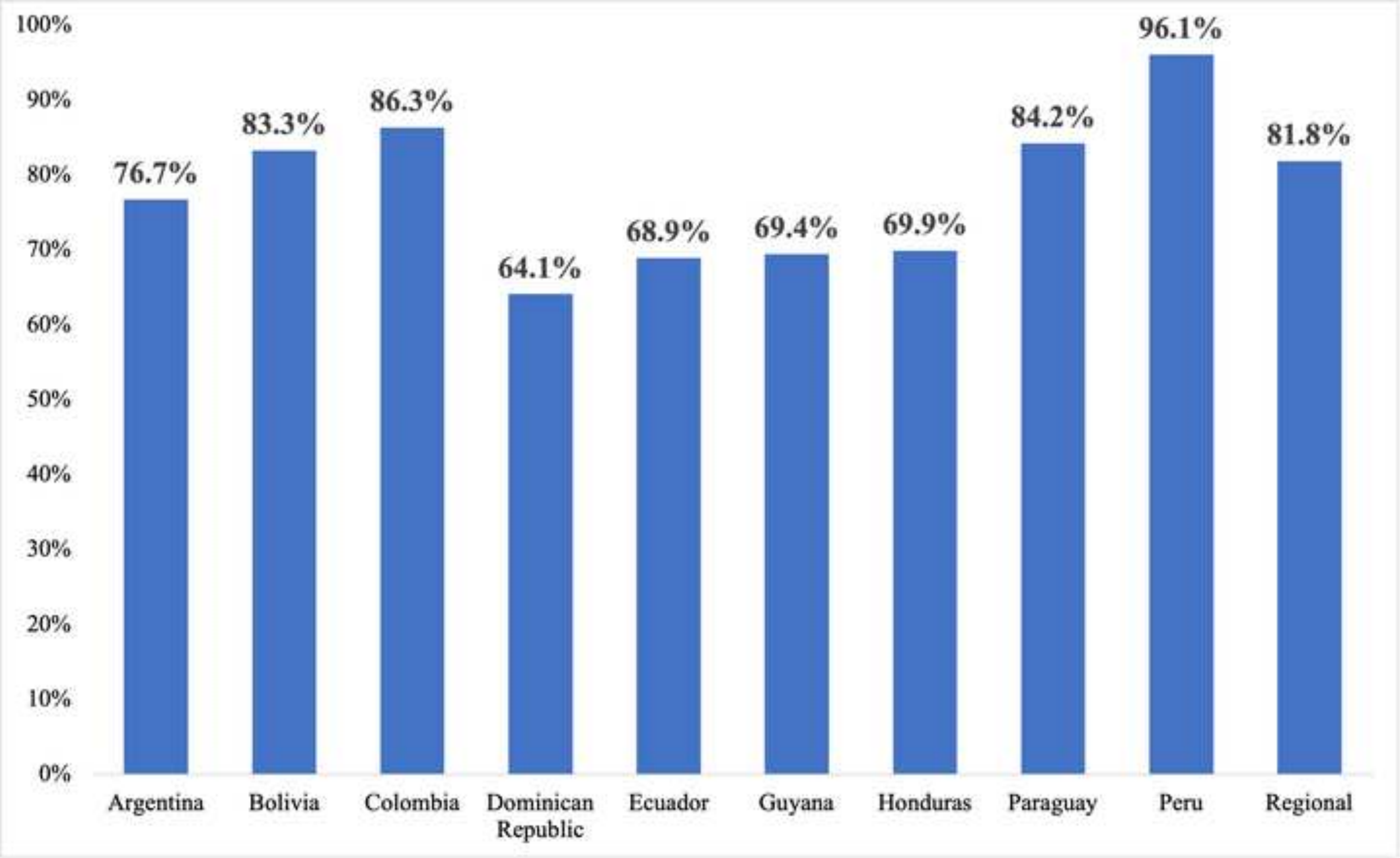

Click here to access/download
Supporting Information - Compressed/ZIP File Archive
Supporting information.zip

Revised Manuscript with Track Changes

Digital health and the promise of equity in maternity care: A mixed methods multi-country assessment on the use of information and communication technologies in healthcare facilities in Latin America and the Caribbean

Short Title: Digital health in maternity care in Latin America and the Caribbean

Ariadna Capasso[1,2*], Mercedes Colomar[3], Dora Ramírez[4], Suzanne Serruya[3], Bremen de Mucio[3]

[1] Health Resources in Action, Boston, MA, USA

[2] New York University, New York, NY, USA

[3] Pan American Health Organization/World Health Organization, Montevideo Uruguay

[4] Independent Consultant, Asunción, Paraguay

* Corresponding author

~~E-mail: acapasso@hria.org~~

E-mail: acapasso@hria.org (AC)

24

2

Abstract

Introduction: Timely access to maternity care is critical to saving lives. Digital health may serve to bridge the care chasm and advance health equity. Conducted in the aftermath of the COVID-19 pandemic, this cross-sectional mixed-methods study assessed the use of information and communication technologies (ICTs) in healthcare facilities in nine Latin American and Caribbean countries to understand the landscape of ICT use in maternity care and the barriers and facilitators to its adoption.

~~Methods~~Materials and methods: Between April 2021 and September ~~of~~ 2022, we disseminated an online survey in English and Spanish among, mainly public, healthcare institutions that ~~provide~~provided maternity care in Argentina, Bolivia, Colombia, the Dominican Republic, Ecuador, Guyana, Honduras, Paraguay and Peru. We also interviewed 27 administrators and providers in ministries of health and healthcare institutions.

Results: Most of the 1877 institutions that answered the survey reported using ICTs in maternity care (N=1536, 82%), ranging from 96% in Peru to 64% in the Dominican Republic. Of institutions that used ICTs, 59% reported using them more than before or for the first time since the pandemic began. ICTs were most commonly used to provide family planning (64%) and breastfeeding (58%) counseling ~~(58%),~~, mainly by phone (82%). ~~Skilled human resources and~~ At the facility level, availability of equipment ~~(e.g., fast~~and internet~~)~~ coverage, coupled with skilled human resources, were the main factors associated with ICT use. ~~Many facilities~~At country level, government-led initiatives to develop digital health platforms, alongside national investments in

Formatted: Font: 18 pt

Formatted: Don't adjust space between Latin and Asian text, Don't adjust space between Asian text and numbers

Formatted: Default Paragraph Font, Font color: Auto

~~rural and remote areas lacked connectivity.~~the digital infrastructure, were the determining factors in the adoption of ICTs in healthcare provision.

Formatted: Default Paragraph Font, Font color: Auto

Conclusion: Digital health for ~~provision~~ maternity care provision relied on commonly available technology and did not necessitate highly sophisticated systems, making it a sustainable and replicable ~~in other settings.~~strategy. However, disparities in access to digital health remain~~.~~ and many facilities in rural and remote areas lacked connectivity. Use of ICTs in maternity care depended on ~~country's~~countries' long-term commitments to achieving universal health ~~coverage and to building the~~and digital ~~infrastructure~~coverage.

Formatted: Font: Not Bold

Formatted: Line spacing: single

Introduction

Formatted: Font: 18 pt

Maternal mortality is a grave public health problem in Latin America and the Caribbean, where an estimated 8400 women died in 2020 from causes related to pregnancy and childbirth [1]. It is the only region in the world that has shown no significant improvements in ~~reducing~~ maternal ~~deaths~~mortality reduction since 2000 [1]. Further, the slow progress made towards ~~eradicating~~reducing maternal deaths over the last two decades was ~~erased during~~undone by the COVID-19: the maternal mortality ratio increased from 77 deaths per 100,000 live births in 2019 to an estimated 88 deaths per 100,000 live births in 2020 [1].

Deep-seated structural inequalities contribute to ~~large~~ disparities in maternal deaths within and between countries [1, 2]. Women of low socioeconomic status, living in rural and remote areas, and of indigenous and African descent, bear the burden of maternal mortality [2]. The maternal mortality ratio of indigenous and afrodescendent women was 1.5 to 3 times higher than that of women of other race/ethnicities every year in Brazil, Guatemala, and Paraguay between 2015 and 2021 [2]. In Paraguay, the maternal mortality ratio among women who did not attend school was 5 times higher than among those ~~who had~~with a graduate degree in 2020 [2]. A study examining deaths related to pregnancy and childbirth associated with COVID-19 found that 92% of deaths were among non-White women and 33% among those who had not finished secondary school [3]. The tragedy ~~of~~is that most maternal deaths ~~is that most~~ are preventable with ~~access to~~ universal access to sexual and reproductive care [4].

Digital health can be a powerful tool to improve population health in resource-constrained settings. In 2005, the World Health Organization (WHO) member states embraced the adoption of digital health as a strategy to bridge the healthcare access gap and attain universal health coverage [5]. WHO defines digital health as “the cost-effective and safe use of information and communication technologies in support of health and health-related areas,” stating that strengthening health systems through digital health “reinforces fundamental human rights by improving equity, solidarity, quality of life, and quality of care” [6]. In 2011, ~~as a follow-up to~~building on this commitment and under the umbrella of the Pan American Health Organization (PAHO), the countries of Latin America and the Caribbean adopted a plan of action to accelerate progress towards attaining universal health coverage and reducing inequities in access to care through the integration of information and communication technologies (ICTs) in healthcare systems and services [7].

Digital health strategies have two components: the technology used and the main purpose for its use. With this in mind, digital health encompasses a wide variety of strategies that incorporate ICTs to provide and obtain health-related information and advice when distance is a factor, including the remote delivery of healthcare services by providers to patients, virtual consultations among healthcare providers, access to health information by patients (e.g., a health application, an online platform, or a phone line), storage and management of health data (e.g., electronic medical records), telemonitoring through devices that track patient health information at a distance, and management of scheduling and reminders [8]. Digital health interventions in maternity care include virtual routine antenatal and postpartum care visits to provide health counseling, identify warning signs, and answer patient questions; wearables and applications for

telemonitoring of pregnant patients with existing conditions, such as gestational diabetes and hypertension; breastfeeding, sexual and reproductive health, and mental health counseling by phone or video call; using SMS and/or e-mails to send appointment reminders and health information to pregnant and postpartum women; remote consultations between primary care and specialty providers to resolve complex cases; and maternal health data management and surveillance through platforms such as PAHO's Perinatal Information System, among others.

The last PAHO/WHO digital health survey~~ of~~, conducted in 2016, revealed that countries in Latin America and the Caribbean had made significant progress in the area. For example, 61% of countries had a national digital health policy; 90% used teleradiology to store and share radiological images for diagnosis or consultation; 58% used telemonitoring to track patients' vitals at home, particularly for patients with chronic conditions; and 100% employed social media for health education [9]. However, the survey, completed by health ministries, did not inquire about the use of ICTs in maternity care, specifically, nor on the specificities of types of technologies used and services provided with the assistance of ICTs. ~~A recent~~Further, this data are likely outdated given the rapid penetration of digital technologies in all sectors, particularly since the COVID-19 pandemic [10]. For example, a global systematic review found significant decreases in in-person and increases in virtual antenatal care visits during the COVID-19 pandemic [11]. A 2020 global survey of healthcare providers found that 24% of those in middle income countries used some ICT in maternity care ~~[10].~~during the COVID-19 pandemic [12].

The evidence on digital health in maternity care is mixed. ~~Systematic reviews~~A review of interventions in high-income countries found that digital health interventions that supplemented

in-person mental health and diabetes care during pregnancy with remote contacts resulted in similar or improved clinical outcomes [11].[13]. Further, a reduced-visit antenatal care schedule using telehealth to replace routine in-person visits resulted inin the U.S. obtained similar clinical outcomes and higher patient satisfaction versusthan usual care [11-13]. However, these reviews included only examples from high-income countries.[14]. A systematic review of ICT use in sexual and reproductive health services in Latin America and the Caribbean found that most interventions entailed the use of SMS for reminders or social media or web-based platforms for education, but fewer involved direct service provision [14]. A hybrid antenatal care model in Peru for high-risk pregnancies also[17]. A caveat of this study is that, since the majority of interventions carried out in health facilities across Latin America and the Caribbean are not documented in the literature, a desk review provides only a partial picture of the reality on the ground. The following are two examples of evaluated digital health interventions in the region. A hybrid antenatal care model for women with high-risk pregnancies in Peru appeared feasible, but no outcomes were reported [15]. In Colombia, an intervention to strengthen the capacity of rural hospitals to respond to obstetric emergencies entailing telehealth and distance learning resulted in a 29% reduction in perinatal deaths [16].

Formatted: Font color: Text 1

The COVID-19 pandemic posed important barriers to accessing maternity care and gave a renewed impetus to the adoption of digital health. A global systematic review found significant decreases in in-person antenatal care visits and increases in virtual antenatal care during the COVID-19 pandemic [16]. To inform the design of future strategies that incorporate ICTs in maternity health, the current mixed methods study aimed to:

Given the rapid growth of digital health in the aftermath of COVID-19 and the limited evidence on the extent of ICT integration in maternity care, this study aimed to:

1) Describe the use of ICTs in maternity care in a sample of ~~healthcare~~health facilities in 9 countries of Latin America and the Caribbean,
2) Understand ~~how~~changes in the use of ICTs as a result of the COVID-19 pandemic~~ shaped the implementation of digital health strategies~~, and
3) Identify the main factors associated with ~~the~~ICT use~~ of digital health~~ in maternity care.

Materials and ~~Methods~~methods

~~**Study design, population and sampling**~~

This is a cross-sectional mixed methods study of health facilities providing maternity care in 9 Latin American and Caribbean countries that participated in a PAHO-led program to improve primary care access: Argentina, Bolivia, Colombia, the Dominican Republic, Ecuador, Guyana, Honduras, Paraguay and Peru. Quantitative data was collected with an online survey questionnaire and qualitative data via key informant interviews, as detailed below. Data for 8 countries were collected between April and September 2021, and for Colombia between July and September 2022. The New York University Institutional Review Board (IRB) did not consider the present study human subjects research because the information collected was not about individuals but about methods, policies, procedures, and organizations; thus, the study did not require IRB submission.

Survey sampling

Formatted: Normal

Formatted: Font: 18 pt

Formatted: Font: 18 pt

The survey sampling frame varied by country and was based on what was deemed feasible following discussions with by national partners (See Annex A for detailsS1 Table. Sampling frame by country). Briefly, the Dominican Republic, Guyana, Honduras and Peru considered a national sample of all facilities; Argentina and Bolivia considered a national sample of maternity hospitalhospitals and facilities associated with the Department of Telemedicine of the Ministry of Health, respectively; and Colombia, Ecuador, and Paraguay focused on priority regions. With the help of national partners, including ministries of health and PAHO country offices, an invitation to complete the survey with a link to an online questionnaire was distributed via e-mail and WhatsApp to maternal health unit directors or telehealth focal points. Instructions asked the potential respondents to complete one survey per facility and to obtain information from other departments, as needed. When the contact information was available, telephone follow-up was conducted a week after the initial invitation was sent. Respondents provided informed consent online by checking two boxes affirming that they understood the terms of the survey and that their participation was voluntary. In 7 countries, interviews were conducted with maternal health and telehealth focal points in health ministries, and maternal health providers, to gain more insights into contextual factors impeding or supporting ICT use. Consent was obtained orally prior to beginning the interview. Overall, 27 in-depth interviews were conducted, lasting from 40 to 60 minutes each. The key informant interview guide consisted of four sections to better understand if and how digital health strategies had been implemented and the factors associated with implementation including: 1) details about any digital health intervention used; 2) funding to develop and implement the intervention; 3) enablers of implementation; and 4) lessons learned and future plans for digital health (See Annex B. Interview guide). Data for 8 countries were gathered during the first phase of data collection between April and September 2021; whereas

~~data for Colombia were collected during the second phase, between July and September 2022. The New York University Institutional Review Board (IRB) does not consider the present study human subjects research because the information collected is not about individuals but about methods, policies, procedures, and organizations, and does not require application to the IRB.~~

Formatted: Font: Bold

Survey Questionnaire

Formatted: Font: 16 pt

A survey questionnaire was developed in Spanish by an international team of public health practitioners, researchers, and providers with expertise in maternal health and digital health. The questionnaire was informed by PAHO’s conceptual framework for telemedicine implementation [8] and guided by the global eHealth survey [9]. The questions were organized under the following themes: 1) general information about the institution and the population served; 2) whether ICTs were used, types of ICTs used, if any, and services provided using ICTs; and 3) enablers ~~and barriers~~ of ICT use. To gather data on point 3, respondents were offered a list of five social and nine institutional factors commonly associated with ICT use and asked to rate the extent to which each facilitated their adoption. These factors included budget appropriations, and ~~supports~~support to providers, such as training and ~~refund~~reimbursement for expenditures incurred when using personal devices. ~~We also asked how current ICT use compared to prior to the COVID-19~~ICT use was assessed by the question “Does your facility use any information and communication technology (ICT) tools for maternal health care (e.g., phone calls, video calls, telemonitoring, text messaging, etc.)?” (Answer options: Yes, the same way as we did before the onset of the pandemic~~.~~; Yes, more than before the pandemic; Yes, we started using ICT tools when the pandemic was already under way; and No). The variable was dichotomized (Yes/No). The questionnaire was piloted with maternity care providers and administrators in each country

to assess face validity and tailor the wording of questions and response options to each national context. The questionnaire was then translated into English and programmed in both languages on Qualtrics (Provo, UT, USA) (See Annex C on-lineS2 Text. Online survey questionnaire).

Formatted: Font: Not Bold

Quantitative Analysis Plan

Formatted: Font: 16 pt

After removing 24 duplicates, we analyzed 1877 survey responses. The mean response rate was 71% (95% Confidence Interval: 44%-97%) (Annex D). Three-quarters (75%) of respondents completed 100% of the survey, and the rest were partial completers. Quantitative and qualitative analyses were done simultaneously in a concurrent design. Quantitative analysis involved producing descriptive statistics (frequencies and percentages) using Stata version 17.0 (StataCorp, College Station, TX, USA). Descriptive statistics revealed the over-representation of facilities from Peru and Ecuador. A sensitivity analysis was conducted, showing that in Ecuador and Peru 70% and 96% of respondents used digital health, respectively, compared to 82% on average across all countries. We did not apply any statistical corrections as sampling strategies varied by country.

Qualitative Data Collection and Analysis

In the 7 countries where we found promising examples of ICT use in maternity care, key informant interviews were conducted with maternal health and telehealth focal points in health ministries, and with maternal health providers, to learn more about these interventions. Consent was obtained orally prior to beginning the interview. Overall, 27 in-depth interviews were conducted, lasting from 40 to 60 minutes each. The key informant interview guide consisted of four sections to better understand the digital health strategies implemented and the factors

associated with implementation including: 1) details about any digital health intervention used; 2) funding to develop and implement the intervention; 3) enablers of implementation; and 4) lessons learned and future plans for digital health (See S1 Text. Interview guide).

The information gathered from the different data collection methods was complementary: whereas the survey allowed us to collect quantitative data from many health facilities, the qualitative data provided us nuanced information about specific interventions and their implementation context. The qualitative analysis was conducted in NVivo 14 (Lumivero, Denver, CO, USA). We employed a conventional content analysis approach to identify key contextual factors related to utilization of ICTs [17].

[18]. Quantitative and qualitative analyses were done simultaneously in a concurrent design.

Secondary Data

Formatted: Font: 16 pt

To better understand the setting in which the digital health intervention was implemented, we contextualized the findings by exploringexplored macro factors that could affect the ability of facilities to use ICTs. We examined national level factors related to the digital infrastructure, access, and affordability of ICTs, and a general economic marker. Digital environment data are drawn from the International Telecommunication Union (ITU) Digital Development Dashboard [18][19]; and the country economies are based on the 2022-2023 World Bank classifications [19].[20]. Lower-middle-income countries are those with a per capita Gross National Income (GNI) of $1,086-$4,255 and upper-middle-income countries are those with a per capita GNI of $4,256-$13,205. For purposes of visualization, the Infrastructure and Access values below the regional average are marked in red on the table. The Broadband Commission for Sustainable

Development established as a 2025 target that broadband services ~~should be affordable to the population in developing countries, at a cost of less than 2% of the monthly per capita GNI [20].~~be affordable in developing countries, at a cost of less than 2% of the monthly per capita GNI [21]. Affordability values above 2% GNI, the global affordability threshold, are marked in red.

Results

Sample characteristics

After removing 24 duplicates, we analyzed 1877 survey responses. Duplicates occurred when respondents continued working on unsubmitted surveys from a different device. The mean response rate was 71% (95% Confidence Interval: 44%-97%) (See S2 Table. Response rate by country). Three-quarters (75%) of respondents completed 100% of the survey, and the rest were partial completers.

~~Implementation science is a field of research that seeks to bridge the gap between evidence and practice, by systematically studying factors that affect the adoption of evidence-based interventions [21]. To increase the usefulness of the current study's findings to administrators and practitioners wishing to implement digital health interventions, the discussion frames the factors associated with ICT use according to the constructs of the Consolidated Framework for Implementation Research (CFIR). CFIR is a conceptual framework that serves to guide the systematic assessment of multilevel implementation contexts to understand the factors associated with the adoption of evidence-based practices in clinical settings [22]. CFIR defines constructs along the following four domains: 1) Innovation: The program or practice being implemented, e.g., complementing in-person antenatal care visits with remote visits; 2) Inner Setting: The~~

Formatted: Font: 18 pt

Formatted: Font: 16 pt

Formatted: Don't adjust space between Latin and Asian text, Don't adjust space between Asian text and numbers

Formatted: Font color: Custom Color(RGB(19,20,19))

setting in which the innovation is implemented, e.g., a health post, hospital, or health network; 3) Outer Setting: The context in which the inner setting exists, and which affects how the intervention is implemented, e.g., national digital infrastructure; and 4) Implementation Process: The activities and strategies used to carry out the innovation, e.g., assessing available technology, planning, staff training and capacity-building [22].

Results

Formatted: Font: 18 pt

Sample characteristics

Formatted: Font: 16 pt

Health

Primary health care centers (53%) and health posts (23%) comprised the majority of the sample (N = 1877) (**Error! Reference source not found.**). Consistent with this, over three-quarters of institutions represented were primary care level institutions (7876%). Peru (23%) and Ecuador (21%) made the largest proportion of the sample, followed by Bolivia (16%).

Table 1. Use of information and communication technologies (ICTs) in the provision of maternal health servicesmaternity care in selected countries of Latin America and the Caribbean, by type of institution and country (2021-2022)), based on survey responses

Deleted Cells

Formatted Table

Variable	Total	Used ICTs (% by column)	Used ICTs (% by row)

	n (%)		~~%~~
	**1877 (100.0)**	**1536 (81.8)**	
**Institution type**			
Referral hospital	160 (8.6)	150 (9.8)	~~93.8~~
District/regional hospital	180 (9.7)	127 (8.3)	~~70.6~~
~~Health~~Primary health care center or clinic	990 (53.1)	794 (52.0)	~~80.2~~
Health post	422 (22.6)	360 (23.6)	~~85.3~~
Administrative center/health network	113 (6.1)	96 (6.3)	~~85.0~~
**Level of care**			
Primary	1440 (78.1)	1175 (77.8)	~~81.6~~
Secondary	230 (12.5)	176 (11.7)	~~76.5~~
Tertiary	145 (7.9)	135 (8.9)	~~93.1~~
**Country**			
Argentina	73 (3.9)	56 (3.7)	~~76.7~~
Bolivia	305 (16.3)	254 (16.5)	~~83.3~~
Colombia	212 (11.3)	183 (11.9)	~~86.3~~
Dominican Republic	39 (2.1)	25 (1.6)	~~64.1~~
Ecuador	402 (21.4)	277 (18.0)	~~68.9~~
Guyana	72 (3.8)	50 (3.3)	~~69.4~~

Deleted Cells

Split Cells

Formatted: Font: 10 pt, Not Bold, Font color: Auto

Formatted: Left

Formatted Table

Formatted Table

Honduras	83 (4.4)	58 (3.8)	~~69.9~~
Paraguay	260 (13.9)	219 (14.3)	~~84.2~~
Peru	431 (23.0)	414 (27.0)	~~96.1~~

Use of ICTs

Formatted: Font: 16 pt

Overall, 82% (n = 1536) of health facilities reported using ICTs in maternity care (~~Table~~Fig 1). Peru was the country with the highest proportion of facilities reporting ICT use (96%), followed by Colombia (86%). The Dominican Republic was the country with lowest proportion of facilities reporting ICT use (64%). When examining ICT use by institution type~~,~~ and care level, the highest proportion of ICT use was indicated by referral hospitals (94%) and tertiary care level centers (93~~%).~~%), respectively. In comparison, 80% of primary health care centers used ICTs.

Fig 1. Proportion of institutions that used ICTs, by country (2021-2022), based on survey responses (N = 1877)

Family planning (64%) and breastfeeding (58%) counseling were the services most commonly provided through the use of ICTs (Table 2). The services less frequently offered via ICT were safe abortion counseling (9%), medical prescription orders (24%), and telemonitoring or long-distance monitoring of high-risk conditions using applications or digital devices (25%). Of note, the proportion of facilities that reported ICT use ~~ICTs~~ to provide safe abortion counseling was higher in countries where the procedure is legal in all or certain circumstances, Argentina (36%) and Colombia (30~~%)~~%), and lowest where it is highly criminalized, such as Honduras (0%) (See

~~Annex E~~S3 Table. Use of ICTs for abortion counseling by country). Other services mentioned included teleconsultations between professionals, management of domestic violence situations, management and treatment of sexually transmitted infections, and ~~the follow-up~~care of pregnant women with COVID-19 infection, among others.

In terms of the ICTs used in maternity care, an overwhelming majority of facilities ~~(82%)~~ used the phone~~,~~ (82%), including applications that enable calling, to communicate with clients (Table 1). To a much lesser extent, other ICTs used included electronic medical records to access client health data (27%), a hotline or dedicated phone number to address pregnancy concerns (27%), and video calls for telemedicine consultations (27%). Provision of telemonitoring devices to pregnant women and engaging in remote recording and monitoring of pregnancy-related conditions, such as blood sugar levels or blood pressure measurements, by providers were not commonly used. ~~In the open text under "Other", respondents provided more details on the services provided, such as using telephone landlines to check on pregnant women during COVID-19,~~Other responses included using social media (such as Facebook), and national health information platforms (such as WawaRed in Peru ~~[23]~~[22] or the Federal Platform for Telehealth and Distance Communication in Argentina ~~[24]).~~[23]). Digital health norms and protocols are important to ensure quality of telecare and compliance with ethical standards. Among respondents, 38% of institutions had a telehealth protocol in place and 50% had systems to ensure data security.

Table 1. Maternity care and information and communication technologies (ICTs) in nine Latin American and Caribbean countries (2021-2022~~)~~), based on survey responses

Variable	Proportion<br>N = 1494 (100.0)*
**Types of services provided using ICTs**	
Safe abortion counseling	134 (9.0)
Orders for medical prescriptions	361 (24.2)
Telemonitoring	374 (25.0)
Gestational disease management	459 (30.7)
Diagnostic antenatal care visit	571 (38.2)
Birth preparation	585 (39.2)
Routine postnatal care visit	628 (42.0)
Routine antenatal care visit	779 (52.3)
Breastfeeding counseling	867 (58.0)
Family planning counseling	962 (64.4)
~~Others~~Other	357 (23.9)
**ICTs used****	
Telemonitoring applications	88 (6.0)
Email address for pregnancy concerns	92 (6.2)
Provision of phones or data to pregnant women	118 (8.0)
Provision of telemonitoring devices to pregnant women	122 (8.3)
Web-based platform for data sharing	185 (12.6)
mHealth (text messages)	217 (14.7)

Variable	Proportion<br>N = 1494 (100.0)*
Video calls for telemedicine consultations	392 (26.6)
Hotline to address pregnancy concerns	397 (26.9)
Electronic medical records	404 (27.4)
Phone calls, including via Apps, for consultations	1205 (81.8)
Other	133 (9.0)
**Protocols and data security*** **	
Telehealth protocol in place	378 (27.7)
Data security system	664 (49.6)

Note: *Does not add to 100 because multiple options were possible; ** n = 1,474 due to missing; *** n = 1363 and 1339, respectively, due to missing

Formatted: Font color: Auto

Formatted: Font color: Auto

Formatted Table

Formatted: Font: Bold, Font color: Auto

Formatted: Font color: Auto

Formatted: Indent: First line: 0 ch

Formatted Table

Interviewees provided more nuanced descriptions of how ICTs were used to provide services to women during pregnancy and the puerperium. Interviewees described using ICTs for scheduling appointments, to conduct routine antenatal visits, for remote gestational disease management, and for medication prescribing. In Colombia national guidelines during COVID-19 recommended antenatal teleconsultations to conduct anamnesis, maternal risk assessment~~,~~ and monitoring, behavioral counseling ~~on healthy behaviors, monitoring of signs of danger~~, and laboratory analysis requests ~~[25].~~[24]. Protocols of a tertiary maternity hospital in Peru for remote antenatal care visits recommended similar services, but also included orders for ~~laboratory tests and~~ imaging, as relevant to gestational age, as well as COVID-19 tracing [15].

Interviewees in Peru considered having electronic medical records, which providers could access from home, if needed, as a key ~~enabling factor for~~enabler of teleconsultations.

In addition to direct services, an interviewee from the Ministry of Health of Bolivia, considered ICTs critical to coordinate obstetric emergency responses. He described that staff in referral and emergency coordinating centers in each region used cell phones to coordinate the response to obstetric emergencies and referrals, including ambulance dispatch. Other interviewees from Bolivia and Paraguay mentioned the development of geospatial analysis tools to inform reproductive health supply chain management in remote areas based on the numbers of reproductive-aged and pregnant women. In both cases, the development of these tools was funded by international development and tested as pilot trials. They were complex and resource intensive interventions, and whereas promising in small scale implementation, they proved difficult to sustain, scale-up and integrate into public healthcare systems.

Interviewees from Argentina, Colombia, Peru and Paraguay mentioned the phone as the most common ICT used to reach pregnant women, ~~either via a direct~~both for phone ~~call to follow-up on a client, or by~~calls and text ~~to send reminders~~messaging. In Peru and Paraguay, nurse midwives mentioned forming WhatsApp groups of pregnant women to provide healthy behavior recommendations and to answer questions. A midwife in Paraguay explained, "*each midwife had her own list of patients. [Providers] became more accessible to clients by using WhatsApp to answer concerns and to discuss medical lab results.*" Interviewees described how their facility used social media tools (such as Facebook) for risk communications, to share facility ~~news,~~updates, to provide general health recommendations ~~for~~to pregnant women (e.g., vaccination reminders), and for ~~events~~event announcements (e.g., birth preparation workshops).

Interviewees in Peru and Argentina described using ICTs, specifically Zoom and YouTube, to offer birth preparation classes. A midwife in Peru recounted opening ~~a~~ an educational YouTube channel ~~to provide educational classes~~ for pregnant women and recent mothers ~~led by~~featuring different specialists, such as pediatricians, endocrinologists, and nutritionists, among others.

Factors associated with ICT use

Formatted: Font: 16 pt

~~Respondents were offered a list of five social and nine institutional factors commonly associated with ICT use and asked to rate the extent to which each facilitated the adoption of ICTs. Respondents perceived ICT acceptability by users (26%) as the strongest social factor that facilitated its use.~~In terms of strategies implemented by institutions to promote ICT use, overall, 45% of facilities indicated providing some support to staff (). Of these, 46% trained staff on ICT use, either through in-house programs or by collaborating with health authorities. Only a small percentage of facilities (6%) reimbursed providers for costs incurred when using personal devices or the internet, and 16% furnished providers with cell phones or other mobile devices for telehealth. Roughly 24% of facilities had dedicated IT personnel to address any technological issues encountered while using ICTs, and 60% had a designated telehealth focal point who served as a point of contact for coordinating telehealth use and addressing provider inquiries.

Respondents perceived ICT acceptability by users (26%) as the strongest facilitating social factor. Conversely, cost (37%) and accessibility of ICTs (30%) were the two factors perceived to least facilitate ~~the adoption of ICTs (Figure 1).~~ICT adoption (Fig 2). In terms of institutional factors, 38% of respondents indicated that providers trained in the use of ICTs, and 28% that the availability of adequate equipment and technology, were the main facilitators of ICT adoption.

Conversely, 49% of respondents perceived that funding for telehealth ~~(49%)~~ was ~~the least~~not a facilitating factor. This is not surprising, as only 9% of institutions reported receiving such funding. These digital health initiatives were mostly funded by the public sector (83%). Among these, over half (56%) received the funding during the COVID-19 pandemic.

Formatted: Font color: Black

Formatted: Font: 12 pt, Font color: Black

Fig 2. Social and institutional factors associated with ICT use among institutions that used ICTs, by level of importance.

Table 2. Staff support and funding to support ICT use in maternity care among facilities that used ICTs, 2021-2022 (n = 1391)

**Staff support**	**n (%)**
Facility supported staff	625 (44.9)
Trained providers in ICT use	643 (46.2)
Reimbursed personal internet costs*	39 (6.2)
Distributed cell phones to providers*	103 (16.5)
IT staff assigned to solve problems*	147 (23.5)
Designated telehealth focal point*	378 (60.5)
Institution received funding for telehealth	117 (8.7)
During the pandemic**	65 (55.6)
Public funds**	98 (83.8)

*Percentage calculated over number of institutions that provided support (n = 625);

**Percentage calculated over those who received funding (n = 117)

Formatted Table

Formatted: Font: 11 pt, Not Bold, Font color: Auto

Qualitative data provided more context to the adoption of ICTs. There were mixed responses related to willingness of providers and patients to use ICTs. Some interviewees mentioned that providers and users were open to using ICTs, particularly cell phones which are ubiquitous in many Latin American contexts, to provide and receive services. An interviewee from Bolivia explained, *"[using ICTs] is acceptable to many women by telephone. And it is ~~accesible~~<u>accessible</u>. Many users have cell phones with WhatsApp."* ~~Another~~<u>An</u> interviewee from Paraguay noted that communicating with providers by message, calls, and videocalls was acceptable to users but, *"it's very precarious, there are few phones available, and the lines become very slow."* However, other interviewees mentioned that providers were reticent to adopt new technologies in their practice. One interviewee described, *"The obstetricians were afraid of giving advice on Zoom. We had to offer a tutorial on the ABC of using Zoom to get them to participate."* In this sense, several interviewees mentioned that capacity building on the use of ICTs, including on protocols for their use, and having IT staff available to solve technological issues, were necessary to promote their adoption. A barrier mentioned by an interviewee from Bolivia was the lack of computer science engineers working at the Ministry of Health. Some interviewees explained that many low-income households access internet via a cell phone, ~~which is~~ the only digital device at home. Thus, to receive phone calls or participate in online sessions, women had to negotiate the use of the cell phone with their partners, and even their children. One interviewee described, "*We had to really work on empowering women to talk to their husbands and convince them that it was important to leave the cell phone with internet at home."*

According to interviewees, having the digital infrastructure in place~~,~~ was a determinant of ICT use. In Argentina and Peru, for example, the government has invested in building the digital

infrastructure for decades. Most public facilities are equipped with internet connection and IT equipment, such as computers. Instead, in Guyana, Honduras, and Paraguay, interviewees mentioned that some regions do not have internet coverage and that many primary care facilities in the public sector lack basic digital infrastructure. A midwife from Paraguay, for example, mentioned staff having to pay out-of-pocket ~~to have~~for the internet at ~~their workplace~~work. She explained, *"A group of 22 nurse midwives came together to pay for the monthly internet fee, this allows us to upload productivity, use email, connect via WhatsApp."* One of the biggest challenges reported were the areas with poor internet connection and intermittent connectivity. Interviewees perceived this as a source of disparities, as ~~these issues~~this issue mainly ~~affected~~pertained to rural areas. An interviewee from a rural municipality in Colombia described the challenges of building telehealth services~~ in such areas~~, "*Sometimes there are people who have to climb a hill or climb a tree to be able to catch a signal and sometimes the calls are lost.*" In Paraguay, an interviewee indicated that service providers serving indigenous communities did not use digital health at all given ~~that internet was not accessible in these areas and the dearth of~~lack of connectivity and IT equipment.

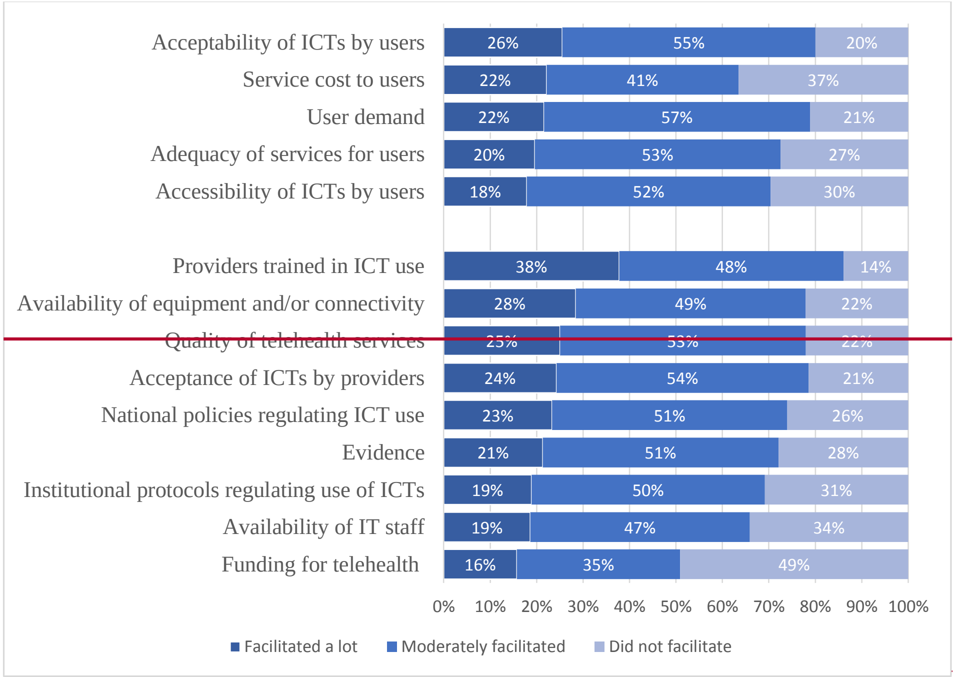

Figure 1. Social and institutional factors associated with ICT use among institutions that used ICTs, by level of importance

Formatted: Font: Bold

Digital health and the COVID-19 pandemic

Formatted: Font: 16 pt

During 2020, the COVID-19 pandemic disrupted health services and presented access to care barriers to pregnant and postpartum women. We asked respondents in facilities that used ICTs, the extent to which their use changed as a result of the pandemic. Overall, 42% of respondents said that their ICT use did not change since the COVID-19 pandemic, 39% that they used ICTs more than prior to the pandemic, and 20% that they started using ICTs as a result of the

pandemic ~~(Figure 2).~~(Fig 3). Honduras (76%) and the Dominican Republic (68%) had the highest proportion of facilities that did not change their ICT use during the pandemic; facilities in Argentina (63%) and Paraguay (46%) were the most likely to report using ICTs more than before COVID-19~~,~~; and ~~in~~ Peru ~~32% of~~was the country with more facilities indicating using ICTs for the first time~~.~~ (32%). When asked if they would continue using ICTs~~,~~ in the future, the majority of respondents (84%) indicated they would. The proportion that indicated this was marginally larger in the group who ~~indicated using~~used ICTs more than before the pandemic (90%) (~~See Annex F~~S1 Graph. Future use of ICTs among respondents who used them). One interviewee reflected, *"telehealth will continue in part because it gives us greater flexibility, but we still have to address the problem of connectivity in remote districts."*

Formatted: Font color: Custom Color(RGB(34,34,34))

Formatted: Adjust space between Latin and Asian text, Adjust space between Asian text and numbers

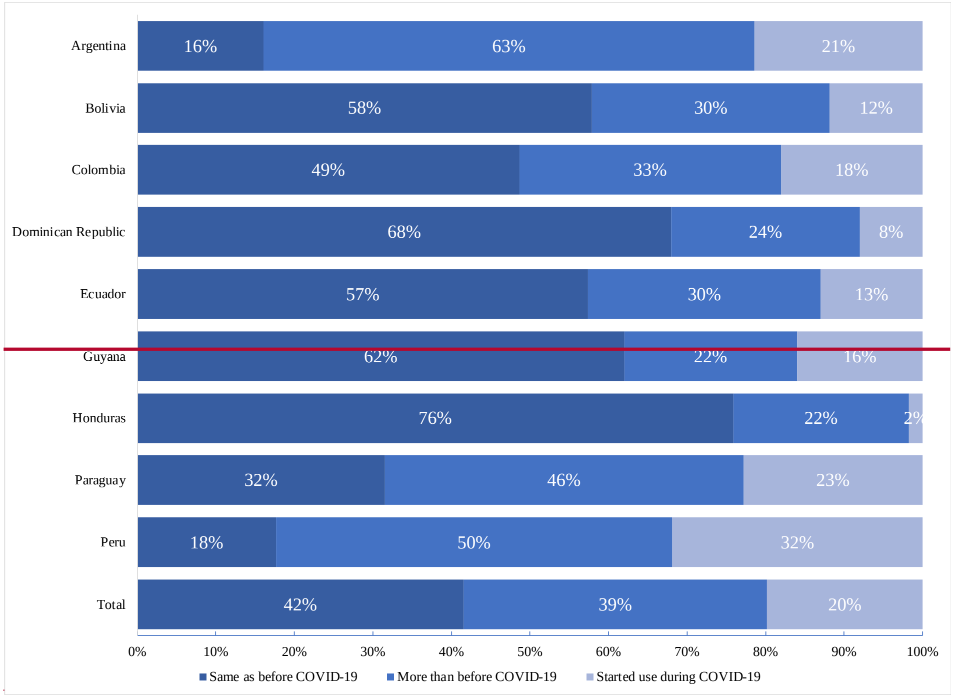

~~*Figure 2.*~~**Fig 3. ICT use in response to COVID-19**

Formatted: Font: Times New Roman, 12 pt, Bold, Not Italic, Font color: Auto

Formatted: Font: 12 pt, Not Italic, Font color: Auto

~~Based on qualitative data we identified different~~Qualitative analysis revealed multilevel approaches ~~taken at different levels - national, institutional, and individual level~~ to minimize ~~the risk of~~ COVID-19 infection risk among pregnant women and providers while maintaining continuity of care. ~~Some examples drawn from the interviews are detailed below.~~

At the national level, the Ministry of Health and Social Protection of Colombia issued protocols for the provision of maternity and infant care during the emergency. The protocols recommended a hybrid model of antenatal visits that involved a combination of antenatal teleconsultations

(25%), home visits (25%), and face-to-face consultations (50%) ~~for antenatal care [25].~~[24]. According to an interviewee, disparities emerged in the implementation of this model. Whereas healthcare providers in the private sector had the infrastructure to provide telehealth consultations, they ~~tended to overuse~~overused them to reduce costs~~ without~~, not properly evaluating obstetric risks~~ and gestational age~~. On the other hand, institutions in the public sector faced challenges in implementing the remote visits, even via telephone, due to a lack of connectivity and equipment. The hybrid antenatal care model was discontinued in 2022 with the end of the pandemic.

In Argentina, the ministry of health extended a telehealth platform used for consultations between providers for management and ~~referrals~~referral of patients with possible COVID-19 infection. The main use of the platform was to evaluate suspected COVID-19 cases and guide the diagnosis and treatment of the patients. Teleconsultations were offered in real-time via videoconferencing. Whereas the platform was not specific to ~~maternal health~~maternity care, pregnant women and recent mothers were considered a priority group.

At the institutional level, a tertiary care maternity hospital in Peru implemented a hybrid antenatal care model for high-risk pregnancies, which entailed alternating consultations and face-to-face visits (50%/50%) [15]. Reflecting on the model's implementation, an administrator noted the importance of an institutional culture of innovation and prior experience in using ICTs to build upon, *"We were the first institution [in the country] to use telehealth. In 2007, we created the maternal-perinatal telehealth network with the institution's own funds, which facilitated interconsultations between our hospital and other facilities throughout the country... When in-*

person consultations closed on March 15, 2020, we began testing a telehealth model." Interviewees considered the use of electronic medical records as a facilitating factor, which enabled providers to access patients' clinical histories from home. Another facilitating factor was having IT personnel on staff to support providers in case of technical difficulties. The hybrid model was widely accepted by users and has expanded since its launch in 2020 to include an app. One limitation of the approach was that it was mainly applied in urban areas of Lima, with good connectivity, in a highly equipped institution, and whose users had access to cell phone or other digital devices.

Another institutional example was a maternity hospital in Argentina which began offering its once in-person birth preparation classes virtually via Zoom, while continuing the normal in-person schedule of antenatal care visits. The modality was highly feasible and acceptable to users. Further, the approach resulted in more family members participating compared to the traditional face-to-face model.~~ In addition, to~~

To reduce crowding, facilities in several countries began scheduling antenatal care visits with a set day and time by phone. Prior to the pandemic, providers would only give women a day or week for a return visit. However, interviewees noted that scheduling in advance was difficulted by having physical rather than digital agendas, which made it hard to coordinate scheduling across providers, by women not coming to the scheduled visit, by problems anticipating how many patients would come in person without an appointment, and by problems rescheduling in case of an unexpected occurrence (e.g., if the provider is unavailable). Further, some facilities that ~~gave~~instructed women ~~a phone number~~ to make appointments~~, found that that they~~ by

phone, did not have enough human resources to answer the phone. As a result, users spent long hours on the phone trying to schedule or reschedule appointments.

In other cases, ICT use was adopted informally by individuals. In institutions across several countries, nurse midwives began calling their patients to check on them. An interviewee from Argentina explained, *"[Concerned with the lack of controls because of the pandemic...], and having the perinatal clinical history available, midwives began offering teleconsultations to pregnant women."* These informal approaches, however, were used temporarily and in response to the emergency and were largely discontinued.

Formatted: Font color: Custom Color(RGB(19,20,19))

~~**Institutional factors and strategies enabling ICT use**~~

~~We asked facilities about the institutional factors that may have contributed to the use of ICTs in maternity care and about some of the strategies that they implemented. Overall, 45% of facilities indicated providing some support to staff (Table 3). Of these, 46% trained staff on ICT use, either through in-house programs or by collaborating with health authorities. Only a small percentage of facilities (6%) reimbursed providers for costs incurred when using personal devices or the internet, and 16% furnished providers with cell phones or other mobile devices for telehealth. Roughly 24% of facilities had dedicated IT personnel to address any technological issues encountered while using ICTs, and 60% had a designated telehealth focal point who served as a point of contact for coordinating telehealth use and addressing provider inquiries. Regarding the allocation of funding earmarked for digital health, only 9% of institutions reported receiving such funding. Among these, over half (56%) received the funding during the COVID-19 pandemic. These digital health initiatives were mostly funded by the public sector (83%). In~~

Field Code Changed

addition, 38% of facilities had protocols in place to regulate ICT use; 50% had systems to protect sensitive patient health information; and 35% used electronic medical records.

Table 3. Institutional-level factors and strategies to support ICT use in maternity care among facilities that used ICTs, 2021-2022 (n = 1391)

Staff support	45%
Trained providers in ICT use*	46%
Reimbursed personal internet costs*	6%
Distributed cell phones to providers*	16%
IT staff assigned to solve problems*	24%
Designated telehealth focal point*	60%
Enabling institutional factors	
Institution received funding for telehealth	9%
During the pandemic**	56%
Public funds**	83%
Institution has a telehealth protocol in place	38%
Institution has systems to ensure data security	50%
Institution has electronic medical records	35%

*Percentage calculated over number of institutions that provided support (n = 625); **Percentage calculated over those who received funding (n = 117)

Formatted Table

Formatted: Font: 11 pt, Not Bold, Font color: Auto

National digital and economic setting

Formatted: Font: 16 pt

To better understand the outer setting in which ICT interventions were implemented in maternity care, this section presents data on economic markers and the national digital infrastructure, including digital coverage, penetration, and affordability. As can be seen in Table 3, all the countries considered in this study are middle-income economies, with Bolivia and Honduras categorized as lower-middle-income and the other countries as upper-middle-income. In terms of digital coverage, most of the population in the countries under study are covered by basic mobile-cellular network. However, gaps remain, particularly in rural areas, with more than 10% of the population not covered by 3G mobile-cellular network in Bolivia, Honduras and Peru. As described above, interviewees in many hard-to-reach areas noted that internet connection was unstable or difficult to access. When examining internet use, we observe large differences by country, with the percentage of the population using the internet ranging from 87% in Argentina to 48% in Honduras. In terms of access to ICTs at home, with the exception of Argentina where the government has actively sought to universalize access by distributing personal computers to public school children, a majority of households do not have home computers. Internet at home is primarily accessed through mobile phones.

Mobile-broadband subscriptions may include three distinct services: voice, SMS messaging and data. Table 3 presents the monthly cost (expressed as % of per capita GNI) of a basic voice and SMS only (no data) basket and of a high-consumption bundle with an allowance of up to 140 minutes of calls, 70 SMS messages, and 2 GB of data. Many of the countries under study lag behind in affordability, with Colombia and Peru being the only two countries where the cost of subscriptions is below the 2% affordability threshold.

Table 3. National digital and economic setting, 2021

Country	Infrastructure & Access					Affordability		Country Income
	Network Coverage		% Population using the Internet	ICTs at Home				
	Population covered by mobile network	Population covered by 3G mobile network	~~% Population using the Internet~~	Internet access at home	Computer at home	Mobile high consumption basket as % of GNI	Mobile voice and SMS (no data) basket as % of GNI	~~Country Income~~
Argentina	98%	98%	87%	90%	64%	4.0%	2.1%	Upper-middle
Bolivia	100%	88%	66%	57%	28%	7.9%	3.6%	Lower-middle
Colombia	100%	100%	73%	61%	38%	1.9%	1.1%	Upper-middle
Dominican Republic	100%	99%	85%	46%	43%	4.4%	1.6%	Upper-middle
Ecuador	96%	95%	70%	60%	40%	3.2%	2.8%	Upper-middle
Guyana	97%	93%	85%		41%	5.7%	2.8%	Upper-middle
Honduras	89%	82%	48%		16%	10.5%	7.2%	Lower-middle
Paraguay	99%	95%	77%	45%	28%	3.0%	3.0%	Upper-middle

Peru	89%	87%	71%	49%	34%	1.7%	1.7%	Upper-middle
Regional	96%	92%	81%	75%	60%	3.0%	2.0%	

~~*Note:*~~ Source of digital environment data: International Telecommunication Union (ITU); Country income based on the World Bank classification. For Infrastructure and Access, the values below the regional average are marked in red; for Affordability values above 2%, the global affordability threshold, are marked in red.

Formatted: Line spacing: single

Formatted: Line spacing: single

Formatted: Line spacing: single

Discussion

Formatted: Font: 18 pt

This mixed methods ~~assessment~~study was conducted in nine middle-income Latin American and Caribbean countries to understand how, primarily public sector, facilities used ICTs to provide maternity care. Because it was conducted following the COVID-19 pandemic, we were able to examine the extent to which digital health interventions were implemented in response to the sanitary crisis. We found that ICTs were used for various services along the continuum of antenatal to postnatal care and differed across countries and contexts. Overall, 4 out of 5 institutions reported incorporating ICTs in some way in maternity care. The widespread use of digital health is not surprising given the region's commitment to advancing universal health coverage through technology ~~[26].~~ [25]. Of note, we also found that a digital divide remains, with some populations still unable to benefit from technological developments.

The most frequent ~~use~~uses of ICTs ~~was~~were to provide family planning and breastfeeding counseling over the phone, a largely available technology in the region. These findings are in line with those of a global survey of ~~maternal~~maternity health professionals, which found that among providers using ICTs, common uses included routine antenatal care (65%), child preparation classes (59%), breastfeeding counseling (50%), and family planning counseling (40%) ~~[10]~~[12].

~~Consistent with the literature [27, 28], COVID-19 seems to have accelerated the use of digital health. We found that one-fifth of facilities that used ICTs did so for the first time during the pandemic. At times, the choice of using ICTs was the provider's personal decision in response to disruptions in care posed by the pandemic and an inability to reach patients. In most of these~~

cases, providers used their personal devices and assumed the data and connectivity costs. In other cases, incorporating ICTs in maternity care was an institutional decision made by administrators. Inner setting factors related to staffing, such as having IT personnel in place to support this process and to troubleshoot the technological problems encountered, and training healthcare providers on how to use the technology and/or deliver the digital intervention, emerged as important facilitators. In addition, many countries issued policies and guidelines for the use of ICTs during COVID-19. Characteristics of the outer setting were also of utmost importance. In particular, the adoption of digital health at the facility level, depended largely on preexisting country commitment to building the digital infrastructure, evidenced in nearly universal broadband coverage, existing digital healthcare platforms, and public sector facilities with IT equipment. As a result, facilities in countries with a history of investment in developing the digital infrastructure faced fewer barriers to the adoption of ICTs in maternity care than those without. Overall, our findings are consistent with prior surveys, finding that the digital infrastructure and support for telehealth programs, including funding, were the top factors determining their use [6]. Of note, despite these important investments, this study also shows that a digital divide remains, with some populations still unable to benefit from technological developments.

Implementation science provides a useful framework to understand factors influencing the adoption of ICTs in maternal health and is helpful to identify the most promising strategies to overcome barriers to ICT use. Below, we detail the most salient issues that emerged from this study in the following domains, and presented graphically in Figure 3: 1) individuals and health teams in roles that affect the use ICTs, 2) characteristics of the ICT strategy used, 3) the inner or

~~facility-level setting, and 4) the outer or environmental setting. At the individual or team level, three important individual roles affected the adoption of ICTs in maternity care: the health professionals tasked with using ICTs to deliver care or make data-driven decisions; the department and facility leaders; and IT personnel as implementation supports. Among maternal health professionals, we found a wide range of attitudes related to ICT use~~Consistent with the literature [26, 27], COVID-19 seems to have accelerated the use of digital health. We found that one-fifth of facilities that used ICTs did so for the first time during the pandemic. Factors associated with the use and adoption of ICTs in maternity care can be classified into individual or team, institutional, and external-level factors, as described below.

Individual and health team-level factors

We found a wide range of attitudes related to ICT use among providers, from reticence to change to willingness to adopt new technologies and modify existing protocols. The barriers to access created by COVID-19 acted as an incentive for individual providers and health teams to embrace change. In fact, we documented several instances in which ~~maternal health~~maternity care providers initiated the use of ICTs to reach pregnant women during COVID-19. Their initiative, motivation, and creativity were critical to the use of ICTs~~ in many instances.~~. Often, the reticence to ICT use ~~documented ~~was~~ partially~~ related to low perceived self-efficacy and lack of skills to use new technologies. Indeed, provider training in the use of ICTs was ranked as the leading facilitating factor for their adoption. The adoption of ICTs during the COVID-19 pandemic occurred within a high-stress context, with healthcare providers experiencing burnout, uncertainty, and fear. Learning new processes and acquiring new skills is time-consuming, time which providers did not have during the sanitary emergency. ~~While individuals and health teams~~

drove interventions, institutional leadership was crucial for the adoption of ICTs in a sustainable way. In particular, maternal health department heads and institutional directors facilitated the adoption of ICTs in maternal health through the allocation of budgets and human resources, including designating a telehealth focal point, hiring IT staff, and allotting provider time for training. Individuals in leadership positions also influenced the adoption of ICT by fostering interdepartmental coordination, influencing the institutional culture in favor of innovation, and issuing policies and protocols for the use of telehealth, which built confidence among providers. In sum, leadership supports helped to create a favorable institutional environment for the adoption of ICT interventions. Finally, the availability of IT personnel to develop new technologies, train providers in their use, and problem-solve were critical to the institutional adoption of ICTs.

Formatted: Font color: Custom Color(RGB(19,20,19))

Institutional-level factors

While the contributions of individual and health teams were important, perhaps the determining institutional factor affecting the use of ICTs was the information technology infrastructure. Different strategies require different structures, but at a basic level, inadequate telecommunication systems, including reliable internet connection and mobile network signal, was a major barrier to the adoption of ICTs. Lack of ICT equipment, including computers or tables, as needed, was another barrier.

We also found that, often, the workflows, processes, and staffing structures were not compatible with the use of ICTs. Using ICTs requires adequate staffing for providers to have capacity to care for both remote and in person patients, and for support staff, including nursing and

administrative staff, to arrange scheduling and pull up patient records. Finally, supervisors should be available to provide guidance and feedback on telehealth provision; and systems must be in place to count telehealth services towards the provider's productivity. In the context of scarce resources, providing basic health services was the priority, with limited opportunities to allocate the human, time and financial resources to fully embrace an innovation.

Formatted: Font color: Custom Color(RGB(19,20,19))

The characteristics of the ICT strategy used were also important factors in their use or not in maternal health. The key ICT-level characteristics identified were the strength of the evidence base, their availability and accessibility, level of complexity, and their cost. The evidence for use of ICTs in maternal health in middle-income countries is promising but not robust. Indeed, WHO guidelines recommend that virtual antenatal visits do not replace, but rather complement, in-person visits [29]. A systematic review of ICT use in sexual and reproductive health services in Latin America and the Caribbean found several promising interventions but insufficient evidence related to outcomes [14]. For example, a randomized controlled trial in Peru to test the effectiveness of SMS for antenatal appointment reminders, significantly increased the number of antenatal visits among women in the trial condition, but there were no differences in pregnancy outcomes [30]. One successful example was the implementation of a model based on telehealth and education for patients with obstetric emergencies in two rural regions of Colombia, which resulted in a 29% reduction in perinatal deaths [31]. Similarly, the examples from Peru and Colombia of reduced-visit antenatal care models reported in our study were acceptable to providers and users but did not have information on outcomes. Availability, accessibility, complexity and cost were also important factors. The phone, as mentioned previously, was already available and widely used for personal purposes, so could be easily repurposed for

telehealth. Furthermore, people are familiar with the use of that technology and the cost of basic SMS and voice services is not prohibitive. We found fewer examples of more complex systems and platforms that require substantial financial investment and time in their development and implementation. Even electronical medical records were only available in about a third of institutions. Further, less than 1 in 10 institutions received funding specifically for telehealth. Therefore, facilities or providers had to invest of their resources to use ICTs. Implementing complex interventions requires substantial planning, coordination of multiple partners, and training. Health data sharing, for instance, requires systems that are secure and interoperable across institutions. The more complex the system or platform, the more resources and time are needed for its development and implementation. This complexity can be a barrier to adoption, particularly in resource-constrained settings where institutions may lack the necessary capacity and expertise. In our study, interviewees reported that apparently simple interventions faced challenges. For example, facilities trying to implement advanced scheduling of antenatal care visits by phone encountered insufficient human resources to schedule and/or to respond to requests to reschedule visits, and difficulties presented by no-shows and by clients without appointments.

In terms of the inner setting or at facility-level, a few salient factors affecting ICT use emerged from the current study. The primary factors affecting the use of ICTs was the information technology infrastructure. Different strategies require different structures, but at a basic level, inadequate telecommunication systems, including reliable internet connection and mobile network signal, was a major barrier to the adoption of ICTs. Lack of ICT equipment, including computers or tables, as needed, were another barrier. For example, to carry out teleconsultations

between providers, having interoperable systems in place to share patients records securely, including laboratory results and imaging, is critical. In addition, sufficient staffing levels that allow providers to have sufficient time to provide telemedicine and also attend to patients coming in person are important. As well as having, sufficient support staff, including nursing and administrative staff, to arrange scheduling and pull up patient records, is important to be able to provide telemedicine. The needed staffing structure to adopt telemedicine is not in place in many facilities. In some cases, the use of the proposed ICT strategy was not compatible with existing workflows and processes. Supervision, for example, is not often set up to provide guidance and feedback on telehealth provision; other times, systems are not set up to count telehealth services towards the provider's productivity. Lack of funding was another factor limiting adoption of ICTs as very few of the institutions surveyed allocated or received funding to purchase equipment, develop or adapt technological platforms, etc. Interviewees mentioned several pilot projects funded by international development, but they were limited in scope and lacked subsequent scale-up or long-term sustainability. In some cases, providers were asked to use a new platform or provide a service using ICTs without the appropriate training and supports (e.g., IT staff, telehealth focal point). In the context of scarce resources, providing basic health services was the priority, with limited opportunities to allocate the human, time and financial resources to fully embrace an innovation.

Formatted: Font color: Custom Color(RGB(19,20,19))

Factors at the outer or environmental setting National-level factors

Ultimately, national-level factors were the most important determinants of ICT use.

Undoubtedly, the COVID-19 pandemic was a critical incident that disrupted the provision of care, triggering the need for innovative ways to reach patients at a distance. However, the ability

of individuals and facilities to incorporate new technologies in maternal health during the pandemic wasThe adoption of digital health at the facility level depended largely based on prior commitmentspreexisting country commitment to achieveachieving universal health coverage on one hand and universal digital access, on the other. Thus, facilities in countries that had historically invested in and building the digital infrastructure, evidenced in nearly universal broadband coverage, existing digital healthcare platforms, and public sector facilities with IT equipment. Facilities in countries with a history of investing in developing the digital infrastructure to ensure access to the entire population and that had begun to build national digital health platforms, were faced fewer barriers to the adoption of ICTs in maternity care than those best placed to bridge the care gap during times of crisis. without. For example, to carry out teleconsultations between providers in different institutions, interoperable systems to share patient records securely, including laboratory results and imaging, must be in place. Among institutions surveyed, most of the funding to expand ICT use received was public and received during the pandemic, highlighting the importance of government investments. PolicyOverall, our findings are consistent with prior surveys, that identified the national digital infrastructure and government support for telehealth programs, including public funding, as the top factors determining their use [6].

Another factor impacting the adoption of ICTs was the lack of a policy framework to regulate the use of ICTs, particularly of direct provider-patient care, in many Latin American and Caribbean countries at the beginning of the pandemic [28]. Whereas policy regulations around telehealth were lagged behind practice; however, several governments issued new policies to regulate

Formatted: Font color: Custom Color(RGB(19,20,19))

Formatted: Font color: Custom Color(RGB(19,20,19))

Formatted: Font color: Custom Color(RGB(19,20,19))

Formatted: Font color: Custom Color(RGB(19,20,19))

Formatted: Font color: Custom Color(RGB(19,20,19))

Formatted: Font color: Custom Color(RGB(19,20,19))

telehealth during the COVID-19 ~~[32]~~[29], including mandates to public health insurances to reimburse remote care provision ~~of services remotely~~.

Strengths and limitations

Formatted: Font: 16 pt

This study findings should be considered in the light of some limitations. Despite efforts to obtain a representative sample of facilities in each county, it should be noted that facilities in distant areas and areas with low internet connectivity were harder to reach. This bias is particularly pertinent to the subject of this analysis, as we may have over-represented the use of ICTs in maternal health. Further, response rates varied by country, and were particularly low in the Dominican Republic. To mitigate this bias, national consultants travelled to hard-to-reach areas to administer the survey in person and/or called potential respondents to administer the survey verbally. Through our extensive outreach, we were able to obtain a large number of responses, making our findings robust. Because we limited the sample to facilities that provided antenatal care, a majority of institutions represented were primary care institutions. A sample with a larger representation of tertiary care facilities may have yielded different findings, such as more frequent use of complex technologies to provide care. The survey was validated with healthcare providers in each country. However, there may have been differences among respondents in the interpretation of the survey questions, thus introducing measurement errors. The mixed methods study focused on the experiences of health providers and administrators primarily in public sector facilities that serve the highest-need populations. Future research could focus on user experiences, such as the satisfaction, acceptability and cultural appropriateness of using ICTs to receive maternity care. Another limitation is the cross-sectional design, which captured the experiences of facilities at a specific moment in time. The responses illustrated how

ICTs were used at the time of the data collection and the changes in their use during the pandemic. However, it was not clear how widely these had been used prior to the pandemic or the extent to which they will continue to be used after the resolution of the sanitary crisis. Future longitudinal research would provide valuable insights into the continuity and evolution of ICT use in maternity care beyond the immediate context of the pandemic. Such research could assess the long-term benefits and challenges associated with telehealth, shedding light on its effectiveness, sustainability, and impact. Despite these limitations, this study is the first to collect data on ICT use in maternity care from institutions in Latin America and the Caribbean at such a large scale. Data from this study contribute to our understanding of the prevalence of ICT use, the technologies used, the services provided, and the factors affecting their use and can inform the design of interventions to improve maternity care through the integration of digital health solutions.

Implications for practice

The integration of ICTs in maternity care is a promising strategy in Latin America and the Caribbean. Considerations for their adoption include the level of complexity of the intervention and the cost and availability of the technology required to implement it. Other considerations include the facility's staffing levels, the digital infrastructure, and institutional workflows and protocols, as well as the existing national regulatory framework. Many of the interventions we found relied on the phone, a widely available technology that many people know how to use. However, implementing any innovation requires substantial planning, coordination, and training. In our study, interviewees reported that apparently simple interventions faced challenges. For example, facilities trying to implement advanced scheduling of antenatal care visits by phone

encountered insufficient human resources to schedule and/or reschedule visits, and to coordinate care given no-shows and patients without appointments. The more complex the system or platform, the more resources and time are needed for its development and implementation. Health data sharing, for instance, requires systems that are secure and interoperable across institutions.

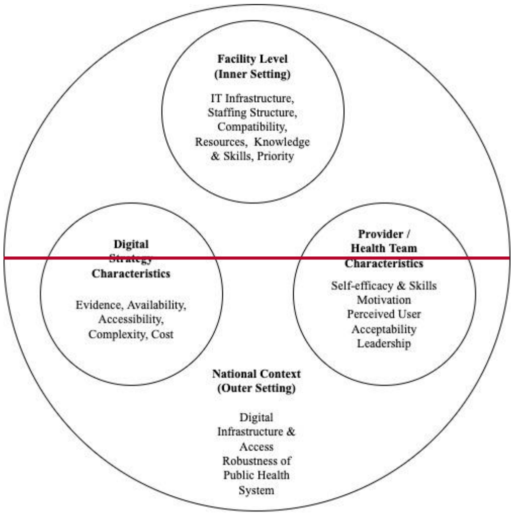

Figure 3. Key Factors Affecting the Use of ICTs in Maternal Health from an Implementation Science Lens

For institutions ready to expand the use of telehealth, PAHO has developed a tool for institutions to self-assess their readiness to use ICTs, identify gaps, and prioritize investments in improving data use and data collection [33]. The tool provides guidance to analyze telehealth readiness in terms the infrastructure, human resources and capacity building, and organizational cultural transformation needed to capture, analyze, and use health data for continuous quality

improvement in health.[30]. The tool provides guidance on assessing telehealth readiness of the infrastructure, human resources and capacity, and the use health data to improve care quality. Further, WHO has also issued guidelines for facilities seeking to incorporate telehealth for systems strengthening [2931].

Field Code Changed

Strengths and limitations

Formatted: Font: 16 pt

This study findings should be considered in the light of some limitations. Despite efforts to obtain a representative sample of facilities in each county, it should be noted that facilities in distant areas and areas with low internet connectivity were harder to reach. This bias is particularly pertinent to the subject of this analysis, as we may have over-represented the use of ICTs in maternal health. Further, response rates varied by country, and were particularly low in the Dominican Republic. To mitigate this bias, in some cases national consultants travelled to priority hard-to-reach areas to administer the survey in person and in others called potential respondents via phone or WhatsApp and administered the survey verbally. The survey was validated with healthcare providers in each country. However, there may have been differences among respondents in the interpretation of the survey questions, thus introducing measurement errors. The mixed methods study focused on the experiences of health providers and administrators primarily in public sector facilities, that serve the highest-need populations. Future research could focus on user experiences, such the satisfaction, acceptability and cultural appropriateness of using ICTs to receive maternal healthcare, and more generally healthcare remotely. Another limitation of this study is the cross-sectional design, which captured the experiences of facilities at a specific moment in time. The responses illustrated how ICTs were used at the time of the survey and changes in their use during the pandemic. However, it was not

~~clear how widely these had been used prior to the pandemic or the extent to which they would continue to be used after the resolution of the sanitary crisis. Future longitudinal research would provide valuable insights into the continuity and evolution of ICT use in maternal healthcare beyond the immediate context of the pandemic. Such research could assess the long-term perceived benefits and challenges associated with telehealth, shedding light on its effectiveness, sustainability, and impact on maternal healthcare provision. By examining telehealth interventions over an extended period, researchers could capture changes in implementation, user experiences, and outcomes, thereby informing future strategies and policy decisions related to telehealth in the context of maternal health.~~

Conclusions

Formatted: Font: 18 pt

In this mixed methods study, we found that many facilities throughout the Latin American and Caribbean region ~~have~~ incorporated some form of ICTs in ~~maternal healthcare.~~maternity care. Respondents considered ICTs as a promising tool to ~~enhance the effectiveness of maternal health services and~~ improve ~~accessibility for the population~~care quality and access. During the COVID-19 pandemic, many facilities turned to ICTs to connect with pregnant women, communicate with other providers, and manage patient flow to reduce congestion. Facilities relied on low-cost technologies that were ~~most~~ readily available. However, findings also highlighted that ICTs may exacerbate disparities in ~~maternal healthcare access.~~maternity care. In particular, primary care facilities in rural areas with deficient digital infrastructure were ~~at risk of being~~disproportionately excluded from the benefits of telehealth.

Furthermore, the study findings revealed that while there were examples of bilateral investments to develop complex digital platforms, they were not sustainable in the long run. Instead, systems led and coordinated by the government, as part of concerted national efforts to expand the use of ICTs in healthcare, alongside the development of the national digital infrastructure, were found to be the most sustainable and promising for the future.

Overall, more research is needed to understand which digital strategies will persist post-COVID and their impact on access to care and maternal and perinatal outcomes. Research focused on understanding implementation aspects of digital strategies~~,~~ will be particularly useful in promoting scale-up and increasing uptake. Examining the long-term use and outcomes of these digital strategies will provide valuable insights into their effectiveness and potential for addressing existing health disparities.

Acknowledgments

Formatted: Font: 18 pt

The authors wish to thank the following individuals for their support in outreach and national data collection: Andrea Mirella Arpita Rojas, Carlos Ayala, María Victoria Bertolino, Alana Browne, Guillermo Carroli, Evelyne Ancion Degraff, Alexia Escóbar Vásquez, Xiomara Fernández, Rosalinda Hernández, Elodia Jara, Haydée Padilla, Margarita Pérez, Bertha Pooley, Kenia Mariel Ramos Aguilar, Nora Redondo, Catherina Rodriguez, Mirian Rojas, Erick Rousselin, Claudio Sosa, Luis Urbina, Nora Edith Valdivia Soto, Janice Woolford, as well as all the interviewees in ministries of health and health facilities for their time and invaluable contributions.

References

Formatted: Font: 18 pt

1. World Health Organization. Trends in maternal mortality 2000 to 2020: estimates by WHO, UNICEF, UNFPA, World Bank Group and UNDESA/Population Division. Geneva: WHO; 2023. https://www.who.int/publications/i/item/9789240068759
2. Cuevas L. Informe sobre los datos actualizados y análisis de indicadores clave de salud materna y razón de mortalidad materna de países de Latino América y el Caribe, periodo 2015-2021 [Report on updated data and analysis of key maternal health indicators and maternal mortality ratio in Latin American and Caribbean countries, period 2015-2021]. Panama City, Panama: Regional Task Force for the Reduction of Maternal Mortality; 2023.
3. Maza-Arnedo F, Paternina-Caicedo A, Sosa CG, de Mucio B, Rojas-Suarez J, Say L, et al. Maternal mortality linked to COVID-19 in Latin America: Results from a multi-country collaborative database of 447 deaths. The Lancet Regional Health – Americas. 2022;12. doi: 10.1016/j.lana.2022.100269
4. Chou D, Daelmans B, Jolivet RR, Kinney M, Say L. Ending preventable maternal and newborn mortality and stillbirths. BMJ : British Medical Journal. 2015;351:h4255. doi: 10.1136/bmj.h4255
5. World Health Organization. WHA58.28 eHealth Ninth plenary meeting, 25 May 2005 – Committee A, seventh report), (2005). Available from: https://apps.who.int/gb/ebwha/pdf_files/WHA58/WHA58_28-en.pdf
6. World Health Organization. Global difusion of eHealth: making universal health coverage achievable. Report of the third global survey on eHealth. Geneva, Switzerland: WHO; 2016. Available from: https://apps.who.int/iris/handle/10665/252529

7. Pan American Health Organization. CD51/13: Strategy and Plan of Action on eHealth, Washington D.C.: PAHO; 2011. Available from: https://iris.paho.org/handle/10665.2/7663

8. Pan American Health Organization. Framework for the Implementation of a Telemedicine Service. Washington, DC: PAHO; 2016. Available from: https://iris.paho.org/handle/10665.2/28414

9. Pan American Health Organization. eHealth in the Region of the Americas: breaking down the barriers to implementation. Results of the World Health Organization's Third Global Survey on eHealth. Washington, D.C.: PAHO; 2016. Available from: https://iris.paho.org/handle/10665.2/31286

10. Galle A, Semaan A, Huysmans E, Audet C, Asefa A, Delvaux T, et al. A double-edged sword-telemedicine for maternal care during COVID-19: findings from a global mixed-methods study of healthcare providers. BMJ Glob Health. 2021;6(2). doi: 10.1136/bmjgh-2020-004575

11. Cantor AG, Jungbauer RM, Totten AM, Tilden EL, Holmes R, Ahmed A, et al. Telehealth Strategies for the Delivery of Maternal Health Care : A Rapid Review. Ann Intern Med. 2022;175(9):1285-97. doi: 10.7326/M22-0737

12. Peahl AF, Smith RD, Moniz MH. Prenatal care redesign: creating flexible maternity care models through virtual care. American journal of obstetrics and gynecology. 2020;223(3):389.e1-.e10.

13. Palmer KR, Tanner M, Davies-Tuck M, Rindt A, Papacostas K, Giles ML, et al. Widespread implementation of a low-cost telehealth service in the delivery of antenatal care during the COVID-19 pandemic: an interrupted time-series analysis. The Lancet. 2021;398(10294):41-52. [redacted]

14. Pan American Health Organization. Technical study on the availability of digital solutions to improve maternal and sexual and reproductive health. Washington, D.C.: PAHO; 2021.

15. Meza-Santibañez L, H., Novoa R, Torres-Osorio J, Jáuregui-Canchari V, Rodríguez-Hilario N, Guevara-Ríos E, et al. Implementación de un modelo mixto de atención prenatal, presencial y virtual durante la pandemia COVID-19, en el Instituto Nacional Materno Perinatal en Lima, Perú [Implementation of a mixed model of prenatal, face-to-face, and virtual care during the COVID-19 pandemic, at the Instituto Nacional Materno Perinatal in Lima, Peru]. Revista Peruana de Ginecología y Obstetricia. 2021;67(2). http://www.scielo.org.pe/scielo.php?script=sci_arttext&pid=S2304-51322021000200004

16. Townsend R, Chmielewska B, Barratt I, Kalafat E, van der Meulen J, Gurol-Urganci I, et al. Global changes in maternity care provision during the COVID-19 pandemic: A systematic review and meta-analysis. eClinicalMedicine. 2021;37:100947. [redacted]

17. Hsieh HF, Shannon SE. Three approaches to qualitative content analysis. Qual Health Res. 2005;15(9):1277–88. doi: 10.1177/1049732305276687

18. International Telecommunication Union (ITU). Digital Development Dashboard Geneva, Switzerland2023. Available from: https://www.itu.int/en/ITU-D/Statistics/Dashboards/Pages/Digital-Development.aspx.

19. World Bank. World Bank country classifications by income level, 2022-2023 2022. Available from: https://blogs.worldbank.org/opendata/new-world-bank-country-classifications-income-level-2022-2023.

20. Broadband Commission for Sustainable Development. 2025 Targets: Connecting the Other Half. Available from: https://www.broadbandcommission.org/broadband-targets/.

21. Nilsen P, Birken SA, ed. Handbook on implementation science. Cheltenham: Edward Elgar Publishing; 2020.

22. Damschroder LJ, Reardon CM, Widerquist MAO, Lowery J. The updated Consolidated Framework for Implementation Research based on user feedback. Implementation Science. 2022;17(1):75. doi: 10.1186/s13012-022-01245-0

23. Pérez-Lu JE, Iguiñiz Romero R, Bayer AM, García PJ. Reduciendo las inequidades en salud y mejorando la salud materna mediante la mejora de los sistemas de información en salud: Wawared Perú [Wawared Peru: reducing health inequities and improving maternal health by improving information systems in health]. Rev Peru Med Exp Salud Publica. 2015;32(2):373-7.

24. Argentina. Ministerio de Salud. Qué es la Telesalud. [What is telehealth?] Argentina.gob.ar, 2020. Available from: https://www.argentina.gob.ar/salud/telesalud/definicion.

25. Colombia. Ministerio de Salud y Protección Social. Lineamientos provisionales para la atención en salud de las gestantes, recién nacidos y para la lactancia materna, en el contexto de la pandemia de covid-19 en Colombia [Provisional guidelines for health care for pregnant women, newborns and for breastfeeding, in the context of the covid-19 pandemic in Colombia]. Bogotá; MSPS; junio 2020. Available from: https://www.minsalud.gov.co/Ministerio/Institucional/Procesos%20y%20procedimientos/GIPS14.pdf

26. World Health Organization & World Bank. (2017). Tracking universal health coverage: 2017 global monitoring report. World Health Organization. Available from: https://apps.who.int/iris/handle/10665/259817.

27. Madubuonwu J, Mehta P. How Telehealth Can be Used to Improve Maternal and Child Health Outcomes: A Population Approach. Clin Obstet Gynecol. 2021;64(2):398-406. doi: 10.1097/GRF.0000000000000610

28. Bouabida K, Lebouché B, Pomey MP. Telehealth and COVID-19 Pandemic: An Overview of the Telehealth Use, Advantages, Challenges, and Opportunities during COVID-19 Pandemic. Healthcare (Basel). 2022 Nov 16; 10(11):2293. doi: 10.3390/healthcare10112293

29. World Health Organization. Recommendations on Digital Interventions for Health System Strengthening. WHO guideline. Geneva: World Health Organization; 2019. Available from: https://www.who.int/publications/i/item/9789241550505

30. Beuermann DW, Anta R, Curioso W, Garcia P, Maffioli A, Perez Lu J, et al. Determinants and effects of prenatal care services: evidence from a mobile phone RCT. Washington, D.C.: Inter-American Development Bank; 2014. Available from: https://issuu.com/bid-sph/docs/spd_04_17_14

31. Escobar MF, Echavarria MP, Vasquez H, Nasner D, Ramos I, Hincapié MA, et al. Experience of a telehealth and education program with maternal and perinatal outcomes in a low-resource region in Colombia. BMC Pregnancy Childbirth. 2022;22(1):604. doi: 10.1186/s12884-022-04935-1

32. Camacho-Leon G, Faytong-Haro M, Carrera K, Molero M, Melean F, Reyes Y, et al. A Narrative Review of Telemedicine in Latin America during the COVID-19 Pandemic. Healthcare (Basel). 2022;10(8):1361. doi: 10.3390/healthcare10081361

33. Pan American Health Organization. Rapid Assessment Tool for Critical Data Gathering. Washington, D.C.: PAHO; 2021. Available from: https://iris.paho.org/handle/10665.2/53614

1. World Health Organization. Trends in maternal mortality 2000 to 2020: estimates by WHO, UNICEF, UNFPA, World Bank Group and UNDESA/Population Division. Geneva: World Health Organization; 2023. Available from: https://www.who.int/publications/i/item/97892400687592.

2. Cuevas L. Informe sobre los datos actualizados y análisis de indicadores clave de salud materna y razón de mortalidad materna de países de Latino América y el Caribe, periodo 2015-2021 [Report on updated data and analysis of key maternal health indicators and maternal mortality ratio in Latin American and Caribbean countries, period 2015-2021]. Panama City: Regional Task Force for the Reduction of Maternal Mortality; 2023.

3. Maza-Arnedo F, Paternina-Caicedo A, Sosa CG, de Mucio B, Rojas-Suarez J, Say L, et al. Maternal mortality linked to COVID-19 in Latin America: Results from a multi-country collaborative database of 447 deaths. Lancet Reg Health Am. 2022;12:100269. doi:10.1016/j.lana.2022.100269.

4. Chou D, Daelmans B, Jolivet RR, Kinney M, Say L; Every Newborn Action Plan (ENAP) and Ending Preventable Maternal Mortality (EPMM) working groups. Ending preventable maternal and newborn mortality and stillbirths. BMJ. 2015 Sep 14;351:h4255. doi: 10.1136/bmj.h4255.

5. World Health Organization. WHA58.28 eHealth Ninth plenary meeting, 25 May 2005 – Committee A, seventh report), (2005). Available from: https://apps.who.int/gb/ebwha/pdf_files/WHA58/WHA58_28-en.pdf

6. World Health Organization. Global difusion of eHealth: making universal health coverage achievable. Report of the third global survey on eHealth. Geneva: WHO; 2016. Available from: https://apps.who.int/iris/handle/10665/252529

7. Pan American Health Organization. CD51/13: Strategy and plan of action on eHealth, Washington D.C.: PAHO; 2011. Available from: https://iris.paho.org/handle/10665.2/7663

8. Pan American Health Organization. Framework for the implementation of a telemedicine service. Washington, D.C.: PAHO; 2016. Available from: https://iris.paho.org/handle/10665.2/28414

9. Pan American Health Organization. eHealth in the Region of the Americas: breaking down the barriers to implementation. Results of the World Health Organization's Third Global Survey on eHealth. Washington, D.C.: PAHO; 2016. Available from: https://iris.paho.org/handle/10665.2/31286

10. Jung J, Katz R. Impacto del COVID-19 en la digitalización de América Latina [Impact of COVID-19 on the digitalization of Latin America]. Santiago: Economic Commision for Latin America and the Caribbean; 2023. Available from: https://repositorio.cepal.org/server/api/core/bitstreams/cf05ce4b-b465-4740-86a1-6b707267e99b/content

11. Townsend R, Chmielewska B, Barratt I, Kalafat E, van der Meulen J, Gurol-Urganci I, et al. Global changes in maternity care provision during the COVID-19 pandemic: A systematic review and meta-analysis. eClinicalMedicine. 2021;37:100947. doi: 10.1016/j.eclinm.2021.100947

12. Galle A, Semaan A, Huysmans E, Audet C, Asefa A, Delvaux T, et al. A double-edged sword-telemedicine for maternal care during COVID-19: findings from a global mixed-methods study of healthcare providers. BMJ Glob Health. 2021;6(2). doi: 10.1136/bmjgh-2020-004575

13. Cantor AG, Jungbauer RM, Totten AM, Tilden EL, Holmes R, Ahmed A, et al. Telehealth strategies for the delivery of maternal health care: A rapid review. Ann Intern Med. 2022;175(9):1285-97. doi: 10.7326/M22-0737

14. Peahl AF, Smith RD, Moniz MH. Prenatal care redesign: creating flexible maternity care models through virtual care. Am J Obstet Gynecol. 2020;223(3):389.e1-389.e10. doi: 10.1016/j.ajog.2020.05.029.

15. Meza-Santibañez LH, Novoa R, Torres-Osorio J, Jáuregui-Canchari V, Rodríguez-Hilario N, Guevara-Ríos E, et al. Implementation of a mixed model of in person and virtual prenatal care during the COVID-19 pandemic at the National Maternal Perinatal Institute in Lima, Peru. Revista Peruana de Ginecología y Obstetricia. 2021;67(2). doi: 10.31403/rpgo.v67i2313

16. Escobar MF, Echavarria MP, Vasquez H, Nasner D, Ramos I, Hincapié MA, et al. Experience of a telehealth and education program with maternal and perinatal outcomes in a low-resource region in Colombia. BMC Pregnancy Childbirth. 2022;22(1):604. doi: 10.1186/s12884-022-04935-1

17. Pan American Health Organization. Technical study on the availability of digital solutions to improve maternal and sexual and reproductive health. Washington, D.C.: PAHO; 2021.

18. Hsieh HF, Shannon SE. Three approaches to qualitative content analysis. Qual Health Res. 2005;15(9):1277-88. doi: 10.1177/1049732305276687

19. International Telecommunication Union (ITU). Digital Development Dashboard. Geneva: ITU; 2023. Available from: https://www.itu.int/en/ITU-D/Statistics/Dashboards/Pages/Digital-Development.aspx.

20. World Bank. World Bank country classifications by income level, 2022-2023. Geneva: World Bank; 2022. Available from: https://blogs.worldbank.org/opendata/new-world-bank-country-classifications-income-level-2022-2023.

21. Broadband Commission for Sustainable Development. 2025 Targets: Connecting the Other Half. Available from: https://www.broadbandcommission.org/broadband-targets/.

22. Pérez-Lu JE, Iguiñiz Romero R, Bayer AM, García PJ. Reduciendo las inequidades en salud y mejorando la salud materna mediante la mejora de los sistemas de información en salud: Wawared Perú [Wawared Peru: reducing health inequities and improving maternal health by improving information systems in health]. Rev Peru Med Exp Salud Publica. 2015;32(2):373-377.

23. Argentina. Ministerio de Salud. Qué es la Telesalud. [What is telehealth?] Argentina.gob.ar, 2020. Available from: https://www.argentina.gob.ar/salud/telesalud/definicion.

24. Colombia. Ministerio de Salud y Protección Social (MSPS). Lineamientos provisionales para la atención en salud de las gestantes, recién nacidos y para la lactancia materna, en el contexto de la pandemia de covid-19 en Colombia [Interim guidelines for health care for pregnant women, newborns and breastfeeding, in the context of the Covid-19 pandemic in Colombia]. Bogota; MSPS; junio 2020. Available from: https://www.minsalud.gov.co/Ministerio/Institucional/Procesos%20y%20procedimientos/GIPS14.pdf

25. World Health Organization, World Bank. Tracking universal health coverage: 2017 global monitoring report. WHO and World Bank; 2017. Available from: https://apps.who.int/iris/handle/10665/310924

26. Madubuonwu J, Mehta P. How telehealth can be used to improve maternal and child health outcomes: a population approach. Clin Obstet Gynecol. 2021;64(2):398-406. doi:10.1097/GRF.0000000000000061027.

27. Bouabida K, Lebouché B, Pomey MP. Telehealth and COVID-19 pandemic: an overview of the telehealth use, advantages, challenges, and opportunities during COVID-19 pandemic. Healthcare (Basel). 2022;10(11):2293. doi:10.3390/healthcare1011229328.

28. Aizenberg M. Regulatory framework for telemedicine: current status and next steps. Washington, D.C.: Inter-American Development Bank; 2022. Available from: https://publications.iadb.org/publications/english/viewer/Regulatory-Framework-for-Telemedicine-Current-status-and-Next-Steps.pdf

29. Camacho-Leon G, Faytong-Haro M, Carrera K, Molero M, Melean F, Reyes Y, et al. A narrative review of telemedicine in Latin America during the COVID-19 pandemic. Healthcare (Basel). 2022;10(8). doi: 10.3390/healthcare10081361

30. Pan American Health Organization. Rapid assessment tool for critical data gathering. Washington, D.C.: PAHO; 2021. Available from: https://iris.paho.org/handle/10665.2/53614

31. World Health Organization. WHO guideline recommendations on digital interventions for health system strengthening. Geneva: WHO; 2019. Available from: https://iris.who.int/bitstream/handle/10665/311941/9789241550505-eng.pdf?ua=1

Supporting information

S1 Table. Sampling frame by country.

S2 Table. Response rate by country.

S3 Table. Use of ICTs for abortion counseling by country.

S1 Text. Interview guide.

S2 Text. Online survey questionnaire.

S1 Graph. Future use of ICTs among respondents who used them (n = 1459).

Formatted: Caption, Line spacing: single

Formatted: Font: Bold, Font color: Text 1

PONE-D-23-21638
Digital health and the promise of equity in maternity care: A mixed methods multi-country assessment on the use of information and communication technologies in healthcare facilities in Latin America and the Caribbean

Point-by-point response to reviewers

Journal Requirements

1. Please ensure that your manuscript meets PLOS ONE's style requirements, including those for file naming. The PLOS ONE style templates can be found at https://journals.plos.org/plosone/s/file?id=wjVg/PLOSOne_formatting_sample_main_body.pdf and https://journals.plos.org/plosone/s/file?id=ba62/PLOSOne_formatting_sample_title_authors_affiliations.pdf

RESPONSE: We modified the manuscript in accordance to PLOS ONE’s style requirements.

2. Thank you for stating in your Funding Statement:
"This study was partially funded by Global Affairs Canada through an agreement with the Pan American Health Organization/the World Health Organization. Global Affairs Canada was not involved in the study design, collection, analysis, and interpretation of data, writing, and submission."

RESPONSE: We included an amended statement in the cover letter, where we also confirm that no additional funding was received for this study.

3. In your Data Availability statement, you have not specified where the minimal data set underlying the results described in your manuscript can be found. PLOS defines a study's minimal data set as the underlying data used to reach the conclusions drawn in the manuscript and any additional data required to replicate the reported study findings in their entirety. All PLOS journals require that the minimal data set be made fully available. For more information about our data policy, please see http://journals.plos.org/plosone/s/data-availability.

Upon re-submitting your revised manuscript, please upload your study’s minimal underlying data set as either Supporting Information files or to a stable, public repository and include the relevant URLs, DOIs, or accession numbers within your revised cover letter. For a list of acceptable repositories, please see http://journals.plos.org/plosone/s/data-availability#loc-recommended-repositories. Any potentially identifying patient information must be fully anonymized.

We will update your Data Availability statement to reflect the information you provide in your cover letter.

RESPONSE: The minimal data set has been uploaded to the OpenICPSR repository, and can be found in repository number: https://doi.org/10.3886/E195667V1.

4. Please include captions for your Supporting Information files at the end of your manuscript, and update any in-text citations to match accordingly. Please see our Supporting Information guidelines for more information: http://journals.plos.org/plosone/s/supporting-information.

RESPONSE: Separate supporting information files have been uploaded for each element, the in-text citations have been modified to meet PLOS ONE formatting standards, and a list of captions have been included at the end of the manuscript file.

Review Comments to the Author

Please note, the line numbers listed below are those in the revised manuscript with tracked changes document.

5. Review Comments to the Author

Reviewer #1: The manuscript described a very interesting issue in health area and approached a promissing issue in the present and future time. The main objective is describing the use of information and communication technology in maternal care assistence in nine Latin American countries.

There are some questions to the authors, which are:
-Which was the criteria to select these countries? Why was Brazil not included, as it is one of the biggest countries in Latin America?

RESPONSE: This is an important point as Brazil is the country with the highest absolute number of maternal deaths in the region. However, this study was part of a Pan American Health Organization program to improve access to primary care in Latin America and the Caribbean and only included the countries in which this program was implemented.

We addressed this in the methods (new text in italics):

“This is a cross-sectional mixed methods study of health facilities providing maternity care in 9 Latin American and Caribbean countries *that participated in a PAHO-led program to improve primary care access*” (lines 163-165)

-Is there any specific reason to explain the difference of response rate among countries, beyond internet affordability?

RESPONSE: Difference in response rates among countries probably depends on the survey dissemination strategy, as well as on internet coverage and the accessibility of equipment. I think the manuscript makes the case that a State that has historically invested in developing a digital infrastructure - as well as a robust public healthcare system – is key to ICT use, in general.

-Is there a difference in the use of ICT between the public and private service? What is the percentage of public and private services in each country?

RESPONSE: This is a great question. Private services probably do have a more developed ICT infrastructure because they have more resources. For example, in Peru, members of EsSalud, which is the government's paid health insurance for employed citizens, have more options to access telemedicine, than members of SIS, which is the government's free health insurance. This manuscript primarily focused on free public services for the most vulnerable populations who face the greatest difficulties accessing health care. Each country's health care systems is pretty fragmented. Determining the percentage of public versus public services available in each would be beyond the scope of this manuscript. However, it would be an interesting future study.

-Page 8: The authors described that "in 7 countries, interviews were conducted with maternal health...". The question is: in the other 2 countries, with whom the interview was conducted?

RESPONSE: This is a great question. We did not conduct qualitative data collection in two countries. We only conducted interviews in countries where we learned about programs in maternity care that used ICTs, so that we could gather more details about these.

We clarified this point in line 238 (added text in italics):

"In *the* 7 countries *where we found promising examples of use of ICTs in maternity care*…"

-In table 1 (page 12 and 13): why the authors included the column "Used ICTs (%by column)"? I suggest to exclude this column, as there are marked differences between the categories of each item (institution type, level of care and country).

RESPONSE: Thank you for this great suggestion. We removed the column.

-Review the numering of tables – there are 2 tables numbered as "1".

RESPONSE: We were referring to Table 1 twice, the first time to describe the types of institutions that responded to the survey and the other to describe the prevalence of ICT use. Since we modified the table and added a figure (new Fig 1), this is no longer an issue.

Reviewer #2: This is a good article including a massive survey conducted in the region. Please consider the following comments, that I structured in general and specific:

General comments
1. I didn't understand if the NYU IRB actually reviewed your submission and said it didn't need ethics approval, or if this was the general instruction that appear in its website. It does look that the informed consents that were used should have been approved by an ethics board.

RESPONSE: Thank you for this point. As per NYU publicly-available ethics committee guidelines, this study did not require submission to the IRB as it did not constitute human subjects' research. The information collected is not about individuals, but about organizations. The NYU IRB guidance is found here: Does Your Project Require an Application to the NYU IRB Office? Decision Tree #1. Available at: https://www.nyu.edu/content/dam/nyu/research/documents/IRB/IRBDecisionTree.pdf. Even though it didn't required IRB review, we followed ethical standards by using the consent to inform participants about how the information would be used.

Field Code Changed

2. I suggest the authors might review the English, and perhaps asking a native speaker to take a quick look at it. There are several examples in which the English could be improved (e.g., "maternal mortality is a grave public health").

RESPONSE: Thank you for your feedback. We have carefully copy edited the manuscript to ensure that there are no typos or grammatical errors.

3. The research questions and how the methods address them are a bit unclear from my perspective. RQ1 and 3 seems to be served by the survey and interviews, but it is not clear how RQ2 would be served by both methods.

RESPONSE: We included multiple questions both in the survey and interviews about the context in which ICTs were adopted, including when they were funded and adopted, among others, to help us understand if and how changes in ICT use occurred during COVID-19.

4. Additionally, it is not clear how the methods would be providing complementary perspectives to address the same questions (e.g., what is the difference of addressing RQ1 using the survey as opposed to using the interview results).

RESPONSE: The methods are complementary because whereas the survey allowed us to reach more institutions across the region and quantify the use of ICTs in the 9 countries assessed, the qualitative data allowed us to gather more nuanced information related to the impetus for ICT adoption, the context in which the adoption of ICTs occurred, the exact use of ICTs (impetus, team involved, intervention characteristics, etc.).

We explained this in lines 248-251:

"The information gathered from the different data collection methods was complementary: whereas the survey allowed us to collect quantitative data from many institutions, the qualitative data provided us nuanced information on the context in which specific interventions were implemented."

5. It would be important to specify a bit more RQ2, and what is understood by "shape the implementation"

RESPONSE: We appreciate the point and have modified the language in RQ2 to address this point (rephrased RQ2 in italics, line 156):

1) *Understand changes in the use of ICTs as a result of the COVID-19 pandemic*

6. I was completely confused when the CFIR part came in the methods, and the results/discussion. If you think you could structure the whole research (including the questionnaire, interview guide and key findings) using CFIR, then please do so. But how it is now, it seems a different paper, because it is not clear what research question is being answered with that analysis.

RESPONSE: We made significant changes to the introduction and discussion to address this issue; we reorganized the discussion and removed the sections related to the CFIR framework. Whereas we still believe that CFIR provides a useful framework to understand the adoption of innovations in a real-world setting, we agree that this content would best suit a separate paper.

7. By addressing the previous comment, you could also reduce the length of the discussion section which is too long for a paper.

RESPONSE: Thank you for the suggestion. Indeed, the manuscript is much shorter with the removal of the CFIR sections.

8. In the classification of institutions type, I am not sure about the difference between them (e.g., is a referral hospital not a health center?)

RESPONSE: A health center is a health facility that only provides primary level care, as compared to a hospital which can offer higher complexity health services. To avoid confusion, we clarified this point by changing the terminology to “primary care health center.”

9. One thing that wasn’t clear to me is how use of ICTs is measured. It might be important to clearly state that in the methods section as well as reflecting around this in the discussion section.

RESPONSE: ICT use was measured by the following survey question:

Does your facility use any information and communication technology (ICT) tools for maternal health care (e.g., phone calls, video calls, telemonitoring, text messaging, etc.)?

The answer options were: Yes, the same way as we did before the onset of the pandemic (1); Yes, more than before the pandemic (2); Yes, we started using ICT tools when the pandemic was already under way (3); and No (4).

We added this information to the methods:
“ICT use was assessed by the question “Does your facility use any information and communication technology (ICT) tools for maternal health care (e.g., phone calls, video calls, telemonitoring, text messaging, etc.)?” (Answer options: Yes, the same way as we did before the onset of the pandemic; Yes, more than before the pandemic; Yes, we started using ICT tools when the pandemic was already under way; and No). The variable was dichotomized Yes/No.” (lines 215-219)

10. I got confused in page 24 for the “institutional factors and strategies”. I don’t understand how they are not a subset of factors that are explainednin figure 1?

RESPONSE: This confusion is understandable. Figure 1 presented survey respondents’ perceptions of the factors that facilitated ICT use, whereas Table 3 are the actual strategies implemented by institutions to facilitate ICT use. To avoid confusion, we incorporated findings from the “institutional factors and strategies” section into the factors associated with ICT use.

Specific comments
Introduction
1. Not sure if eradicating maternal deaths is actually an objective. I tend to believe that the verb eradicating is normally used with diseases. Maybe it might be more relevant to use reducing maternal deaths.

RESPONSE: Thank you. We agree with the comment and changed the language to reducing. (line 67)

2. At the end of introduction, the COVID-19 pandemic is introduced at the end of this section, but seems very disconnected from the rest of the section.

RESPONSE: We appreciate this suggestion. We wove in the introduction to COVID-19 within the section presenting prior evidence on the use of ICTs in the region. The main point we were trying to make was that by creating barriers to accessing services in person, COVID-19 may have accelerated the adoption of ICTs. Thus, warranting this study on the use of ICTs in the post-pandemic period (lines 121-125).

3. It is important the the introducxtion could justify the research questions included. Reading the RQ1, it seems that could have been already answered by reference 14. What additional value is being added by this research piece by answering that research question?

RESPONSE: Reference 14 is a desk review of published evidence on the use of ICTs in sexual and reproductive health. However, the vast majority of health interventions carried out by health facilities in Latin America and the Caribbean are not documented. Thus, any study based solely on desk review is vastly limited. Primary data collection via survey and interviews provides a more robust picture of the situation of ICT use in the region.

We clarified this in the manuscript:

“A caveat of this study is that, since the majority of interventions carried out in facilities across Latin America and the Caribbean are not documented in the literature, a desk review provides only a partial picture of the reality on the ground..” (lines 138-141)

4. Additionally, authors should provide a more robust explanation of the data integration part of the mixed-methods. Are you using triangulation, what type of strategy?

RESPONSE: The survey findings were used to present quantitative data, primarily related to prevalence of ICT use, types of ICTs used, and services provided. The interviews served to learn more about specific practices and the context in which ICTs were used. As mentioned above, the methods are complementary because whereas the survey allowed us to reach more institutions and quantify the use of ICTs, the qualitative data allowed us to gather more nuanced information related to the impetus for ICT adoption, the context in which the adoption of ICTs occurred, the exact use of ICTs (purposes, technology used, team involved, etc.).

We explained this in lines 248-251:

"The information gathered from the different data collection methods was complementary: whereas the survey allowed us to collect quantitative data from many institutions, the qualitative data provided us nuanced information on the context in which specific interventions were implemented."

5. It would be important the the authors describe why these nine countries were selected, and why only in 7 were the interviews conducted.

RESPONSE: This is a great point. This study was part of a Pan American Health Organization program to improve access to primary care in Latin America and the Caribbean and only included the countries in which this program was implemented. We only conducted interviews in countries where we learned about programs in maternal health that used ICTs, so that we could learn more about these.

We addressed this in the methods (new text in italics):

"This is a cross-sectional mixed methods study of health facilities providing maternity care in nine Latin American and Caribbean countries *that participated in a PAHO-led program to improve primary care access*" (lines 164-165)

"In *the* 7 countries *where we found promising examples of use of ICTs in maternal health care*…" (line 238-239)

6. Reference 13 is not a review, as it is mentioned in page 6 line 124.

RESPONSE: Thank you for the observation. We removed that sentence and reworded that paragraph to better reflect the evidentiary sources. (lines 129-134)

Methods
1. I suggest the authors use clear subheadings following participants selection, data collection methods and data analysis, and clearly splitting the two parts of their study.

RESPONSE: Thank you for the suggestion. We separated the quantitative and qualitative descriptions of the study methods into sections.

2. Also, I believe this is a mixed-methods study that includes a cross-sectional study as a quantatitve component.

RESPONSE: This is both a mixed-methods study because it uses quantitative and qualitative data collection methods AND a cross-sectional study because there is no repeating or longitudinal data – data from each resondents was collected at one time point.

3. Page 8, line 159. Please use another paragraph when “in 7 countries” starts.

RESPONSE: This section was moved when we reorganized the methods. We made sure it starts a new paragraph. (line 238)

4. Could you please explain why you had duplicates and a confidence interval for the response rate?

RESPONSE: We had duplicates because some respondents started the online survey and stopped for one reason or another (e.g., their internet was cut, they were interrupted by something else), and then instead of continuing the survey, they restarted it. This only happened when they used a different device to try to complete the survey. We calculated the confidence interval of the mean response rate to account for random variance across countries.

We clarified: “Duplicates occurred when respondents continued working on unsubmitted surveys from a different device.” (lines 275-276)

5. Page 9, lines 190-192, all these type of information I am inclined to say that it is part of the results.

RESPONSE: These lines were moved to the beginning of the results section. (lines 275-279)

Results
1. Page 11 line 238: There is an error.

RESPONSE: Thank you for pointing this out. We changed to the actual percentage, 76%. (line 302)

2. I would suggest that one key issue mentioned is that 78% of the institutions were primary care, meaning that we don’t have much information about the uses of digital health in hospital.

RESPONSE: Thank you. We have included this point in the discussion.

3. Table 1 title: Please specify that these are the results from the survey.

RESPONSE: The title of the table was modified to include that these were the results from the survey (lines 304).

4. In table 1, I think the % by row is confusing because this is not a representative sample.

RESPONSE: Thank you for your suggestion. We removed the percentage by row column.

5. Table numbers are wrong.

RESPONSE: We were referring to Table 1 twice, the first time to describe the types of institutions that responded to the survey and the other to describe the prevalence of ICT use. Since we modified the table and added a figure (new Fig 1), this is no longer an issue.

6. In table 2, it might be good to have more details on the intersection between the 2 variables shown (e.g., what type of ICT was used for gestational disease management?).

RESPONSE: This is a good suggestion but cannot be answered with the current data. We could do crosstabs on the two variables, but that still wouldn't tell us specifically what types of ICTs were used for a specific condition such as gestational disease management.

7. Page 17, line 314-315. I think this is should be included in the methods

RESPONSE: Thank you for the suggestion. We moved this to the survey questionnaire section. (lines 215-217)

8. Table 3, could you also provide frequencies?
RESPONSES: Thank you the suggestion. We added the frequencies to Table 3.

Discussion
1. I didn't see strengths, while your survey was massive!

RESPONSE: Thank you! We added strengths to the Strengths and Limitations subsection (line 780).

2. I didn't see implications for practice either.

RESPONSE: Valuable point. We added a section on implications for practice in the discussion (starting in line 813).
