# Supplementary material for: Digital health and the promise of equity in maternity care: A mixed methods multi-country assessment on the use of information and communication technologies in healthcare facilities in Latin America and the Caribbean
Source: PLoS One. 2024 Feb 27;19(2):e0298902. doi: 10.1371/journal.pone.0298902 (PMC10898739; doi:10.1371/journal.pone.0298902)
Supplement: Attachment — Submitted filename: Response to Reviewers.docx [file pone.0298902.s007.docx]

PONE-D-23-21638

Digital health and the promise of equity in maternity care: A mixed methods multi-country assessment on the use of information and communication technologies in healthcare facilities in Latin America and the Caribbean

**Point-by-point response to reviewers**

**Journal Requirements**

1. Please ensure that your manuscript meets PLOS ONE's style requirements, including those for file naming. The PLOS ONE style templates can be found at
   <https://journals.plos.org/plosone/s/file?id=wjVg/PLOSOne_formatting_sample_main_body.pdf> and 
   <https://journals.plos.org/plosone/s/file?id=ba62/PLOSOne_formatting_sample_title_authors_affiliations.pdf>

RESPONSE: We modified the manuscript in accordance to PLOS ONE’s style requirements.

2. Thank you for stating in your Funding Statement: 
"This study was partially funded by Global Affairs Canada through an agreement with the Pan American Health Organization/the World Health Organization. Global Affairs Canada was not involved in the study design, collection, analysis, and interpretation of data, writing, and submission."

RESPONSE: We included an amended statement in the cover letter, where we also confirm that no additional funding was received for this study.

3. In your Data Availability statement, you have not specified where the minimal data set underlying the results described in your manuscript can be found. PLOS defines a study's minimal data set as the underlying data used to reach the conclusions drawn in the manuscript and any additional data required to replicate the reported study findings in their entirety. All PLOS journals require that the minimal data set be made fully available. For more information about our data policy, please see <http://journals.plos.org/plosone/s/data-availability>.

Upon re-submitting your revised manuscript, please upload your study’s minimal underlying data set as either Supporting Information files or to a stable, public repository and include the relevant URLs, DOIs, or accession numbers within your revised cover letter. For a list of acceptable repositories, please see <http://journals.plos.org/plosone/s/data-availability#loc-recommended-repositories>. Any potentially identifying patient information must be fully anonymized.

We will update your Data Availability statement to reflect the information you provide in your cover letter.

RESPONSE: The minimal data set has been uploaded to the OpenICPSR repository, and can be found in repository number: https://doi.org/10.3886/E195667V1.

4. Please include captions for your Supporting Information files at the end of your manuscript, and update any in-text citations to match accordingly. Please see our Supporting Information guidelines for more information: <http://journals.plos.org/plosone/s/supporting-information>. 

RESPONSE: Separate supporting information files have been uploaded for each element, the in-text citations have been modified to meet PLOS ONE formatting standards, and a list of captions have been included at the end of the manuscript file.

**Review Comments to the Author**

Please note, the line numbers listed below are those in the revised manuscript with tracked changes document.

5. Review Comments to the Author

Reviewer #1: The manuscript described a very interesting issue in health area and approached a promissing issue in the present and future time. The main objective is describing the use of information and communication technology in maternal care assistence in nine Latin American countries.

There are some questions to the authors, which are:
-Which was the criteria to select these countries? Why was Brazil not included, as it is one of the biggest countries in Latin America?

RESPONSE: This is an important point as Brazil is the country with the highest absolute number of maternal deaths in the region. However, this study was part of a Pan American Health Organization program to improve access to primary care in Latin America and the Caribbean and only included the countries in which this program was implemented.

We addressed this in the methods (new text in italics):

“This is a cross-sectional mixed methods study of health facilities providing maternity care in 9 Latin American and Caribbean countries *that participated in a PAHO-led program to improve primary care access*” (lines 163-165)

-Is there any specific reason to explain the difference of response rate among countries, beyond internet affordability?

RESPONSE: Difference in response rates among countries probably depends on the survey dissemination strategy, as well as on internet coverage and the accessibility of equipment. I think the manuscript makes the case that a State that has historically invested in developing a digital infrastructure - as well as a robust public healthcare system – is key to ICT use, in general.

-Is there a difference in the use of ICT between the public and private service? What is the percentage of public and private services in each country?

RESPONSE: This is a great question. Private services probably do have a more developed ICT infrastructure because they have more resources. For example, in Peru, members of EsSalud, which is the government’s paid health insurance for employed citizens, have more options to access telemedicine, than members of SIS, which is the government’s free health insurance. This manuscript primarily focused on free public services for the most vulnerable populations who face the greatest difficulties accessing health care. Each country’s health care systems is pretty fragmented. Determining the percentage of public versus public services available in each would be beyond the scope of this manuscript. However, it would be an interesting future study.

-Page 8: The authors described that “in 7 countries, interviews were conducted with maternal health...”. The question is: in the other 2 countries, with whom the interview was conducted?

RESPONSE: This is a great question. We did not conduct qualitative data collection in two countries. We only conducted interviews in countries where we learned about programs in maternity care that used ICTs, so that we could gather more details about these.

We clarified this point in line 238 (added text in italics):

“In *the* 7 countries *where we found promising examples of use of ICTs in maternity care*…”

-In table 1 (page 12 and 13): why the authors included the column “Used ICTs (%by column)”? I suggest to exclude this column, as there are marked differences between the categories of each item (institution type, level of care and country).

RESPONSE: Thank you for this great suggestion. We removed the column.

-Review the numering of tables – there are 2 tables numbered as “1”.

RESPONSE: We were referring to Table 1 twice, the first time to describe the types of institutions that responded to the survey and the other to describe the prevalence of ICT use. Since we modified the table and added a figure (new Fig 1), this is no longer an issue.

Reviewer #2: This is a good article including a massive survey conducted in the region. Please consider the following comments, that I structured in general and specific:

General comments
1. I didn’t understand if the NYU IRB actually reviewed your submission and said it didn’t need ethics approval, or if this was the general instruction that appear in its website. It does look that the informed consents that were used should have been approved by an ethics board.

RESPONSE: Thank you for this point. As per NYU publicly-available ethics committee guidelines, this study did not require submission to the IRB as it did not constitute human subjects’ research. The information collected is not about individuals, but about organizations. The NYU IRB guidance is found here: Does Your Project Require an Application to the NYU IRB Office? Decision Tree #1. Available at: <https://www.nyu.edu/content/dam/nyu/research/documents/IRB/IRBDecisionTree.pdf>. Even though it didn’t required IRB review, we followed ethical standards by using the consent to inform participants about how the information would be used.

2. I suggest the authors might review the English, and perhaps asking a native speaker to take a quick look at it. There are several examples in which the English could be improved (e.g., “maternal mortality is a grave public health”).

RESPONSE: Thank you for your feedback. We have carefully copy edited the manuscript to ensure that there are no typos or grammatical errors.

3. The research questions and how the methods address them are a bit unclear from my perspective. RQ1 and 3 seems to be served by the survey and interviews, but it is not clear how RQ2 would be served by both methods.

RESPONSE: We included multiple questions both in the survey and interviews about the context in which ICTs were adopted, including when they were funded and adopted, among others, to help us understand if and how changes in ICT use occurred during COVID-19.

4. Additionally, it is not clear how the methods would be providing complementary perspectives to address the same questions (e.g., what is the difference of addressing RQ1 using the survey as opposed to using the interview results).

RESPONSE: The methods are complementary because whereas the survey allowed us to reach more institutions across the region and quantify the use of ICTs in the 9 countries assessed, the qualitative data allowed us to gather more nuanced information related to the impetus for ICT adoption, the context in which the adoption of ICTs occurred, the exact use of ICTs (impetus, team involved, intervention characteristics, etc.).

We explained this in lines 248-251:

*“The information gathered from the different data collection methods was complementary: whereas the survey allowed us to collect quantitative data from many institutions, the qualitative data provided us nuanced information on the context in which specific interventions were implemented.”*

5. It would be important to specify a bit more RQ2, and what is understood by “shape the implementation”

RESPONSE: We appreciate the point and have modified the language in RQ2 to address this point (rephrased RQ2 in italics, line 156):

1) *Understand changes in the use of ICTs as a result of the COVID-19 pandemic*

6. I was completely confused when the CFIR part came in the methods, and the results/discussion. If you think you could structure the whole research (including the questionnaire, interview guide and key findings) using CFIR, then please do so. But how it is now, it seems a different paper, because it is not clear what research question is being answered with that analysis.

RESPONSE: We made significant changes to the introduction and discussion to address this issue; we reorganized the discussion and removed the sections related to the CFIR framework. Whereas we still believe that CFIR provides a useful framework to understand the adoption of innovations in a real-world setting, we agree that this content would best suit a separate paper.

7. By addressing the previous comment, you could also reduce the length of the discussion section which is too long for a paper.

RESPONSE: Thank you for the suggestion. Indeed, the manuscript is much shorter with the removal of the CFIR sections.

8. In the classification of institutions type, I am not sure about the difference between them (e.g., is a referral hospital not a health center?)

RESPONSE: A health center is a health facility that only provides primary level care, as compared to a hospital which can offer higher complexity health services. To avoid confusion, we clarified this point by changing the terminology to “primary care health center.”

9. One thing that wasn’t clear to me is how use of ICTs is measured. It might be important to clearly state that in the methods section as well as reflecting around this in the discussion section.

RESPONSE: ICT use was measured by the following survey question:

Does your facility use any information and communication technology (ICT) tools for maternal health care (e.g., phone calls, video calls, telemonitoring, text messaging, etc.)?

The answer options were: Yes, the same way as we did before the onset of the pandemic (1); Yes, more than before the pandemic (2); Yes, we started using ICT tools when the pandemic was already under way (3); and No (4).

We added this information to the methods:

“ICT use was assessed by the question “Does your facility use any information and communication technology (ICT) tools for maternal health care (e.g., phone calls, video calls, telemonitoring, text messaging, etc.)?” (Answer options: Yes, the same way as we did before the onset of the pandemic; Yes, more than before the pandemic; Yes, we started using ICT tools when the pandemic was already under way; and No). The variable was dichotomized Yes/No.” (lines 215-219)

10. I got confused in page 24 for the “institutional factors and strategies”. I don’t understand how they are not a subset of factors that are explainednin figure 1?

RESPONSE: This confusion is understandable. Figure 1 presented survey respondents’ perceptions of the factors that facilitated ICT use, whereas Table 3 are the actual strategies implemented by institutions to facilitate ICT use. To avoid confusion, we incorporated findings from the “institutional factors and strategies” section into the factors associated with ICT use.

Specific comments
Introduction
1. Not sure if eradicating maternal deaths is actually an objective. I tend to believe that the verb eradicating is normally used with diseases. Maybe it might be more relevant to use reducing maternal deaths.

RESPONSE: Thank you. We agree with the comment and changed the language to reducing. (line 67)

2. At the end of introduction, the COVID-19 pandemic is introduced at the end of this section, but seems very disconnected from the rest of the section.

RESPONSE: We appreciate this suggestion. We wove in the introduction to COVID-19 within the section presenting prior evidence on the use of ICTs in the region. The main point we were trying to make was that by creating barriers to accessing services in person, COVID-19 may have accelerated the adoption of ICTs. Thus, warranting this study on the use of ICTs in the post-pandemic period (lines 121-125).

3. It is important the the introducxtion could justify the research questions included. Reading the RQ1, it seems that could have been already answered by reference 14. What additional value is being added by this research piece by answering that research question?

RESPONSE: Reference 14 is a desk review of published evidence on the use of ICTs in sexual and reproductive health. However, the vast majority of health interventions carried out by health facilities in Latin America and the Caribbean are not documented. Thus, any study based solely on desk review is vastly limited. Primary data collection via survey and interviews provides a more robust picture of the situation of ICT use in the region.

We clarified this in the manuscript:

“A caveat of this study is that, since the majority of interventions carried out in facilities across Latin America and the Caribbean are not documented in the literature, a desk review provides only a partial picture of the reality on the ground..” (lines 138-141)

4. Additionally, authors should provide a more robust explanation of the data integration part of the mixed-methods. Are you using triangulation, what type of strategy?

RESPONSE: The survey findings were used to present quantitative data, primarily related to prevalence of ICT use, types of ICTs used, and services provided. The interviews served to learn more about specific practices and the context in which ICTs were used. As mentioned above, the methods are complementary because whereas the survey allowed us to reach more institutions and quantify the use of ICTs, the qualitative data allowed us to gather more nuanced information related to the impetus for ICT adoption, the context in which the adoption of ICTs occurred, the exact use of ICTs (purposes, technology used, team involved, etc.).

We explained this in lines 248-251:

*“The information gathered from the different data collection methods was complementary: whereas the survey allowed us to collect quantitative data from many institutions, the qualitative data provided us nuanced information on the context in which specific interventions were implemented.”*

5. It would be important the the authors describe why these nine countries were selected, and why only in 7 were the interviews conducted.

RESPONSE: This is a great point. This study was part of a Pan American Health Organization program to improve access to primary care in Latin America and the Caribbean and only included the countries in which this program was implemented. We only conducted interviews in countries where we learned about programs in maternal health that used ICTs, so that we could learn more about these.

We addressed this in the methods (new text in italics):

“This is a cross-sectional mixed methods study of health facilities providing maternity care in nine Latin American and Caribbean countries *that participated in a PAHO-led program to improve primary care access*” (lines 164-165)

“In *the* 7 countries *where we found promising examples of use of ICTs in maternal health care*…” (line 238-239)

6. Reference 13 is not a review, as it is mentioned in page 6 line 124.

RESPONSE: Thank you for the observation. We removed that sentence and reworded that paragraph to better reflect the evidentiary sources. (lines 129-134)

Methods
1. I suggest the authors use clear subheadings following participants selection, data collection methods and data analysis, and clearly splitting the two parts of their study.

RESPONSE: Thank you for the suggestion. We separated the quantitative and qualitative descriptions of the study methods into sections.

2. Also, I believe this is a mixed-methods study that includes a cross-sectional study as a quantatitve component.

RESPONSE: This is both a mixed-methods study because it uses quantitative and qualitative data collection methods AND a cross-sectional study because there is no repeating or longitudinal data – data from each resondents was collected at one time point.

3. Page 8, line 159. Please use another paragraph when “in 7 countries” starts.

RESPONSE: This section was moved when we reorganized the methods. We made sure it starts a new paragraph. (line 238)

4. Could you please explain why you had duplicates and a confidence interval for the response rate?

RESPONSE: We had duplicates because some respondents started the online survey and stopped for one reason or another (e.g., their internet was cut, they were interrupted by something else), and then instead of continuing the survey, they restarted it. This only happened when they used a different device to try to complete the survey. We calculated the confidence interval of the mean response rate to account for random variance across countries.

We clarified: “Duplicates occurred when respondents continued working on unsubmitted surveys from a different device.” (lines 275-276)

5. Page 9, lines 190-192, all these type of information I am inclined to say that it is part of the results.

RESPONSE: These lines were moved to the beginning of the results section. (lines 275-279)

Results
1. Page 11 line 238: There is an error.

RESPONSE: Thank you for pointing this out. We changed to the actual percentage, 76%. (line 302)

2. I would suggest that one key issue mentioned is that 78% of the institutions were primary care, meaning that we don’t have much information about the uses of digital health in hospital.

RESPONSE: Thank you. We have included this point in the discussion.

3. Table 1 title: Please specify that these are the results from the survey.

RESPONSE: The title of the table was modified to include that these were the results from the survey (lines 304).

4. In table 1, I think the % by row is confusing because this is not a representative sample.

RESPONSE: Thank you for your suggestion. We removed the percentage by row column.

5. Table numbers are wrong.

RESPONSE: We were referring to Table 1 twice, the first time to describe the types of institutions that responded to the survey and the other to describe the prevalence of ICT use. Since we modified the table and added a figure (new Fig 1), this is no longer an issue.

6. In table 2, it might be good to have more details on the intersection between the 2 variables shown (e.g., what type of ICT was used for gestational disease management?).

RESPONSE: This is a good suggestion but cannot be answered with the current data. We could do crosstabs on the two variables, but that still wouldn’t tell us specifically what types of ICTs were used for a specific condition such as gestational disease management.

7. Page 17, line 314-315. I think this is should be included in the methods

RESPONSE: Thank you for the suggestion. We moved this to the survey questionnaire section. (lines 215-217)

8. Table 3, could you also provide frequencies?
RESPONSES: Thank you the suggestion. We added the frequencies to Table 3.

Discussion
1. I didn’t see strengths, while your survey was massive!

RESPONSE: Thank you! We added strengths to the Strengths and Limitations subsection (line 780).

2. I didn’t see implications for practice either.

RESPONSE: Valuable point. We added a section on implications for practice in the discussion (starting in line 813).
