# Supplementary material for: Digital health and the promise of equity in maternity care: A mixed methods multi-country assessment on the use of information and communication technologies in healthcare facilities in Latin America and the Caribbean
Source: PLoS One. 2024 Feb 27;19(2):e0298902. doi: 10.1371/journal.pone.0298902 (PMC10898739; doi:10.1371/journal.pone.0298902)
Supplement: Attachment — Submitted filename: Response to reviewers_R2.docx [file pone.0298902.s009.docx]

PONE-D-23-21638R1

Digital health and the promise of equity in maternity care: A mixed methods multi-country assessment on the use of information and communication technologies in healthcare facilities in Latin America and the Caribbean

PLOS ONE

Dear Dr. Hasan,

Thank you for the opportunity to resubmit the above-mentioned manuscript. Below my point-by-point response to reviewers.

Best regards,

Ariadna

A rebuttal letter that responds to each point raised by the academic editor and reviewer(s). You should upload this letter as a separate file labeled 'Response to Reviewers'.

- A marked-up copy of your manuscript that highlights changes made to the original version. You should upload this as a separate file labeled 'Revised Manuscript with Track Changes'.
- An unmarked version of your revised paper without tracked changes. You should upload this as a separate file labeled 'Manuscript'.

Journal Requirements:

Please review your reference list to ensure that it is complete and correct. If you have cited papers that have been retracted, please include the rationale for doing so in the manuscript text, or remove these references and replace them with relevant current references. Any changes to the reference list should be mentioned in the rebuttal letter that accompanies your revised manuscript. If you need to cite a retracted article, indicate the article’s retracted status in the References list and also include a citation and full reference for the retraction notice.

RESPONSE: We have reviewed the reference list to ensure completeness.

6. Review Comments to the Author

Please use the space provided to explain your answers to the questions above. You may also include additional comments for the author, including concerns about dual publication, research ethics, or publication ethics. (Please upload your review as an attachment if it exceeds 20,000 characters)

Reviewer #2: Thank you for addressing all the previous comments. I think most of them were already addressed. I only have 2 follow-up points. I add my original point + your response in each:


4. Additionally, it is not clear how the methods would be providing complementary
perspectives to address the same questions (e.g., what is the difference of addressing
RQ1 using the survey as opposed to using the interview results).

RESPONSE: The methods are complementary because whereas the survey allowed
us to reach more institutions across the region and quantify the use of ICTs in the 9
countries assessed, the qualitative data allowed us to gather more nuanced
information related to the impetus for ICT adoption, the context in which the adoption of
ICTs occurred, the exact use of ICTs (impetus, team involved, intervention
characteristics, etc.).
We explained this in lines 248-251:
“The information gathered from the different data collection methods was
complementary: whereas the survey allowed us to collect quantitative data from many
institutions, the qualitative data provided us nuanced information on the context in
which specific interventions were implemented."

4. Additionally, authors should provide a more robust explanation of the data
integration part of the mixed-methods. Are you using triangulation, what type of
strategy?
RESPONSE: The survey findings were used to present quantitative data, primarily
related to prevalence of ICT use, types of ICTs used, and services provided. The
interviews served to learn more about specific practices and the context in which ICTs
were used. As mentioned above, the methods are complementary because whereas
the survey allowed us to reach more institutions and quantify the use of ICTs, the
qualitative data allowed us to gather more nuanced information related to the impetus
for ICT adoption, the context in which the adoption of ICTs occurred, the exact use of
ICTs (purposes, technology used, team involved, etc.).


Follow-up: I will connect both points because I think I have the same comment. Thanks for your explanation, but I feel that the complementarity of both methods is still unclear. You explained very clear how the quantitative and qualitative data contributes to answer the question, but it would be helpful to say the survey answered the question related to prevalence, types of ICTs, and services provided, while the qualitative data answered the more nuanced information. I think these should be separate research questions clearly specified at the end of the introduction. I say this because otherwise readers might think that you are answering the same question using different methodologies, and then triangulation or other mixed-methods data integration method is expected.

Just this minor point, but the rest looks good to me!

RESPONSE: We have addressed this request to clarify how quantitative and qualitative data informed the study by modifying the first study objective, as follows (next text in italics):

Describe the *prevalence of ICT use in maternity care, the types of ICTs used, and the services provided using ICTs* in a sample of health facilities in 9 countries of Latin America and the Caribbean (page 7)

And be adding the following description in the methods (page 8):

*“Survey data served primarily to answer questions related to the prevalence of ICT use, the types of ICTs used, and the services provided using ICTs, whereas qualitative data served to gain nuanced information about the context in which ICTs were employed, and about the barriers and facilitators to their adoption.”*

Reviewer #3: Title: Digital health and the promise of equity in maternity care: A mixed methods multicountry assessment on the use of information and communication technologies in
healthcare facilities in Latin America and the Caribbean
Abstract: Key study aim, methods and result of the study well presented.
Introduction: Detailed information on statement of problem, rational for the study clearly presented and study objectives are well presented.
Methods: Fairly well structured and described. However, the following observation need to be addressed
• Survey sampling - Please provide the inclusion and exclusion criteria for selecting the facilities.

RESPONSE: We specified the inclusion and exclusion criteria as follows (page 8_:

*“The study included health facilities that provided maternity care in the countries under consideration, and excluded those that did not provide such care.”*

• Was the sampling frame restricted to public health facilities? The sampling technique used for each of the countries are not mentioned.

RESPONSE: This was part of the negotiation with the country teams. Most countries restricted the survey to public health facilities, with the private sector only included in Guyana, Paraguay, and Peru. Column 2 of the S1 Table with the sampling frame was modified to include this detail by country. This detail was also added to the survey sampling description (pages 8-9):

*“The country teams decided whether to limit the survey to the public sector or not. Most countries focused on public sector facilities, while Guyana, Paraguay, and Peru included those in both the public and private sectors.”*

• Interviewees were maternal health unit directors or telehealth focal points - In facilities where they have both, who is selected for the interview? This is likely to be the case in a number of health facilities used. Is it focal points or head of the telehealth units that were interviewed?

RESPONSE: In addition to facility-level providers, we tried to interview both telehealth and maternal health focal points at the ministries of health. Whether we reached both, depended on the national context, and on who was available and willing to participate.
